# 3-Benzyl-Hexahydro-Pyrrolo[1,2-a]Pyrazine-1,4-Dione Extracted From *Exiguobacterium indicum* Showed Anti-biofilm Activity Against *Pseudomonas aeruginosa* by Attenuating Quorum Sensing

**DOI:** 10.3389/fmicb.2019.01269

**Published:** 2019-06-07

**Authors:** Vijay K. Singh, Avinash Mishra, Bhavanath Jha

**Affiliations:** Division of Biotechnology and Phycology, CSIR – Central Salt and Marine Chemicals Research Institute, Bhavnagar, India

**Keywords:** anti-biofilm, anti-quorum, biofilm, *Exiguobacterium*, *Pseudomonas*, quorum quenching, quorum sensing

## Abstract

Bacterial cell-to-cell communication promotes biofilm formation and can potentially lead to multidrug resistance development. Quorum sensing inhibition (QSI) is an effective and widely employed strategy against biofilm formation. The extract from *Exiguobacterium indicum* SJ16, a gram-positive bacterium, isolated from the rhizosphere of *Cyperus laevigatus* showed significant anti-quorum sensing activity (about 99%) against the reference *Chromobacterium violaceum* CV026 strain without exerting any antibacterial effect. The potentially active QSI compound identified in the SJ16 extract was 3-Benzyl-hexahydro-pyrrolo[1, 2-a]pyrazine-1,4-dione. The SJ16 extract containing this active compound showed significant anti-quorum sensing activity against a model quorum sensing bacterium strain *Pseudomonas aeruginosa* PAO1 and a clinical isolate *P. aeruginosa* PAH by preventing biofilm formation without attenuating the cell growth within the biofilm. More specifically, the SJ16 extract changed the topography and architecture of the biofilm, thus preventing bacterial adherence and further development of the biofilm. Furthermore, it decreased virulence factors (rhamnolipid and pyocyanin), the bacterial motility, as well as the elastase, and protease activities in *P. aeruginosa*. Microarray analysis revealed the differential expression of quorum sensing regulatory genes. Based on these results, we herein propose a hypothetical model, characterizing the role of this QSI agent in the transcriptional regulation of quorum sensing in *P. aeruginosa* PAO1, demonstrating that this compound has significant drug-development potential. Further research is required to delineate its possible applications in therapeutics in the context of biofilm forming bacterial infections.

## Introduction

The diseases caused by pathogenic bacteria are controlled, prevented, and treated with a number of antibiotics which inhibit essential bacterial processes, such as cell wall synthesis, DNA replication, or protein biosynthesis. Antibiotics have long become the commonplace in our effort to tackle infectious diseases (Chu et al., 2014). However, extensive use of these agents creates an evolutionary pressure on bacteria which, in many cases, leads to antibiotics resistance. Therefore, alternative strategies with low potential for resistance emergence are required in order to combat pathogenic multidrug resistant bacteria (Chu et al., 2014; Singh et al., 2016b). Quorum sensing (QS) is directly involved in pathogenesis of infectious disease, by regulating the biofilm formation, the production of multiple virulence factors, as well as the motility of bacteria (Singh et al., 2017). In QS system, molecular cascades regulate the gene expression and determine the fate of bacterial biofilms (Ganin et al., 2015). In this context, gram-negative bacteria communicate through small diffusible molecules, such as acyl homoserine lactones, whereas gram-positive bacteria use autoinducer peptides for their communication (Galloway et al., 2011).

Biofilms are comprised of single or multiple microbial species and develop on different biotic and abiotic surfaces. In most of the cases, mixed species biofilms are predominant. However, single species biofilms are commonly associated with medical equipment-related infections, especially dental plaque and medical implants (O’Toole et al., 2000). *Pseudomonas aeruginosa*, a gram-negative bacterium, is considered to be the most important species of biofilm-forming bacteria. It can develop biofilms on a variety of biotic and abiotic surfaces; especially in immunocompromised patients (Driscoll et al., 2007). Moreover, this bacterium shows resistance to most of the conventional antibiotics, because it can form a biofilm matrix, which protects bacterial cells. Therefore, eradicating such infections poses a significant challenge (Driscoll et al., 2007; Lee and Zhang, 2015). QS regulatory networks control the virulence factors and biofilm formation in *P. aeruginosa* (Lee and Zhang, 2015). Therefore, utilization of anti-QS strategies could prove to be a promising way to tackle *P. aeruginosa* infections.

The rhizosphere is a region of soil adjacent to the plant roots that inhabits a number of microbes and facilitates various plant-microbe and microbe-microbe interactions. Many rhizospheric bacteria prevent the development of soilborne pathogens, while at the same time they protect the associated plants by activating the induced systemic resistance (Berendsen et al., 2012). Importantly, the rhizosphere supports different bacterial communities that exert QS and quorum quenching activities. Christiaen et al. (2011) isolated 59 bacterial species from 16 different environmental samples including plant rhizospheres and water, and found that 41 of them had anti-QS properties. Furthermore, *Stenotrophomonas maltophilia* and *Delftia tsuruhatensis*, isolated from the rhizosphere of *Cyperus laevigatus* showed anti-QS and anti-biofilm activities against *P. aeruginosa* (Singh et al., 2013, 2017). Moreover, a bacterium from the family of the *Acinetobacter* spp., isolated from the cucumber rhizosphere, inhibited the growth of *Pseudomonas chlororaphis* and *Burkholderia glumae* during co-cultivation by degrading acyl-homoserine lactones (AHLs), produced by these bacteria (Kang et al., 2004). Other AHL producing and degrading bacteria were isolated from the tobacco rhizosphere, including two newly identified species, *Sphingopyxis witflariensis* and *Bosea thiooxidans*, belong to the *Bacillus* α-proteobacteria family (D’Angelo-Picard et al., 2005). A three bacterial consortium comprised of *Acinetobacter* sp., *Burkholderia* sp. and *Klebsiella* sp. was isolated from the ginger rhizosphere, demonstrating significant AHL degrading activities and growth-inhibiting capabilities against *P. aeruginosa* and the plant pathogen *Erwinia carotovora* without affecting their planktonic growth (Chan et al., 2011). Last but not least, the AHL degrading bacterial consortia isolated from the potato rhizosphere showed significant biocontrol activity against *Pectobacterium atrosepticum* (Cirou et al., 2007). In most of these earlier studies, the above mentioned bacteria were isolated and characterized for their anti-QS properties; however, to the best of our knowledge there is no report, so far, on the isolation and characterization of the active compounds from these bacteria.

In the present study, a bacterium *Exiguobacterium indicum* SJ16 was isolated from the rhizosphere of a monocot *C. laevigatus*, amply growing in the coastal saline area and was tested for its anti-QS and anti-biofilm potential. The active fraction was collected, identified, and regulatory key genes were studied to elucidate a possible QSI mechanism.

## Materials and Methods

### Isolation and Molecular Identification of Bacteria

Bacteria from the rhizosphere of *C. laevigatus* L. from the natural habitat of Bhavnagar, India (Latitude N 21°45.124”, Longitude E 72°13.579”) were isolated. Bacteria were screened by plate based bioassay, and two were found positive for anti-QS activity (Singh et al., 2017). The bacterial isolate SJ16, which showed anti-QS (but not antibacterial) activity was selected for further characterization. Genomic DNA of the bacterium was isolated, and the 16S rRNA gene was amplified by universal primers fD1-5′-AGA GTT TGA TCC TGG CTC AG -3′ and rP2-5′-ACG GCT ACC TTG TTA CGA CTT -3′ using optimized PCR conditions (Weisburg et al., 1991). The amplified PCR product was purified, sequenced (at Macrogen Inc., South Korea), analyzed and submitted to the NCBI GenBank. The phylogenetic analysis was performed using MEGA version 6.0 (Tamura et al., 2013), and a phylogenetic tree was inferred by neighbor-joining methods (Saitou and Nei, 1987). Bootstrap analysis was performed, and maximum composite likelihood algorithms were used for the determination of the evolutionary distances (Felsenstein, 1985; Tamura et al., 2004).

### Fatty Acid Methyl Ester Profiling of Bacteria

Fatty acid methyl ester (FAME) profiling of *E. indicum* SJ16 was carried out using Microbial Identification System coupled with gas chromatography (MIDI, Microbial ID; GC system-6850, Agilent Technologies, United States). The bacterium was grown for 24 h at 30°C on Tryptic soy yeast agar, FAMEs were prepared using MIDI manual, and peaks were identified with RTSBA6 6.10 database (Jha et al., 2015).

### Preparation of the Bacterial Extract, Fractionation, and Identification of the Active Compound

Bacterial culture (*E. indicum* SJ16) was grown for 48 h at 30°C in 500 ml of nutrient broth, kept in an incubator shaker with agitation at180 rpm. The culture was centrifuged for 15 min at 10,000 × *g* (4°C), the supernatant was collected, and filtered through 0.22 μm filter for the complete removal of remaining bacterial cells. The supernatant was extracted with ethyl acetate (equal volume), evaporated to dryness under vacuum and the dried residue finally dissolved in methanol.

The methanol extract of SJ16 was further fractionated by solid phase extraction method using different cartridges including anion exchanger DAE, cation mixed Plexa PCX, polar SI, and non-polar C18 (Agilent, United States). Each fraction was tested for quorum sensing inhibition (QSI) activity (Singh et al., 2017). The positive fraction (collected through the C18 cartridge with 40% methanol) showing a maximum zone of QSI was used for the subsequent studies and was also subjected to GC-MS (GC-2010, Shimadzu, Japan) analysis. The probable active compound was identified by comparing the mass spectra with the reference spectra library. The mass of the probable active compound was further confirmed by electrospray ionization mass spectrometry (ESI-MS; Q-Tof micro TM, Micromass, United Kingdom).

### Anti-quorum Sensing and Anti-biofilm Activities

The anti-QS activity was tested (i) by a plate-based bioassay using *Chromobacterium violaceum* CV026 as a tester strain, methanol as a negative control and cinnamaldehyde (Sigma-Aldrich, United States) as a positive control, and (ii) by quantifying the violacein production. Both the plate-based bioassay and the violacein quantification, were performed as per our previously optimized methods (Singh et al., 2013).

The anti-biofilm formation assay was performed with different concentrations of bacterial (strain SJ16) extracts (0.2, 0.4, 0.6, 0.8, 1.0, and 1.2 mg/ml) using two *P. aeruginosa* strains, PAO1 as well as the PAH clinical isolate (provided as a courtesy from the Government Medical College, Bhavnagar; Goswami et al., 2011). Briefly, 200 μl bacterial culture (OD_600 nm_ = 0.1) dilute from overnight grown PAO1 and PAH cultures were added to a 96-well polystyrene microtiter plate with different concentrations of the SJ16 extracts. The plates were allowed to grow for 24 h (at 37°C with 100 rpm), and growth was measured at 600 nm. The biofilm formation was assayed using our previously optimized crystal violet staining method (Kavita et al., 2014). The experiments were performed three times with five replicates each time.

### Fluorescence Microscopy

*Pseudomonas aeruginosa* (strains PAO1 and PAH) were grown in a 24 well polystyrene plate with or without SJ16 extract to assess the development of biofilm on a sterilized glass coverslip (11 mm). In a 24-well polystyrene plate 1 ml of NB media containing bacterial culture at OD_600 nm_ = 0.1 (diluted from overnight grown culture) was added to each well with or without (control) 1.0 mg/ml of SJ16 extract. One glass coverslip/well was submerged and the plate was incubated in static condition at 37°C. The effect of the SJ16 extract (1.0 mg/ml) on the *P. aeruginosa* cell viability within biofilm was examined at different time points (24, 48, and 72 h) under a fluorescence microscope using FilmTracer LIVE/DEAD Biofilm Viability Kit (Invitrogen, United States) (Singh et al., 2013). The bacterial cells within the biofilm were labeled with a fluorescent dye (propidium iodide and SYTO 9), were further processed according to the manufacturer’s instructions and were visualized under an epifluorescence microscope (Axio Imager, Carl Zeiss AG, Germany).

### Scanning Electron and Atomic Force Microscopy

Scanning electron microscopy (SEM) and atomic force microscopy (AFM) were used to visualize the effect of the SJ16 extract on the topology of the biofilm developed by the *P. aeruginosa* strains. For the SEM, a previously described protocol was used (Andersson et al., 2009; Singh et al., 2013). In brief, biofilms of *P. aeruginosa* PAO1 and *P. aeruginosa* PAH were grown on glass coverslips submerged in nutrient broth in a 24-well polystyrene plate with (1.0 mg/ml) or without extract (control). The plate containing the culture and the coverslips were kept at 37°C for 24 h. After incubation, the planktonic culture was removed and coverslips were gently washed with 0.9% NaCl. The samples were treated with 2.5% glutaraldehyde for 20 min followed by 4% OsO_4_ for 30 min and dehydrated using ethanol gradient (10 to 95%) treatment for 10 min for each concentration. The dehydrated and dried biofilms were coated with gold and observed under a scanning electron microscope (SEM, LEO series VP1430, Germany). For the AFM, overnight grown cultures of *P. aeruginosa* PAO1 and *P. aeruginosa* PAH were diluted to reach an OD_600 nm_ = 0.1 in NB broth, sterile glass cover slips were submerged in 24-well polystyrene plate with (1.0 mg/ml) or without extract (control). The plate was kept for 24 h at 37°C. Following this incubation period, the biofilm that developed on the glass coverslip was rinsed gently with phosphate buffer saline (pH 7.4) and was kept under the desiccators until completely dry. Finally, the biofilm was scanned under AFM (NT-MDT, Russia) in a semi-contact mode (Oh et al., 2009; Singh et al., 2013).

### Swarming Motility Assay

The swarming motility of PAO1 and PAH were tested in the presence (1.0 mg/ml) and absence of SJ16 extract. Overnight grown culture of *P. aeruginosa* PAO1 and *P. aeruginosa* PAH were diluted to OD_600 nm_ = 1.0 and spotted on a plate containing BM2 swarming medium (62 mM PBS at pH 7, 2 mM MgSO_4_, 10 μM FeSO_4_, 0.4% glucose, 0.1% casamino acids and 0.5% agar) supplemented with 1.0 mg/ml SJ16 extract or without extract supplementation (control) (Overhage et al., 2007). The plates were incubated at 37°C for 24 h and swarming zones were observed.

### Swimming Motility Assay

The overnight grown culture of PAO1 and PAH were diluted to OD_600 nm_ = 1.0 and spotted on a plate containing tryptone broth (10 g/l tryptone, 5 g/l NaCl and 0.3% agar) supplemented with 1.0 mg/ml SJ16 extract or without extract supplementation (control) (Rashid and Kornberg, 2000). The plates were incubated at 37°C and analyzed after 24 h.

### Virulence Factor Analysis

The effect of the SJ16 bacterial extracts (1.0 mg/ml) on the production of virulence factors by reference *P. aeruginosa* strains (PAO1 and PAH) was studied by measuring the levels of pyocyanin and rhamnolipid, and by analyzing the elastase and protease activities. For pyocyanin assay, starter cultures of *P. aeruginosa* strains (PAO1 and PAH) were grown at 37 °C in an incubator shaker until OD_600 nm_ = 3.0 and diluted to OD_600 nm_ = 0.1 in PB medium (5 ml; 20 g/l peptone, 1.4 g/l MgCl_2_, and 10 g/l K_2_SO_4_). The tubes containing 5 ml PB medium (OD_600 nm_ = 0.1) supplemented with 1.0 mg/ml SJ16 extract or without extract supplementation (control). Supernatants were collected by centrifuging cultures at 10,000 × *g* for 10 min; pyocyanin was extracted in 3 ml of chloroform followed by 1 ml of 0.2 N HCl and quantified spectrophotometrically at 520 nm (Essar et al., 1990). For rhamnolipid assay, *P. aeruginosa* strains PAO1 and PAH (OD_600 nm_ = 0.1) were grown overnight at 37°C in NB medium, supplemented with 1.0 mg/ml SJ16 extract or without extract supplementation (control). The culture was centrifuged (10,000 × *g* for 10 min), the supernatant was collected and acidified to pH 2 (with HCl), and absorbance was measured at 570 nm (McClure and Schiller, 1992; Sarabhai et al., 2013).

### Elastase Assay

To estimate the elastase activity, the supernatant (750 μl) from overnight grown (with or without SJ16 extract) cultures of *P. aeruginosa* (PAO1 and PAH) were incubated with elastin congo red solution (250 μl; 5 mg/ml in 0.1 M Tris–HCl pH 8;1 mM CaCl_2_) at 37°C for 16 h. Reaction-mixtures were centrifuged (3,000 × *g* for 10 min), the supernatant was collected, and the absorbance was read at 495 nm (Bjorn et al., 1979; Zhu et al., 2002).

### Protease Assay

For the protease activity, 2% azocasein solution was prepared in 50 mM phosphate buffer saline (pH 7). The supernatant (400 μl) from overnight grown (with or without SJ16 extract) cultures of *P. aeruginosa* (PAO1 and PAH) were incubated with an equal volume of azocasein solution (2%) at 37°C for 1 h. A measure of 500 μl of 10% trichloroacetic acid was added to stop the reaction. Reaction-mixtures were centrifuged (8,000 × *g* for 5 min) to remove residual azocasein and the absorbance of the supernatant was measured at 400 nm (Adonizio et al., 2008).

### Microarray

A single colony of the reference strain *P. aeruginosa* PAO1 was inoculated in a 5 ml NB tubes, was grown until OD_600 nm_ 3.0 and was then diluted to OD_600 nm_ = 0.1. Following dilution, *P. aeruginosa* PAO1 was grown in a tube containing 5 ml starter culture (OD_600 nm_ = 0.1) with or without bacterial extracts (1.0 mg/ml) at 37°C for 24 h in a shaker incubator at 200 rpm (Singh et al., 2017). Planktonic cells were harvested by centrifuge at 12,000 × *g* for 5 min and total RNA was isolated using TRI reagent (Sigma, United States). Total RNA was analyzed on 2% agoras gel and quantified using ND-1000 spectrophotometer (Nanodrop Technologies, United States). Ten μg RNA, extracted from control and treated samples were converted to cDNA, fragmented and labeled using previously optimized method (Singh et al., 2016a). Labeled cDNAs were hybridized with genome array gene chip (Gene Chip *P. aeruginosa* PAO1 containing total 5,886 gene probes), washed, stained, and scanned (Scanner 3000 7G, Affymetrix, United States) (Singh et al., 2017). Scanned chips were processed and analyzed using the expression console and the transcriptome analysis console (Affymetrix, United States). Microarray analysis was performed in duplicate and the genes showing significant differences in fold-change expression (ANOVA *p*-value < 0.05) were considered for the study.

### Statistical Analysis

Statistical analysis was performed using GraphPad Prism software. One-way ANOVA followed by Tukey *post hoc* that was applied for the comparison of test samples and controls.

## Results

### Isolation, Molecular Identification, Phylogeny and Fatty Acid Methyl Ester Profiling

Previously, out of 56 bacterial axenic cultures which were obtained from the rhizosphere of *C. laevigatus* L., two axenic cultures (SJ01 and SJ16) showed anti-QS activity (Singh et al., 2017). The 16S rRNA gene sequence (accession no. KX130768) of isolate SJ16 showed 99% similarity with *E. indicum* with 100% query coverage; therefore, this isolate was designated as *E. indicum* SJ16. The phylogenetic analysis showed the taxonomic position of the strain SJ16 (Supplementary Figure S1). The whole-cell FAME profile of the *E. indicum* SJ16 bacterium demonstrated an abundance of the iso-C_17:0_ (17.39%), iso-C_15:0_ (14.79%) and anteiso-C_13:0_ (11.36%) fatty acids (Supplementary Figure S2).

### Identification of the Active Fraction/Compound

**FIGURE 1:**
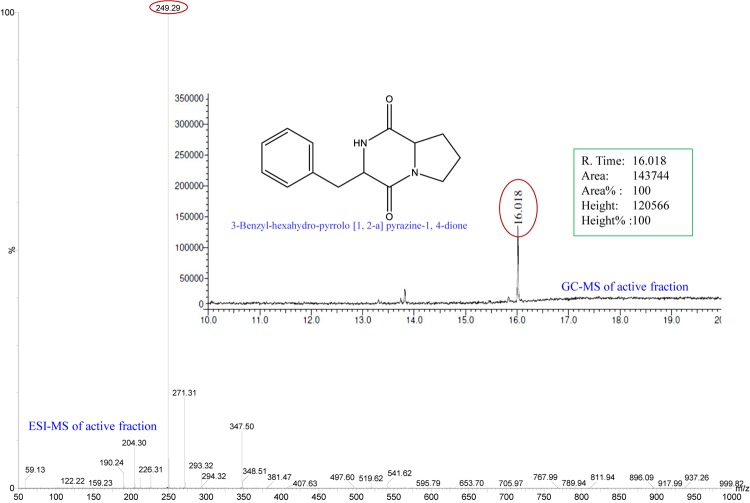
Analysis of active fraction showing QSI. GC chromatograms and ESI-MS/MS of the active fraction (C18-40) of *E. indicum* SJ16 extract showed resemblance to 3-Benzyl-hexahydro-pyrrolo[1,2-a]pyrazine-1,4-dione (redrawn by ChemBioDraw Ultra 12.0).

All fractions, collected from the various SPE cartridges (anion exchanger DAE, cation mixed Plexa PCX, polar SI, and non-polar C18) were individually screened for anti-QS activity using a biosensor plate containing the tester strain *C. violaceum* CV026. The fraction collected from the C18 cartridge (with 40% methanol) showed a maximum zone of QSI. This fraction was subjected to GC-MS analysis, and the chromatogram showed a single predominant peak at the retention time 16.01 min (Figure 1). The detected peak resembled the 3-Benzyl-hexahydro-pyrrolo[1, 2-a]pyrazine-1,4-dione (C_14_H_16_N_2_O_2_; expected molecular mass 244.28) from the GC-MS library. The active fraction was also subjected to ESI-MS analysis, which showed a dominating spectral peak at *m/z* 249.29 (Figure 1). Thus, the experimental molecular mass of the active compound was determined to be 249.29, which is corresponding to the theoretical mass of the active fraction.

### *Exiguobacterium indicum* SJ16 Showed Anti-quorum Sensing and Anti-biofilm Activities Without Inhibiting the Planktonic Growth

**FIGURE 2:**
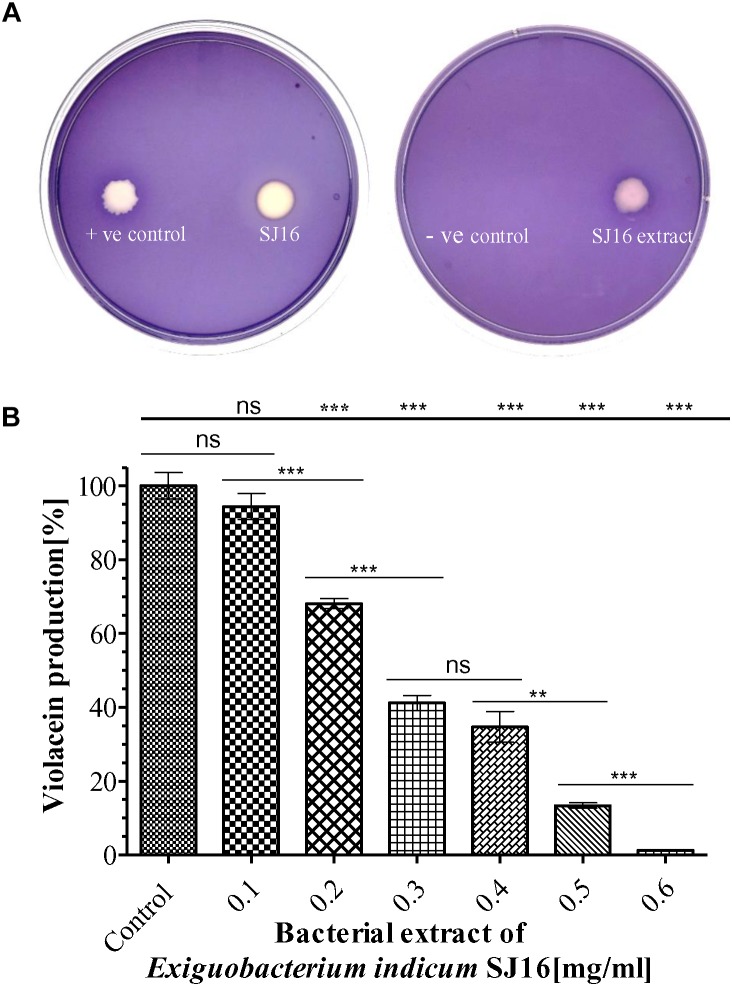
Anti-quorum sensing activity of SJ16. **(A)** The biosensor plates containing the reference strain *C. violaceum* CV026 were spotted with cinnamaldehyde (positive control), SJ16 (axenic culture), SJ16 extract, and methanol (negative control). A bright white opaque zone of inhibition showed anti-QS activity. **(B)** Different concentrations of bacterial extract (0.1–0.6 mg/ml) were used to quantify the inhibition of violacein production, an indicator of QS activity. Culture without extract was considered as a control. “^∗∗^” and “^∗∗∗^” Indicates significant differences from the control at *P* < 0.01 and *P* < 0.001, respectively.

A clear white opaque zone was observed with the axenic culture and crude extract of the *E. indicum* strain SJ16 in the biosensor plate containing the reference strain *C. violaceum* CV026, result that was comparative to the positive control with cinnamaldehyde, a well-known QS inhibitor (Figure 2). The zone of inhibition was not detected with the extraction solvent methanol that was used as a negative control. As inhibition of violacein production is an indicator of QS activity, different concentrations (0.1–0.6 mg/ml) of the SJ16 extract were used to quantify the levels of this QSI marker. Indeed, the violacein production decreased concomitantly with increasing concentrations of the extract, and about 99% inhibition was achieved with 0.6 mg/ml of the SJ16 extract (Figure 2). Furthermore, the antibacterial assay, performed by using the disc diffusion technique, confirmed that the bacterial isolate SJ16 did not halt the growth or the viability of the reference strain *C. violaceum* CV026 (Supplementary Figure S3).

**FIGURE 3:**
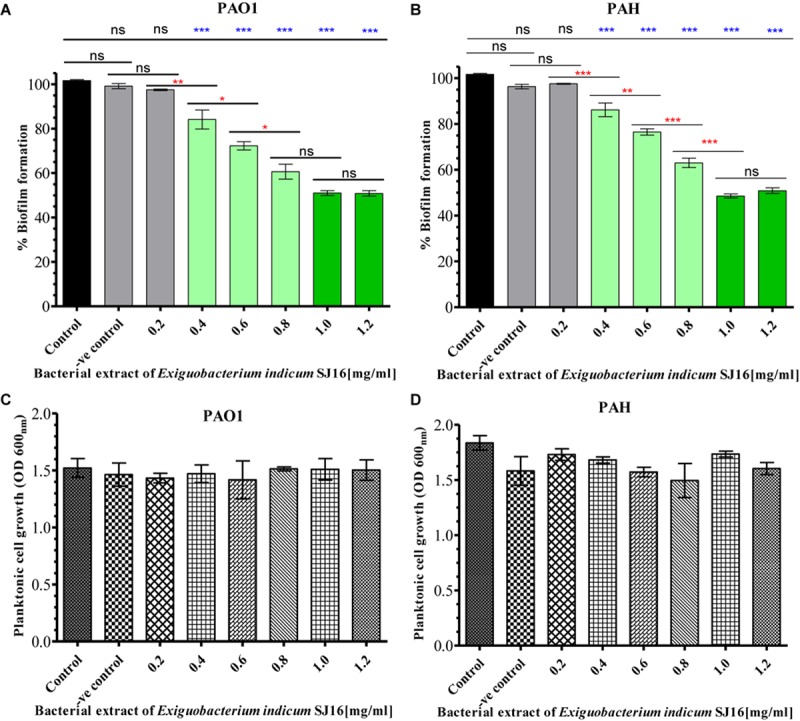
Effect of *E. indicum* SJ16 extract on biofilm formation and planktonic growth of *P. aeruginosa*. Different concentration of bacterial extracts (0.2–1.2 mg/ml) ware tested against the biofilm forming reference *P. aeruginosa* strains PAO1 **(A)** and PAH **(B)**. The same concentrations of bacterial extracts were also tested for the inhibitory effect on the planktonic growth of PAO1 **(C)** and PAH **(D)** strains. Tests without extract and with methanol were considered as control and negative control, respectively. “^∗^”, “^∗∗^” and “^∗∗∗^” Indicates significant differences from the control at *P* < 0.05, *P* < 0.01, and *P* < 0.001, respectively.

The anti-biofilm activity of *E. indicum* SJ16 extract was performed with a different concentration (0.2–1.2 mg/ml) against PAO1 and PAH strains. The biofilm formation capability of both strains significantly decreased with increasing concentrations of the extract (Figure 3). About 50% inhibition of biofilm formation was noticed with 1.0 mg/ml extract. No significant effect of this compound was observed on the planktonic growth of either PAO1 or PAH even with highest extract concentrations used (0.2–1.2 mg/ml) (Figure 3).

**FIGURE 4:**
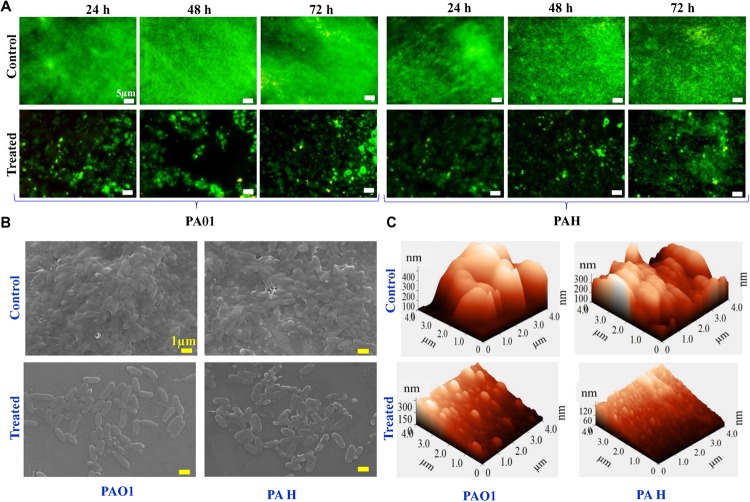
Effect of *E. indicum* SJ16 extract on cell viability, and topology of biofilms developed by *P. aeruginosa*. **(A)** The effect of the bacterial extract (1.0 mg/ml) on the viability of reference *P. aeruginosa* strains (PAO1 and PAH) in the biofilm was examined under an epifluorescence microscope at different time points (24, 48 and 72 h) and compared with control. The dead bacterial cells were labeled with propidium iodide whereas live cells were stained with SYTO 9, which produced red and green fluorescence, respectively. SEM **(B)** and AFM **(C)** images illustrate the effect of the bacterial extract (1.0 mg/ml) on biofilm formation and topology. **(B)** A well-grown biofilm along with adhering bacterial cells was observed in the control samples (normal biofilm developed by *P. aeruginosa*), whereas dispersed bacterial cells were observed in treated samples under SEM. Similarly, AFM **(C)** showed a disrupted surface topology and a height distribution profile of the biofilm developed by the reference *P. aeruginosa* strains in the presence of the bacterial extract compared to the control biofilm.

The viability of the reference strains (*P. aeruginosa* PAO1 and PAH) within the biofilm was further analyzed with the SJ16 extract at 24, 48, and 72 h. The live cells of *P. aeruginosa* were labeled with SYTO 9, whereas dead cells were stained with propidium iodide using a Live/Dead staining kit. Live and dead cells produced green and red fluorescence, respectively, when visualized under an epi-fluorescence microscope (Figure 4A). *P. aeruginosa* (PAO1 and PAH) cells, treated with SJ16 extract were not tightly attached to the biofilm surface compared to the control. There was no difference between the control and treated biofilm when considering the cell viability whithin biofilm, and insignificant numbers of dead cells were detected in the biofilm at different time points (Figure 4A). Overall, these results indicate that the SJ16 extract does not exert any toxic effect on the growth or viability of the aforementioned reference strains, even after a longer duration, or higher concentrations of the compound, while at the same time it effectively inhibits the bacterial biofilm formation (Figure 4).

### *Exiguobacterium indicum* SJ16 Extract Disrupts the Topology of the Biofilm

The effect of the SJ16 extract on the topology of the biofilm developed by the PAO1 and PAH was visualized under SEM and AFM. A biofilm along with adhering bacterial cells was developed by *P. aeruginosa* in the absence of SJ16 extract (control), whereas an immature biofilm with dispersed bacterial cells was observed when *P. aeruginosa* was grown with SJ16 extract (Figure 4B). Similarly, AFM micrographs showed changes in the topology of the biofilm developed by *P. aeruginosa* in the presence of SJ16 extract compared to normal (control) biofilm (Figure 4C). AFM showed poor biofilm adherence on the surface of the glass cover slip, while height distribution profile showed the average thickness of the biofilm developed in the presence of SJ16 extract was significantly reduced compared to the control biofilm (Figure 4C).

### *Exiguobacterium indicum* SJ16 Extract Downregulates the Motility of *P. aeruginosa*

**FIGURE 5:**
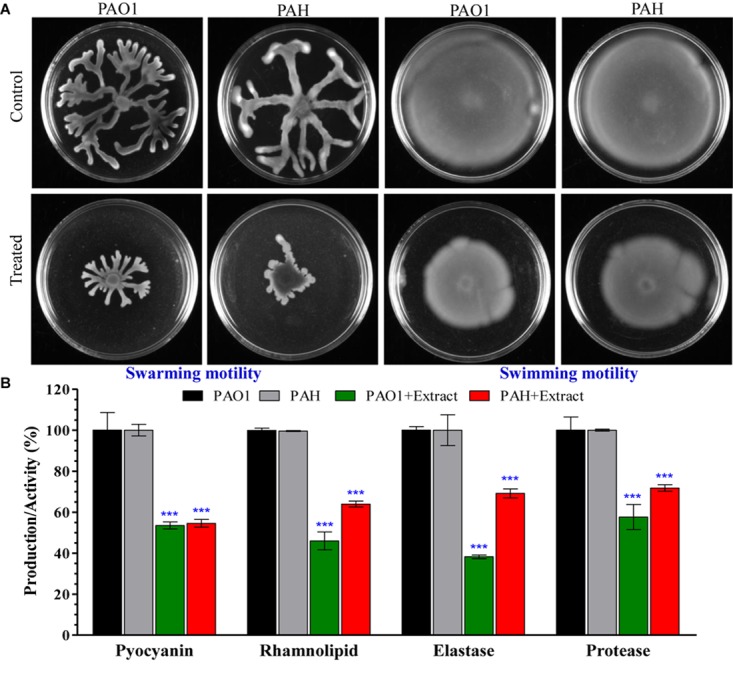
Effect of *E. indicum* SJ16 extract on cell motility, and the virulence factors of *P. aeruginosa*. **(A)** The effect of the bacterial extract on the swarming and swimming motility of reference *P. aeruginosa* strains (PAO1 and PAH). *P. aeruginosa* was spotted on a plate supplemented with 1.0 mg/ml extract or without extract supplementation (control). Plates were analyzed after incubation of 24 h at 37°C; **(B)** The effect of the bacterial extracts (SJ16; 1.0 mg/ml) was determined based on the production of virulence factors of the reference *P. aeruginosa* strains by quantifying pyocyanin and rhamnolipid, as well as by analyzing the elastase and protease activities. “^∗∗∗^” Indicates significant differences from the control at *P* < 0.001.

Bacterial motility and initial attachment of bacterial cells to surfaces are key prerequisites for biofilm formation. Therefore, the effect of the *E. indicum* SJ16 extract on the motility of reference *P. aeruginosa* strains (PAO1 and PAH) was studied. Our results show that the swarming and swimming motility of PAO1 and PAH are significantly inhibited in the presence of the SJ16 extract (Figure 5A).

### *Exiguobacterium indicum* SJ16 Extract Shows Inhibitory Effect on Virulence Activities

Virulence activities of the *P. aeruginosa* strains (PAO1 and PAH), including elastase and protease activities, as well as the production of virulence factors (pyocyanin and rhamnolipid) accelerate biofilm formation. Our data indicate that the SJ16 extract considerably reduced the production of virulence factors, with pyocyanin production decreasing about 50% in both PAO1 and PAH compared to the untreated samples (Figure 5B). Similarly, rhamnolipid production was also significantly decreased by 55% in PAO1 and 37% in PAH in the presence of the SJ16 extract compared to control (Figure 5B). Furthermore, the SJ16 extract led to a drastic inhibition of the elastase and protease activities in the above-mentioned *P. aeruginosa* strains. Specifically, elastase activity was decreased by 62% in PAO1 and by 31% PAH compared to untreated samples (Figure 5B). Similarly, protease activity was reduced by about 40% in PAO1 and by 28% in PAH in the presence of the SJ16 extract compared to control (Figure 5B). Overall, our results (Figure 3–5) strongly demonstrate that the SJ16 extract inhibits the cell-to-cell communication and thus prevents *P. aeruginosa* from developing a robust biofilm.

### Differential Expression of Quorum Sensing Regulatory Genes

**FIGURE 6:**
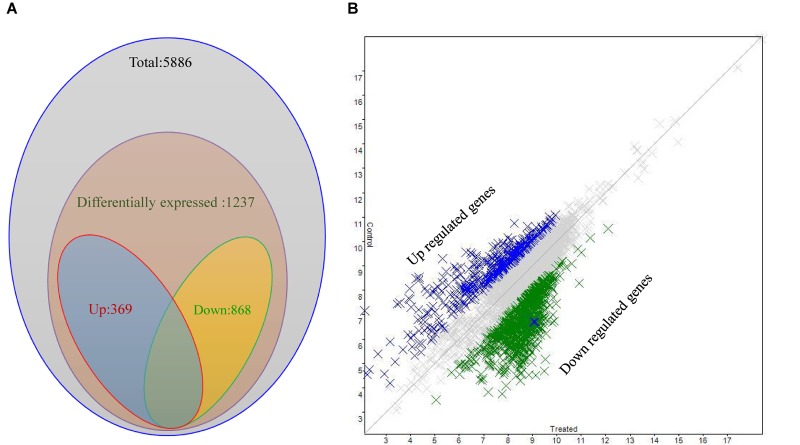
Differential expression analysis of *P. aeruginosa* PAO1 treated with SJ16 extract. **(A)** Venn diagram and **(B)** Scattered plot showing genes differentially expressed in *P. aeruginosa* PAO1 as determined by microarray.

A *P. aeruginosa* PAO1 genome array chip, containing 5,886 gene-probe sets, was used to study the effect of 3-Benzyl-hexahydro-pyrrolo[1, 2-a]pyrazine-1,4-dione on QS regulatory genes. Scattered plot analysis showed that 1,237 out of 5,886 genes (Array-Express accession E-MTAB-6829) were differentially expressed (Figure 6), with a two-fold (*p* < 0.05) up- (>2) or down-(<-2) regulation at minimum (Supplementary Table S1). More specifically, a total of 868 genes were down-regulated, whereas 369 genes were up-regulated in the presence of the compound. Among these, we identified a plethora of genes that are key players in bacterial QS network of *P. aeruginosa* and exert crucial functions in general metabolic pathways in bacteria (Table 1).

**Table 1:** Selected transcripts that are differentially expressed (up- or down- regulated) in *P. aeruginosa* PAO1, treated with the bacterial (*E. indicum* SJ16) active fraction compared to control (untreated PAO1 strain).

Transcript ID	Gene Symbol	Description	Swiss Prot	Fold Change
PA0998	*pqsC*	Homologous to beta-keto-acyl-acyl-carrier protein synthase	Q9I4X1	-11.76
PA5493_polA	*polA*	DNA polymerase I	Q9HT80	-11.3
PA1105_fliJ	*fliJ*	flagellar protein FliJ	Q9I4N0	-7.31
PA1715_pscB	*pscB*	type III export apparatus protein	Q9I320	-6.94
PA5368_pstC	*pstC*	membrane protein component of ABC phosphate transporter	Q51544	-6.62
PA3064	*pelA*	hypothetical protein	Q9HZE4	-6.18
PA1985_pqqA	*pqqA*	pyrroloquinoline quinone biosynthesis protein A	Q9ZAA0	-6.18
PA0996	*pqsA*	probable coenzyme A ligase	Q9I4X3	-5.96
PA0520_nirQ	*nirQ*	regulatory protein NirQ	Q51481	-5.6
PA1077_flgB	*flgB*	flagellar basal-body rod protein FlgB	Q9I4Q2	-5.23
PA3824_queA	*queA*	S-adenosylmethionine:trna ribosyltransferase-isomerase	Q9HXH8	-5.15
PA5501_znuB	*znuB*	permease of ABC zinc transporter ZnuB	Q9HT72	-4.97
PA2236	*pslF*	hypothetical protein	Q9I1N3	-4.76
PA1082_flgG	*flgG*	flagellar basal-body rod protein FlgG	Q9I4P7	-4.75
PA4309_pctA	*pctA*	chemotactic transducer PctA	G3XD24	-4.35
PA4224	*pchG*	pyochelin biosynthetic protein PchG	G3XCL0	-4.17
PA3061	*pelD*	hypothetical protein	Q9HZE7	-3.97
PA5450_wzt	*wzt*	ABC subunit of A-band LPS efflux transporter	P72163	-3.62
PA4225_pchF	*pchF*	pyochelin synthetase	Q9HWG4	-3.39
PA5107_blc	*blc*	outer membrane lipoprotein Blc	Q9HU76	-3.26
PA1049_pdxH	*pdxH*	pyridoxine 5’-phosphate oxidase	–	-3.21
PA1704_pcrR	*pcrR*	transcriptional regulator protein PcrR	G3XCW4	-3.01
PA2238	*pslH*	hypothetical protein	Q9I1N1	-2.74
PA3701_prfB	*prfB*	peptide chain release factor 2	–	-2.73
PA1311_phnX	*phnX*	2-phosphonoacetaldehyde hydrolase	Q9I433	-2.71
PA4229_pchC	*pchC*	pyochelin biosynthetic protein PchC	Q9HWG2	-2.38
PA2686_pfeR	*pfeR*	two-component response regulator PfeR	Q04803	-2.25
PA1720_pscG	*pscG*	type III export protein PscG	P95435	-2.13
PA0023_qor	*qor*	quinone oxidoreductase	P43903	-2.12
PA4590_pra	*pra*	protein activator	G3XDA9	-2.09
PA0409_pilH	*pilH*	twitching motility protein PilH	P43501	-2.04
PA0051	*phzH*	potential phenazine-modifying enzyme	Q9I781	-2.01
PA2425	*pvdG*	PvdG	Q9I156	2.06
PA1544_anr	*anr*	transcriptional regulator Anr	P23926	2.13
PA2399_pvdD	*pvdD*	pyoverdine synthetase D	Q9I182	2.17
PA2054_cynR	*cynR*	transcriptional regulator CynR	Q9I261	2.36
PA1783_nasA	*nasA*	nitrate transporter	Q9I2V9	2.54
PA1904	*phzF*	phzF1 and phzF2 probable phenazine biosynthesis protein	O69754	2.72
PA4034_aqpZ	*aqpZ*	aquaporin Z	Q9HWZ3	3.04
PA1723_pscJ	*pscJ*	type III export protein PscJ	Q9I314	3.39
PA1717_pscD	*pscD*	type III export protein PscD	Q9I318	3.43
PA2424	*pvdL*	PvdL	Q9I157	3.43
PA1097_fleQ	*fleQ*	transcriptional regulator FleQ	G3XCV0	4.91
PA4472_pmbA	*pmbA*	PmbA protein	Q9HVU9	5.13
PA2961_holB	*holB*	DNA polymerase III, delta prime subunit	–	7.97
PA2258_ptxR	*ptxR*	transcriptional regulator PtxR	P72131	9.11


*“-” sign means down-regulation.*

## Discussion

The genus *Exiguobacterium* is comprised of 16 species, and *E. indicum* was isolated from meltwater of the Hamta glacier (Himalayan mountain ranges) of India (Chaturvedi and Shivaji, 2006). The present study is the second report on the isolation of *E. indicum* from a rhizosphere of the coastal area after Susilowati et al. (2015), who isolated this bacteria from rice rhizosphere and reported its plant growth promotion trait. To the best of our knowledge, there is no available study, so far reporting the anti-QS activity of the genus *Exiguobacterium*. We herein for the first time demonstrate the inhibitory effect of *E. indicum* on bacterial quorum-sensing biofilm formation. In this study, the *E. indicum* SJ16 extract showed QSI activity (without any antibacterial activity; Supplementary Figure S3) against the reference strain *C. violaceum* CV026 on biosensor plates. This was also demonstrated by reduced levels of violacein in the violacein quantification assay (99% inhibition with 0.6 mg/ml SJ16 extract), which was a dose-dependent (Figure 2). A high concentration of methanolic extract (4 mg/ml) of an edible plant, *Melicope lunuankenda*, inhibited the response of *C. violaceum* CV026 to N-hexanoyl homoserine lactone (Tan et al., 2012). Similarly, Rodrigues et al. (2016) have shown that another plant methanol extract (*Eugenia uniflora*) showed inhibition of violacein production in *C. violaceum*, reaching up to 96% at the highest concentration tested. We have previously demonstrated a 98% decrease of violacein production in a dose-dependent manner with 3.7 mg/ml extract of *S. maltophilia*, as well as a 95% reduction with 0.1 mg/ml of *D. tsuruhatensis* extract, both isolated from the rhizosphere of *C. laevigatus* (Singh et al., 2013, 2017).

The treatment of biofilm-associated infections is extremely challenging, as biofilm-forming bacteria are commonly resistant to a broad spectrum of antibiotics (Høiby et al., 2010). *P. aeruginosa*, an opportunistic pathogen that causes severe infections in immunocompromised patients possesses a regulatory gene cascade that controls quorum-sensing and thus, biofilm formation and virulence factors productions. Quorum-sensing inhibition with potent QSI compounds shows promise in tackling biofilm formation in the setting of bacterial infections (Kalia, 2013; Singh et al., 2017; Quecán et al., 2018). In the field of drug discovery, extracts from natural products, especially those of microbial origin, have always been a tremendous source effective novel therapeutics (Gillespie et al., 2002; Courtois et al., 2003). These compounds have the ability to interfere with the QS system, inhibit the expression of virulence factors and prevent biofilm formation. In the present study, the *E. indicum* SJ16 extract robustly inhibits the biofilm formation of two reference *P. aeruginosa* strains (PAO1 and PAH) by modulating virulence factors (Figure 5), without affecting the planktonic growth of these bacteria (Figure 3). It is possible that these observations are the effect of the active compound within the extract that hinder the *P. aeruginosa* QS regulatory cascade (Table 1). These results are in accordance to our previous work describing the quorum quenching and anti-biofilm forming activities of the extracts from *S. maltophilia* BJ01 and *D. tsuruhatensis* SJ01 that we isolated from the rhizosphere of *C. laevigatus* (Singh et al., 2013, 2017).

In this study, the active compound that we herein describe was fractionated and identified as 3-Benzyl-hexahydro-pyrrolo[1,2-a]pyrazine-1,4-dione with a theoretical mass of 244.28 (RT-16.018) and an experimental mass of 249.28 (*m/z*) (Figure 1). Interestingly, a similar to this molecule, Pyrrolo[1,2-a]pyrazine-1,4-dione, hexahydro compound that was extracted from the newly identified species of *Streptomyces mangrovisoli* showed significant antioxidant and free-radical scavenging activities (Ser et al., 2015). Furthermore, the Hexahydropyrrolo[1,2-a]pyrazine-1,4-dione compound, isolated from the *Shewanella* sp. Lzh-2 possessed algicidal activity against several cyanobacterial and algal strains (Li et al., 2014). Moreover, the compound Pyrrolo[1,2-a]pyrazine-1,4-dione,hexahydro-3-(phenylmethyl) compound, obtained from the *Streptomyces* sp. VITPK9 showed anticandidal activity against *Candida albicans, C. krusei*, and *C. tropicalis* (Sanjenbam et al., 2014). Additionally, the Pyrrolo[2,1-c][1,4]benzodiazepine compound was demonstrated to be an effective anti-tumor drugs (Cipolla et al., 2009), while the recently described hordenine compound that was obtained from sprouting barley showed significant QSI and anti-biofilm effects against *P. aeruginosa* (Zhou et al., 2018). Taken together, compounds that are similar to the one described in our study present great potential as bioactive molecules with diverse functions including antimicrobial, anti-cancer, antioxidant and now, anti-QS activity.

As stated above, the SJ16 extract inhibits the biofilm formation of the reference *P. aeruginosa* strains PAO1 and PAH in a concentration-dependent manner without affecting their planktonic growth (Figure 3). Furthermore, epi-fluorescence microscopy confirmed the viability of *P. aeruginosa* cells within the biofilm (Figure 4A). Thus, the possibility of an inhibitory effect of the SJ16 extract on the growth of these reference strains (PAO1 and PAH) was ruled out. The SEM and AFM images suggest an alteration in the topology of the biofilm in the samples supplemented with the extract, compared to their corresponding controls, while a growing biofilm with dispersed bacterial cells was developed by *P. aeruginosa* strains in the presence of the SJ16 extract (Figure 4B,C). Disruption of biofilm architecture is a promising strategy to inhibit biofilm formation of drug-resistant *P*. *aeruginosa* strains. Importantly, in order for the bacteria to form a biofilm, they need to attach to a surface or substratum. Bacterial motility is crucial in the effort of the microbes to reach the substratum. Once attached to the surface, the bacteria spread all around via swarming and swimming type of motility, ultimately leading to biofilm formation over the surface (O’May and Tufenkji, 2011). Inhibition of the swarming and swimming motility of PAO1 and PAH was observed in the presence of the SJ16 extract (Figure 5A). It is possible that the SJ16 extract may also have the capability to block the initial attachment of *P*. *aeruginosa* by preventing the bacterial motility toward the surface. This strategy could potentially open new avenue in the effort to halt bacterial spreading and thus minimize the ability of microbes to form biofilms (Singh et al., 2017).

The *P. aeruginosa* pathogenicity depends on the ability of this microbe to produce virulence factors. Pyocyanin, rhamnolipids, elastase, and protease are the key virulence factors which are highly expressed by *P. aeruginosa* during QS, infection and biofilm formation (Driscoll et al., 2007; Sarabhai et al., 2013). Elastase and protease are involved in the early steps of host colonization by the bacterial cells, pyocyanin is crucial for the demonstration of *P. aeruginosa* virulence, while rhamnolipids facilitate the bacterial motility. The combined effect of the afore-mentioned microbial factors eventually lead to biofilm formation (O’May and Tufenkji, 2011; Sarabhai et al., 2013). Elastase and protease activities, as well as the production of pyocyanin and rhamnolipid of both *P*. *aeruginosa* strains PAO1 and PAH were significantly reduced following SJ16 extract supplementation (Figure 5B), demonstrating the ability of the extract to attenuate the *P*. *aeruginosa* virulence functions, largely regulated by the *las* and *rhl* regulatory gene cascades (De Kievit and Iglewski, 2000). Similar results were obtained with the extract of *Terminalia chebula* and *D. tsuruhatensis* (Sarabhai et al., 2013, Singh et al., 2017).

The microarray analysis revealed that at least 1,237 genes were differentially expressed following SJ16 extract supplementation, 868 out of which were down-regulated, whereas 369 were up-regulated (Supplementary Table S1 and Figure 6). These microarray results demonstrated that a plethora of genes affected by the SJ16 extract are involved in QS regulation and biofilm formation. More specifically, these genes are crucial in the control of bacterial QS, virulence, motility, cell metabolism, cell wall synthesis, and transcriptional regulation, while some others encode hypothetical proteins (Table 1). The expression profile of cells treated with the SJ16 extract containing the active 3-Benzyl-hexahydro-pyrrolo[1,2-a]pyrazine-1,4-dione compound showed that this agent downregulates genes responsible for the synthesis of the flagellar protein, the type III export apparatus protein, the flagellar basal-body rod protein, the pyochelin biosynthetic protein, the phenazine-modifying enzyme, the type III export protein, the twitching motility protein, as well as some hypothetical proteins. The extract also represses the expression of gene(s) which are involved in the biosynthesis of transcriptional regulators/ activators of QS network. These genes are controlled by QS systems and are closely associated with the pathogenicity of *P. aeruginosa* (Wagner et al., 2003). Similar results were obtained when *P. aeruginosa* PAO1 cells were treated with 1,2-benzenedicarboxylic acid, diisooctyl ester (Singh et al., 2017).

**FIGURE 7:**
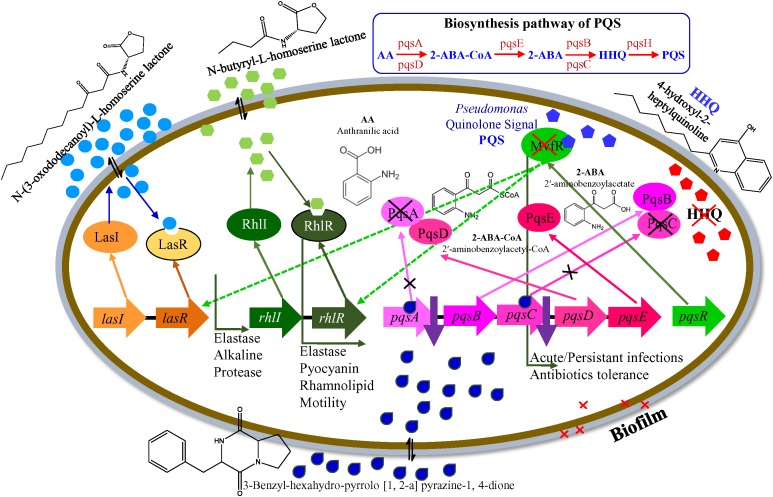
A hypothetical model for the transcriptional regulation of QSI in *P. aeruginosa* PAO1.

Based on the results that we herein report, we adopted a hypothetical model that elucidates the transcriptional regulation of *P. aeruginosa* virulence mediated by the by *E. indicum* SJ16 extract (Figure 7). We hypothesized that the identified active compound, 3-Benzyl-hexahydro-pyrrolo[1,2-a]pyrazine-1,4-dione lowers the expression of key regulatory genes including, *pqsA, pqsC, pscB, pstC, pqqA*, and *qor* (Table 1), in addition to the differential expression of other genes (Supplementary Table S1). Alteration in the transcript expression of these genes leads to lower levels of HHQ. Thus, it is possible that 3-Benzyl-hexahydro-pyrrolo[1, 2-a]pyrazine-1,4-dione modulates the function of the *pqs* transcriptional regulatory system, hence controlling the production of MvfR (also known as PqsR). Inhibition of the *MvfR* system decreases the production of QS activators and signaling molecules, thus regulating the *las* and *rhl* transcription cascades. Subsequently, *rhl* leads lower levels of pyocyanin and rhamnolipid, decreased protease and elastase activities as well as reduced bacterial motility (Déziel et al., 2005). Our results indicate that the active compound present in the SJ16 extract decreases the virulence activity through modulating the *pqs* transcription regulation system. The proposed model is a schematic representation of the regulatory mechanism, illustrated based on the available literature. However, a comprehensive study is required to confirm the precise role of the identified compound, 3-Benzyl-hexahydro-pyrrolo[1,2-a]pyrazine-1,4-dione in the QSI regulation mechanism as well as its interacting partners.

## Conclusion

The QS regulates virulence factor activities and biofilm formation, and disrupting the QS mechanism is an important strategy to inhibit pathogenicity of *P*. *aeruginosa* strains. Anti-QS compounds provides a useful tool in the effort to tackle infections caused by pathogenic bacteria. *E. indicum* SJ16, a microbe isolated from the rhizosphere of *C. laevigatus* showed promising anti-QS and anti-biofilm activities, without exhibiting any anti-bacterial properties. The 3-Benzyl-hexahydro-pyrrolo[1, 2-a]pyrazine-1,4-dione compound present in the SJ16 extract was identified as a potentially active agent inhibiting the biofilm formation of two references *P. aeruginosa* strains, PAO1 and PAH by decreasing their swimming and swarming motility and by regulating the production of virulence factors such as pyocyanin, rhamnolipid, elastase, and protease. Furthermore, it is possible that this compound controls the *pqs* QS system, thus regulating the bacterial QS mechanism as indicated by our proposed inhibitory model. Overall, our results indicate that the SJ16 extract did not have any toxic effect on the growth and viability of the reference *P. aeruginosa* strains (PAO1 and PAH), even after longer incubation periods. On the contrary, it exhibits a strong inhibitory effects on the microbial motility, on the production of virulence factors as well as on biofilm formation. Importantly, our data indicate that the SJ16 extract is able to disrupt the cell-to-cell communication (QSI) by modulating a key component of the molecular cascade regulating the *P. aeruginosa* of QS systems (*las, rhl*, and *pqs*). Therefore, the identified compound has great potential for drug development in our efforts to enrich our antimicrobial armamentarium. Further research is necessary to explore and determine its pharmaceutical applications.

## Author Contributions

All authors conceived and designed the experiments. VS performed the experiments. VS and AM analyzed the data and wrote the manuscript.

## Conflict of Interest Statement

The authors declare that the research was conducted in the absence of any commercial or financial relationships that could be construed as a potential conflict of interest.

## References

[B1] Adonizio A. Kong K. F. Mathee K. (2008). Inhibition of quorum sensing controlled virulence factor production in *Pseudomonas aeruginosa* by south florida plant extracts. *Antimicrob. Agents Chemother.* 52 198–203. 10.1128/AAC.00612-07 17938186 PMC2223872

[B2] Andersson S. Dalhammar G. Land C. Rajarao G. (2009). Characterization of extracellular polymeric substances from denitrifying organism *Comamonas denitrificans*. *Appl. Microbiol. Biotechnol.* 82 535–543. 10.1007/s00253-008-1817-3 19123000

[B3] Berendsen R. L. Pieterse C. M. J. Bakker P. A. (2012). The rhizosphere microbiome and plant health. *Trends Plant Sci.* 17 478–486. 10.1016/j.tplants.2012.04.001 22564542

[B4] Bjorn M. J. Sokol P. A. Iglewski B. H. (1979). Influence of iron on yields of extracellular products in *Pseudomonas aeruginosa* cultures. *J. Bacteriol.* 138 193–200. 108250 10.1128/jb.138.1.193-200.1979 PMC218257

[B5] Chan K. Atkinson S. Mathee K. Sam C. Chhabra S. R. Camara M. (2011). Characterization of N-acylho- moserine lactone-degrading bacteria associated with the *Zingiber officinale* (ginger) rhizosphere: co-existence of quorum quenching and quorum sensing *Acinetobacter* and *Burkholderia*. *BMC Microbiol.* 11:51. 10.1186/1471-2180-11-51 21385437 PMC3062576

[B6] Chaturvedi P. Shivaji S. (2006). Exiguobacterium indicum sp. nov., a psychrophilic bacterium from the Hamta glacier of the Himalayan mountain ranges of India. *Int. J. Syst. Evol. Microbiol.* 56 2765–2770. 10.1099/ijs.0.64508-0 17158975

[B7] Christiaen S. E. Brackman G. Nelis H. J. Coenye T. (2011). Isolation and identification of quorum quenching bacteria from environmental samples. *J. Microbiol. Methods* 87 213–219. 10.1016/j.mimet.2011.08.002 21856341

[B8] Chu W. Zhou S. Zhu W. Zhuang X. (2014). Quorum quenching bacteria *Bacillus* sp. *QSI-*1 protect zebrafish (Danio rerio) from *Aeromonas hydrophila* infection. *Sci. Rep.* 4:5446. 10.1038/srep05446 24962441 PMC4069686

[B9] Cipolla L. Araújo A. C. Airoldi C. Bini D. (2009). Pyrrolo [2, 1-c] [1, 4] benzodiazepine as a scaffold for the design and synthesis of anti-tumour drugs. *AntiCancer Agents Med. Chem.* 9 1–31. 10.2174/187152009787047743 19149479

[B10] Cirou A. Diallo S. Kurt C. Latour X. Faure D. (2007). Growth promotion of quorum-quenching bacteria in the rhizosphere of *Solanum tuberosum*. *Environ. Microbiol.* 9 1511–1522. 10.1111/j.1462-2920.2007.01270.x 17504488

[B11] Courtois S. Cappellano C. M. Ball M. Francou F. X. Normand P. Helynck G. (2003). Recombinant environmental libraries provide access to microbial diversity for drug discovery from natural products. *Appl. Environ. Microbiol.* 69 49–55. 10.1128/AEM.69.1.49-55.2003 12513976 PMC152451

[B12] D’Angelo-Picard C. Faure D. Penot I. Dessaux Y. (2005). Diversity of N-acyl homoserine lactone-producing and degrading bacteria in soil and tobacco rhizosphere. *Environ. Microbiol.* 7 1796–1808. 10.1111/j.1462-2920.2005.00886.x 16232294

[B13] De Kievit T. R. Iglewski B. H. (2000). Bacterial quorum sensing in pathogenic relationships. *Infect. Immun.* 68 4839–4849. 10.1128/IAI.68.9.4839-4849.2000 10948095 PMC101676

[B14] Déziel E. Gopalan S. Tampakaki A. P. Lépine F. Padfield K. E. Saucier M. (2005). The contribution of MvfR to *Pseudomonas aeruginosa* pathogenesis and quorum sensing circuitry regulation: multiple quorum sensing-regulated genes are modulated without affecting *lasRI, rhlRI* or the production of *N*-acyl-l-homoserine lactones. *Mol. Microbiol.* 55 998–1014. 10.1111/j.1365-2958.2004.04448.x 15686549

[B15] Driscoll J. A. Brody S. L. Kollef M. H. (2007). The epidemiology, pathogenesis, and treatment of *Pseudomonas aeruginosa* infections. *Drugs* 67 351–368. 10.2165/00003495-200767030-00003 17335295

[B16] Essar D. W. Eberly L. Hadero A. Crawford I. P. (1990). Identification and characterization of genes for a second anthranilate synthase in *Pseudomonas aeruginosa*: interchangeability of the two anthranilate synthases and evolutionary implications. *J. Bacteriol.* 172 884–900. 10.1128/JB.172.2.884-900.1990 2153661 PMC208517

[B17] Felsenstein J. (1985). Confidence limits on phylogenesis: an approach using the bootstrap. *Evolution* 39 783–791. 10.1111/j.1558-5646.1985.tb00420.x 28561359

[B18] Galloway W. R. James T. H. Bowden S. D. Welch M. Spring D. R. (2011). Quorum sensing in gram-negative bacteria: small-molecule modulation of AHL and AI-2 quorum sensing pathways. *Chem. Rev.* 111 28–67. 10.1021/cr100109t 21182299

[B19] Ganin H. Yardeni E. H. Kolodkin-Gal I. (2015). “Biofilms: maintenance, development, and disassembly of bacterial communities are determined by QS cascades,” in *Quorum Sensing vs Quorum Quenching: A Battle with No End in Sight*, ed. Kalia V. (New Delhi: Springer), 23–37. 10.1007/978-81-322-1982-8_3

[B20] Gillespie D. E. Brady S. F. Bettermann A. D. Cianciotto N. P. Liles M. R. Rondon M. R. (2002). Isolation of antibiotics turbomycin A and B fromametagenomic library of soil microbial DNA. *Appl. Environ. Microbiol.* 68 4301–4306. 10.1128/AEM.68.9.4301-4306.2002 12200279 PMC124076

[B21] Goswami N. N. Trivedi H. R. Goswami A. P. P. Patel T. K. Tripathi C. B. (2011). Antibiotic sensitivity profile of bacterial pathogens in postoperative wound infections at a tertiary care hospital in Gujarat, India. *J. Pharmacol. Pharmacother.* 2:158. 10.4103/0976-500X.83279 21897707 PMC3157123

[B22] Høiby N. Bjarnsholt T. Givskov M. Molin S. Ciofu O. (2010). Antibiotic resistance of bacterial biofilms. *Int. J. Antimicrob.* 35 322–332. 10.1016/j.ijantimicag.2009.12.011 20149602

[B23] Jha B. Singh V. K. Weiss A. Hartmann A. Schmid M. (2015). Zhihengliuella somnathii sp. nov., a halotolerant actinobacterium from the rhizosphere of a halophyte *Salicornia brachiata*. *Int. J. Syst. Evol. Microbiol.* 65 3137–3142. 10.1099/ijsem.0.000391 26297009

[B24] Kalia V. C. (2013). Quorum sensing inhibitors: an overview. *Biotechnol. Adv.* 31 224–245. 10.1016/j.biotechadv.2012.10.004 23142623

[B25] Kang B. R. Lee J. H. Ko S. J. Lee Y. H. Cha J. S. Cho B. H. (2004). Degradation of acyl-homoserine lactone molecules by *Acinetobacter* sp. strain C1010. *Can. J. Microbiol.* 50 935–941. 10.1139/w04-083 15644910

[B26] Kavita K. Singh V. K. Mishra A. Jha B. (2014). Characterisation and anti-biofilm activity of extracellular polymeric substances from *Oceanobacillus iheyensis*. *Carbohydr. Polym.* 101 29–35. 10.1016/j.carbpol.2013.08.099 24299745

[B27] Lee J. Zhang L. (2015). The hierarchy quorum sensing network in *Pseudomonas aeruginosa*. *Protein Cell* 6 26–41. 10.1007/s13238-014-0100-x 25249263 PMC4286720

[B28] Li Z. Lin S. Liu X. Tan J. Pan J. Yang H. (2014). A freshwater bacterial strain, *Shewanella* sp. *Lzh-*2, isolated from Lake Taihu and its two algicidal active substances, hexahydropyrrolo [1, 2-a] pyrazine-1, 4-dione and 2, 3-indolinedione. *Appl. Microbiol. Biotechnol.* 98 4737–4748. 10.1007/s00253-014-5602-1 24566920

[B29] McClure C. D. Schiller N. L. (1992). Effects of *Pseudomonas aeruginosa* rhamnolipids on human monocyte-derived macrophages. *J. Leukoc. Biol.* 51 97–102. 10.1002/jlb.51.2.97 1431557

[B30] Oh Y. Lee N. Jo W. Jung W. Lim J. (2009). Effects of substrates on biofilm formation observed by atomic force microscopy. *Ultramicroscopy* 109 874–880. 10.1016/j.ultramic.2009.03.042 19394143

[B31] O’May C. Tufenkji N. (2011). The swarming motility of *Pseudomonas aeruginosa* is blocked by cranberry proanthocyanidins and other tannin-containing materials. *Appl. Environ. Microbiol.* 77 3061–3067. 10.1128/AEM.02677-10 21378043 PMC3126419

[B32] O’Toole G. Kaplan H. B. Kolter R. (2000). Biofilm formation as microbial development. *Annu. Rev. Microbiol.* 54 49–79. 10.1146/annurev.micro.54.1.49 11018124

[B33] Overhage J. Lewenza S. Marr A. K. Hancock R. E. (2007). Identification of genes involved in swarming motility using a *Pseudomonas aeruginosa* PAO1 Mini-Tn5-lux mutant library. *J. Bacteriol.* 189 2164–2169. 10.1128/JB.01623-06 17158671 PMC1855721

[B34] Quecán B. X. V. Rivera M. L. C. Pinto U. M. (2018). “Bioactive phytochemicals targeting microbial activities mediated by quorum sensing,” in *the Biotechnological Applications of Quorum Sensing Inhibitors*. ed. Kalia V. (Singapore: Springer), 397–416. 10.1007/978-981-10-9026-4_19

[B35] Rashid M. H. Kornberg A. (2000). Inorganic polyphosphate is needed for swimming, swarming, and twitching motilities of *Pseudomonas aeruginosa*. *Proc. Natl. Acad. Sci. U.S.A.* 97 4885–4890. 10.1073/pnas.060030097 10758151 PMC18327

[B36] Rodrigues A. C. Zola F. G. Ávila Oliveira B. D. Sacramento N. T. B. da Silva E. R. Bertoldi M. C. (2016). Quorum quenching and microbial control through phenolic extract of *Eugenia uniflora* fruits. *J. Food Sci.* 81 M2538–M2544. 10.1111/1750-3841.13431 27603708

[B37] Saitou N. Nei M. (1987). The neighbor-joining method: a new method for reconstructing phylogenetic trees. *Mol. Biol. Evol.* 4 406–425. 10.1093/oxfordjournals.molbev.a040454 3447015

[B38] Sanjenbam P. Gopal J. V. Kannabiran K. (2014). Isolation and identification of anticandidal compound from *Streptomyces* sp. VITPK9. *Appl. Microbiol. Biotechnol.* 50 492–499. 10.1134/S0003683814050081 24674450

[B39] Sarabhai S. Sharma P. Capalash N. (2013). Ellagic acid derivatives from terminalia chebula retz. Downregulate the expression of quorum sensing genes to attenuate *Pseudomonas aeruginosa* PAO1 virulence. *PLoS One* 8:e53441. 10.1371/journal.pone.0053441 23320085 PMC3539995

[B40] Ser H. L. Palanisamy U. D. Yin W. F. Malek S. N. A. Chan K. G. Goh B. H. (2015). Presence of antioxidative agent, Pyrrolo [1, 2-a] pyrazine-1, 4-dione, hexahydro-in newly isolated *Streptomyces mangrovisoli* sp. nov. *Front. Microbiol.* 6:854. 10.3389/fmicb.2015.00854 26347733 PMC4542459

[B41] Singh V. K. Kavita K. Prabhakaran R. Jha B. (2013). Cis-9-octadecenoic acid from the rhizospheric bacterium *Stenotrophomonas maltophilia* BJ01 shows quorum quenching and anti-biofilm activities. *Biofouling* 29 855–867. 10.1080/08927014.2013.807914 23844805

[B42] Singh V. K. Mishra A. Jha B. (2016b). “Marine bacterial extracellular polymeric substances: characteristics and applications,” in *Marine Glycobiology: Principles and Applications*. ed. Kim S. (Boca Raton, FL: CRC Press), 369–377. 10.1201/9781315371399-27

[B43] Singh V. K. Mishra A. Haque I. Jha B. (2016a). A novel transcription factor-like gene *SbSDR1* acts as a molecular switch and confers salt and osmotic endurance to transgenic tobacco. *Sci. Rep.* 6:31686. 10.1038/srep31686 27550641 PMC4994045

[B44] Singh V. K. Mishra A. Jha B. (2017). Anti-quorum sensing and anti-biofilm activity of *Delftia tsuruhatensis* extract by attenuating the quorum sensing-controlled virulence factor production in *Pseudomonas aeruginosa*. *Front. Cell. Infect. Microbiol.* 7:337. 10.3389/fcimb.2017.00337 28798903 PMC5526841

[B45] Susilowati D. N. Sudiana I. M. Mubarik N. R. Suwanto A. (2015). Species and functional diversity of rhizobacteria of rice plant in the coastal soils of Indonesia. *Indones. J. Agric. Sci.* 16 39–50. 10.21082/ijas.v16n1.2015.p39-50

[B46] Tamura K. Nei M. Kumar S. (2004). Prospects for inferring very large phylogenies by using the neighbor-joining method. *Proc. Natl. Acad. Sci. U.S.A.* 101 11030–11035. 10.1073/pnas.0404206101 15258291 PMC491989

[B47] Tamura K. Stecher G. Peterson D. Filipski A. Kumar S. (2013). MEGA6: molecular evolutionary genetics analysis version 6.0. *Mol. Biol. Evol.* 30 2725–2729. 10.1093/molbev/mst197 24132122 PMC3840312

[B48] Tan L. Y. Yin W. F. Chan K. G. (2012). Silencing quorum sensing through extracts of *Melicope lunuankenda*. *Sensors* 12 4339–4351. 10.3390/s120404339 22666033 PMC3355414

[B49] Wagner V. E. Bushnell D. Passador L. Brooks A. I. Iglewski B. H. (2003). Microarray analysis of *Pseudomonas aeruginosa* quorum-sensing regulons: effects of growth phase and environment. *J. Bacteriol.* 185 2080–2095. 10.1128/JB.185.7.2080-2095.2003 12644477 PMC151498

[B50] Weisburg W. G. Barns S. M. Pelletier D. A. Lane D. J. (1991). 16S ribosomal DNA amplification for phylogenetic study. *J. Bacteriol.* 173 697–703. 10.1128/jb.173.2.697-703.1991 1987160 PMC207061

[B51] Zhou J. W. Luo H. Z. Jiang H. Jian T. K. Chen Z. Q. Jia A. Q. (2018). Hordenine, a novel quorum sensing inhibitor and anti-biofilm agent against *Pseudomonas aeruginosa*. *J. Agric. Food Chem.* 66 1620–1628. 10.1021/acs.jafc.7b05035 29353476

[B52] Zhu H. Thuruthyl S. J. Willcoxd M. D. P. (2002). Determination of quorum-sensing signal molecules and virulence factors of *Pseudomonas aeruginosa* isolates from contact lens-induced microbial keratitis. *J. Med. Microbiol.* 51 1063–1070. 10.1099/0022-1317-51-12-1063 12466404

