# Supplementary material for: 3-Benzyl-Hexahydro-Pyrrolo[1,2-a]Pyrazine-1,4-Dione Extracted From Exiguobacterium indicum Showed Anti-biofilm Activity Against Pseudomonas aeruginosa by Attenuating Quorum Sensing
Source: Front Microbiol. 2019 Jun 7;10:1269. doi: 10.3389/fmicb.2019.01269 (PMC6568026; doi:10.3389/fmicb.2019.01269)
Supplement: Supplementary file 1 [file Data_Sheet_1.PDF]

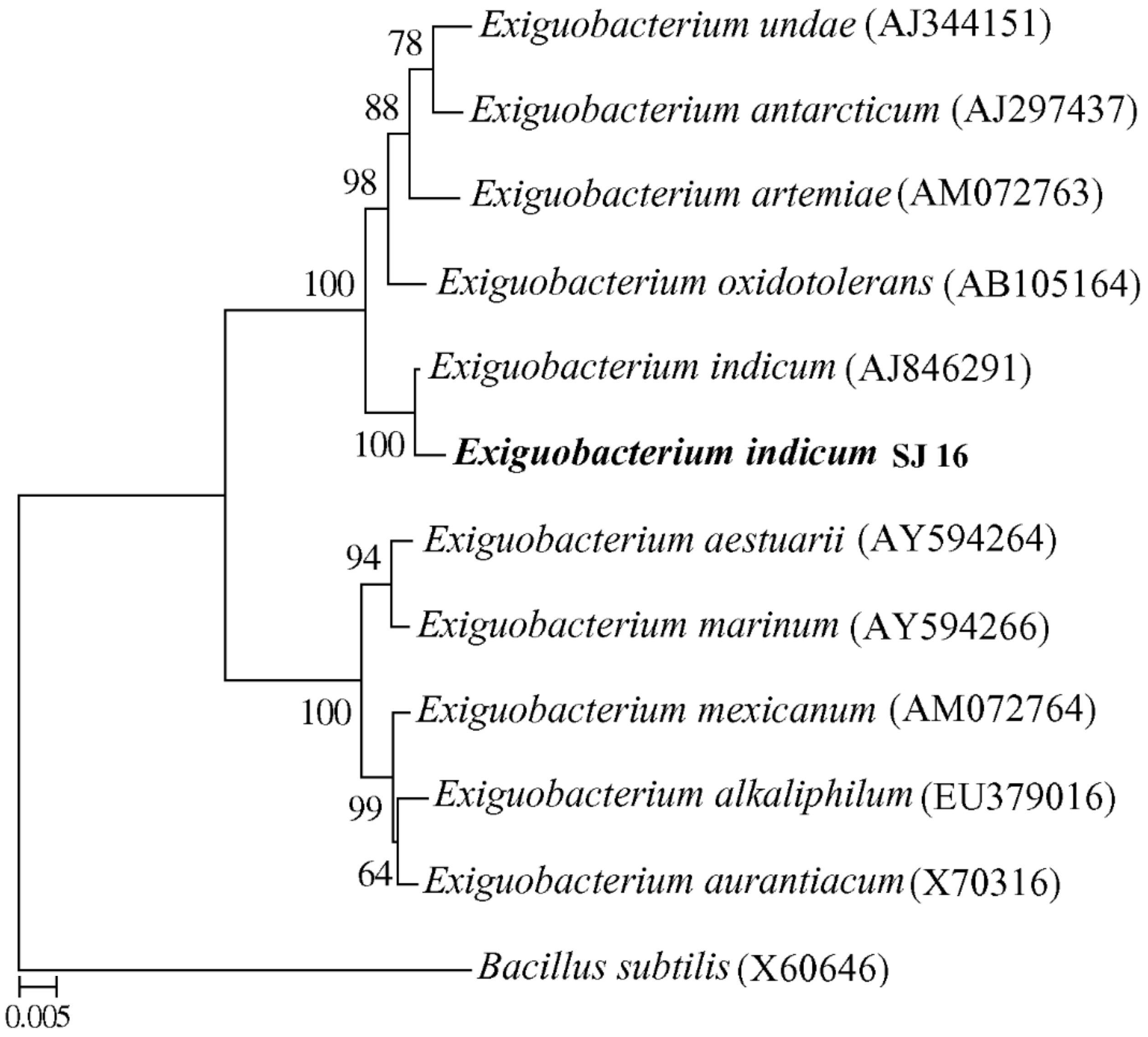

Figure S1: Phylogenetic position of *Exiguobacterium indicum* SJ16 (KX130768) with taxonomic neighbors. Numbers at nodes are percentage bootstrap values. The phylogenetic trees were computed using the maximum composite likelihood method and are in the units of the number of base substitutions per site. The complete deletion option eliminated all positions containing gaps and missing data. Phylogenetic analysis was conducted in MEGA (ver 6). Bar indicates 0.005 substitutions per nucleotide position.

RT	Response	Ar/Ht	RFact	ECL	Peak Name	Percent	Comment1	Comment2
0.7308	157072	0.005	----	6.6062		----	< min rt	
0.7395	9.892E+8	0.019	----	6.6614	SOLVENT PEAK	----	< min rt	
1.3866	1061	0.010	1.083	10.6180	11:0 iso	0.22	ECL deviates 0.000	Reference 0.000
1.4707	874	0.012	----	11.0345		----		
1.6108	7666	0.009	1.044	11.6196	12:0 iso	1.56	ECL deviates -0.001	Reference -0.003
1.7018	609	0.010	1.032	11.9991	12:0	0.12	ECL deviates -0.001	Reference -0.003
1.8723	38705	0.008	1.013	12.6250	13:0 iso	7.63	ECL deviates 0.002	Reference -0.001
1.8970	57777	0.009	1.011	12.7155	13:0 anteiso	11.36	ECL deviates 0.001	Reference -0.001
2.1624	12936	0.008	0.989	13.6287	14:0 iso	2.49	ECL deviates 0.001	Reference -0.003
2.2731	2077	0.009	0.981	13.9992	14:0	0.40	ECL deviates -0.001	Reference -0.005
2.4305	7908	0.009	0.973	14.5010	Sum In Feature 1	1.50	ECL deviates -0.002	13:0 3OH/15:1 iso H
2.4716	78334	0.009	0.970	14.6322	15:0 iso	14.79	ECL deviates 0.000	Reference -0.004
2.5007	33504	0.009	0.969	14.7247	15:0 anteiso	6.32	ECL deviates 0.000	Reference -0.005
2.5866	882	0.009	----	14.9984	15:0	----	ECL deviates -0.002	
2.7202	3825	0.009	0.960	15.4143	16:1 w7c alcohol	0.71	ECL deviates 0.000	
2.7421	3836	0.009	0.959	15.4825	16:1 iso H	0.72	ECL deviates 0.000	
2.7904	29168	0.009	0.957	15.6328	16:0 iso	5.43	ECL deviates 0.000	Reference -0.005
2.8380	6576	0.009	0.956	15.7812	16:1 w11c	1.22	ECL deviates -0.001	
2.8562	7842	0.009	0.955	15.8377	Sum In Feature 3	1.46	ECL deviates -0.002	16:1 w7c/16:1 w6c
2.8840	577	0.009	0.954	15.9243	16:1 w5c	0.11	ECL deviates -0.004	
2.9079	40373	0.008	0.953	15.9986	16:0	7.49	ECL deviates -0.001	Reference -0.007
3.0094	566	0.010	----	16.3145		----		
3.0424	19111	0.013	0.950	16.4172	17:1 iso w10c	3.53	ECL deviates 0.003	
3.0732	7771	0.009	0.949	16.5131	Sum In Feature 4	1.44	ECL deviates 0.001	17:1 anteiso B/iso I
3.0863	2914	0.009	0.949	16.5539	17:1 anteiso w9c	0.54	ECL deviates 0.002	
3.1126	94264	0.009	0.948	16.6358	17:0 iso	17.39	ECL deviates -0.001	Reference -0.007
3.1436	33100	0.009	0.948	16.7324	17:0 anteiso	6.10	ECL deviates -0.001	Reference -0.006
3.1601	680	0.009	0.947	16.7835	17:1 w9c	0.13	ECL deviates 0.001	
3.2298	3401	0.009	0.946	17.0006	17:0	0.63	ECL deviates 0.001	Reference -0.005
3.2529	724	0.010	----	17.0731		----		
3.3623	373	0.009	0.944	17.4163	17:0 10-methyl	0.07	ECL deviates 0.001	
3.3866	621	0.010	0.944	17.4923	18:1 iso H	0.11	ECL deviates 0.002	
3.4329	3978	0.010	0.943	17.6376	18:0 iso	0.73	ECL deviates 0.002	Reference -0.005
3.4802	4113	0.011	0.942	17.7859	18:1 w9c	0.75	ECL deviates -0.008	
3.4989	5411	0.010	0.942	17.8447	Sum In Feature 8	0.99	ECL deviates -0.003	18:1 w7c
3.5276	422	0.010	0.942	17.9348	18:1 w5c	0.08	ECL deviates -0.002	
3.5482	18712	0.009	0.942	17.9994	18:0	3.43	ECL deviates -0.001	Reference -0.007
3.6073	400	0.010	0.941	18.1893	17:0 iso 3OH	0.07	ECL deviates -0.004	
3.6872	463	0.010	----	18.4463		----		
3.7465	1432	0.010	0.941	18.6370	19:0 iso	0.26	ECL deviates -0.001	Reference -0.008
3.7787	741	0.011	0.940	18.7406	19:0 anteiso	0.14	ECL deviates 0.003	Reference -0.004
4.1630	427	0.010	0.940	19.9992	20:0	0.08	ECL deviates -0.001	Reference -0.008
----	7908	---	----	----	Summed Feature 1	1.50	15:1 iso H/13:0 3OH	13:0 3OH/15:1 iso H
----	7842	---	----	----	Summed Feature 3	1.46	16:1 w7c/16:1 w6c	16:1 w6c/16:1 w7c
----	7771	---	----	----	Summed Feature 4	1.44	17:1 iso I/anteiso B	17:1 anteiso B/iso I
----	5411	---	----	----	Summed Feature 8	0.99	18:1 w7c	18:1 w6c

Figure S2: Whole cell fatty acid profiling of the bacterium *E. indicum* SJ16. The whole cell fatty acid profile of strain SJ01 was performed by GC coupled with MIDI. The name of the fatty acids was assigned by corresponding fatty acids of RTSBA6 6.10 library match.

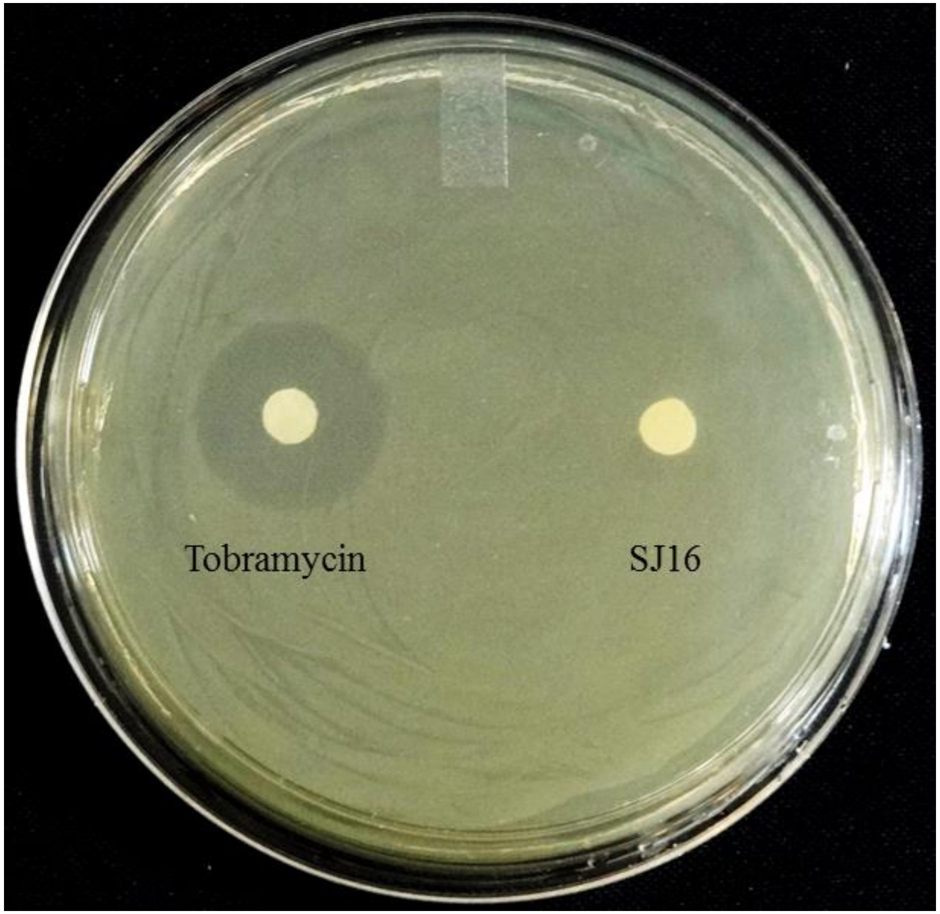

Figure S3: Antibacterial disc diffusion assay of *E. indicum* SJ16 *C. violaceum* CV026. The Mueller-Hinton agar (MHA) plate containing reference strain *C. violaceum* CV026 were tested for antibacterial activity of strain SJ16. The antibiotic tobramycin was used as a positive control.
