# Supplementary material for: 3-Benzyl-Hexahydro-Pyrrolo[1,2-a]Pyrazine-1,4-Dione Extracted From Exiguobacterium indicum Showed Anti-biofilm Activity Against Pseudomonas aeruginosa by Attenuating Quorum Sensing
Source: Front Microbiol. 2019 Jun 7;10:1269. doi: 10.3389/fmicb.2019.01269 (PMC6568026; doi:10.3389/fmicb.2019.01269)
Supplement: Supplementary file 2 [file Data_Sheet_2.PDF]

Table S1: Total transcripts that differentially expressed (up- or down- regulated) in *P. aeruginosa* PAO1, treated with bacterial (*E. indicum* SJ16).

Transcript Cluster ID	Transcript ID	Control I	Treated	Fold Chai	ANOVA p-	FDR p-value	Gene Symb	Description	Alignments	Gene Title	Pathway	SwissProt
PA2617_aat_at	PA2617_aat	9.48	6.52	7.77	0.00E+00	0.00E+00	aat	leucyl/phenyla	gb|AE004091|AE	leucyl/phenylalanyl-tRNA-prote		Q9I0M1
PA4733_acsB_at	PA4733_acsB	9.58	8.46	2.16	0.00E+00	0.00E+00	acsB	acetyl-coenzyı	gb|AE004091|AE	acetyl-coenzym	Propanoate me	Q9HV66
PA3550_algF_at	PA3550_algF	7.57	8.94	-2.57	0.00E+00	0.00E+00	algF	alginate o-ace	gb|AE004091|AE	alginate o-acety	Alginate biosyr	Q06062
PA3548_algI_at	PA3548_algI	7.98	6.97	2.01	0.00E+00	0.00E+00	algI	alginate o-ace	gb|AE004091|AE	alginate o-acety	Alginate biosyr	Q51392
PA1525_at	PA1525	7.41	9.44	-4.06	0.00E+00	0.00E+00	alkB2	alkane-1-mon	gb|AE004091|AE	alkane-1-monoc	aliphatic comp	Q6H941
PA5538_amiA_at	PA5538_amiA	4.75	7.92	-9.05	0.00E+00	0.00E+00	amiA	N-acetylmurar	gb|AE004091|AE	N-acetylmuramoyl-L-alanine an		Q9HT36
PA4522_ampD_at	PA4522_ampD	7.65	8.72	-2.1	0.00E+00	0.00E+00	ampD	beta-lactamas	gb|AE004091|AE	beta-lactamase expression regu		Q9ZGA0; G3XCW9
PA4109_ampR_at	PA4109_ampR	6.83	9.52	-6.45	0.00E+00	0.00E+00	ampR	transcriptiona	gb|AE004091|AE	transcriptional regulator AmpR		P24734
PA5287_amtB_at	PA5287_amtB	8.14	6.41	3.31	0.00E+00	0.00E+00	amtB	ammonium tra	gb|AE004091|AE	ammonium transporter AmtB		Q9HTR7
PA1544_anr_at	PA1544_anr	9.93	8.84	2.13	0.00E+00	0.00E+00	anr	transcriptiona	gb|AE004091|AE	transcriptional regulator Anr		P23926
PA2253_ansA_at	PA2253_ansA	10.06	8.94	2.17	0.00E+00	0.00E+00	ansA	L-asparaginase	gb|AE004091|AE	L-asparaginase I	Alanine and As	Q9I1L6
PA2512_antA_at	PA2512_antA	6.15	9.35	-9.16	0.00E+00	0.00E+00	antA	anthranilate d	gb|AE004091|AE	anthranilate dio	Aromatic comp	Q9I0X0
PA2513_antB_at	PA2513_antB	8.94	10.37	-2.7	0.00E+00	0.00E+00	antB	anthranilate d	gb|AE004091|AE	anthranilate dio	Aromatic comp	Q9I0W9
PA0889_aotQ_at	PA0889_aotQ	5.57	8.02	-5.48	0.00E+00	0.00E+00	aotQ	arginine/ornitl	gb|AE004091|AE	arginine/ornithine transport pr		Q9I557
PA4119_aph_at	PA4119_aph	5.42	6.73	-2.48	0.00E+00	0.00E+00	aph	aminoglycosid	gb|AE004091|AE	aminoglycoside 3'-phosphotran		Q9HWR2
PA1246_aprD_at	PA1246_aprD	6.3	8.82	-5.74	0.00E+00	0.00E+00	aprD	alkaline protea	gb|AE004091|AE	alkaline protease secretion prot		Q03024
PA4034_aqpZ_at	PA4034_aqpZ	9.13	7.52	3.04	0.00E+00	0.00E+00	aqpZ	aquaporin Z	gb|AE004091|AE	aquaporin Z		Q9HWZ3
PA5204_argA_at	PA5204_argA	6.75	8.89	-4.4	0.00E+00	0.00E+00	argA	N-acetylglutar	gb|AE004091|AE	N-acetylglutama	Arginine and pı	P22567
PA3525_argG_at	PA3525_argG	6.27	8.89	-6.15	0.00E+00	0.00E+00	argG	argininosuccin	gb|AE004091|AE	argininosuccinat	Alanine and As	Q9HY84
PA1681_aroC_at	PA1681_aroC	6.81	9.2	-5.22	0.00E+00	0.00E+00	aroC	chorismate syı	gb|AE004091|AE	chorismate synt	Phenylalanine,	Q9I344
PA0025_aroE_at	PA0025_aroE	6.38	8.02	-3.12	0.00E+00	0.00E+00	aroE	shikimate deh	gb|AE004091|AE	shikimate dehyc	Phenylalanine,	P43904
PA0899_aruB_at	PA0899_aruB	10.65	9.56	2.13	0.00E+00	0.00E+00	aruB	succinylarginir	gb|AE004091|AE	succinylarginine dihydrolase		O50175
PA0895_aruC_at	PA0895_aruC	6.37	7.75	-2.61	0.00E+00	0.00E+00	aruC	N-succinylglut	gb|AE004091|AE	N-succinylglutar	Urea cycle and	O30508
PA2001_atoB_at	PA2001_atoB	7.01	8.99	-3.93	0.00E+00	0.00E+00	atoB	acetyl-CoA ace	gb|AE004091|AE	acetyl-CoA acet	Butanoate met	Q9I2A8
PA5556_atpA_at	PA5556_atpA	7.34	9.3	-3.88	0.00E+00	0.00E+00	atpA	ATP synthase a	gb|AE004091|AE	ATP synthase al	Oxidative phos	Q9HT18
PA1008_bcp_at	PA1008_bcp	7.94	9.47	-2.88	0.00E+00	0.00E+00	bcp	bacterioferritiı	gb|AE004091|AE	bacterioferritin comigratory prc		Q9I4W5
PA0031_betC_at	PA0031_betC	5.81	7.33	-2.86	0.00E+00	0.00E+00	betC	choline sulfata	gb|AE004091|AE	choline sulfatase		Q9I799
PA4280_birA_at	PA4280_birA	7.76	8.9	-2.21	0.00E+00	0.00E+00	birA	BirA bifunctior	gb|AE004091|AE	BirA bifunctional protein		Q9HWC0
PA5107_blc_at	PA5107_blc	8.1	9.8	-3.26	0.00E+00	0.00E+00	blc	outer membra	gb|AE004091|AE	outer membrane lipoprotein Bl		Q9HU76
PA1072_braE_at	PA1072_braE	4.54	8.86	-20.03	0.00E+00	0.00E+00	braE	branched-chai	gb|AE004091|AE	branched-chain amino acid trar		P21628
PA4726_at	PA4726	7.89	9.22	-2.52	0.00E+00	0.00E+00	cbrB	two-compone	gb|AE004091|AE	two-component response regul		G3XCK7; Q9HV73
PA0584_cca_at	PA0584_cca	4.83	7.99	-8.91	0.00E+00	0.00E+00	cca	tRNA nucleoti	gb|AE004091|AE	tRNA nucleotidyl transferase		Q9I5V3
PA1477_ccmC_at	PA1477_ccmC	7.06	8.59	-2.89	0.00E+00	0.00E+00	ccmC	heme exporte	gb|AE004091|AE	heme exporter protein CcmC		Q9I3N5
PA4587_ccpR_at	PA4587_ccpR	7.64	9.03	-2.62	0.00E+00	0.00E+00	ccpR	cytochrome c5	gb|AE004091|AE	cytochrome c551 peroxidase pr		P14532
PA1801_clpP_at	PA1801_clpP	8.39	9.75	-2.55	0.00E+00	0.00E+00	clpP	ATP-depender	gb|AE004091|AE	ATP-dependent Clp protease pr		Q9I2U1
PA4529_at	PA4529	6.13	7.61	-2.77	0.00E+00	0.00E+00	coaE	dephosphocoe	gb|AE004091|AE	dephosphocoen	Pantothenate a	Q9HVP8

PA1276_cobC_at	PA1276_cobC	9.47	7.54	3.81	0.00E+00	0.00E+00	cobC	cobalamin bio	gb|AE004091|AE	cobalamin biosy	Cobalamin bios	Q9I468
PA2905_cobH_at	PA2905_cobH	6.17	7.9	-3.32	0.00E+00	0.00E+00	cobH	precorrin isom	gb|AE004091|AE	precorrin isome	Cobalamin bios	Q9HZU2
PA2948_cobM_at	PA2948_cobM	6.44	9.41	-7.82	0.00E+00	0.00E+00	cobM	precorrin-3 m	gb|AE004091|AE	precorrin-3 met	Cobalamin bios	Q9HZP9
PA1277_cobQ_at	PA1277_cobQ	7.21	9.05	-3.58	0.00E+00	0.00E+00	cobQ	cobyric acid sy	gb|AE004091|AE	cobyric acid syn	Cobalamin bios	Q9I467
PA2717_cpo_at	PA2717_cpo	7.31	8.77	-2.74	0.00E+00	0.00E+00	cpo	chloroperoxid	gb|AE004091|AE	chloroperoxidase precursor		Q9I0C5
PA3221_csaA_at	PA3221_csaA	5.8	7.5	-3.25	0.00E+00	0.00E+00	csaA	CsaA protein	gb|AE004091|AE	CsaA protein		Q9HZ19
PA0994_at	PA0994	9.46	8.1	2.56	0.00E+00	0.00E+00	cupC3	usher CupC3	gb|AE004091|AE	usher CupC3		Q9I4X5
PA2054_cynR_at	PA2054_cynR	9.08	7.84	2.36	0.00E+00	0.00E+00	cynR	transcriptiona	gb|AE004091|AE	transcriptional regulator CynR		Q9I261
PA0280_cysA_at	PA0280_cysA	7.1	8.87	-3.43	0.00E+00	0.00E+00	cysA	sulfate transp	gb|AE004091|AE	sulfate transport protein CysA		Q9I6L0
PA1838_cysI_at	PA1838_cysI	6.77	8.82	-4.16	0.00E+00	0.00E+00	cysI	sulfite reducta	gb|AE004091|AE	sulfite reductase		Q9I2Q7
PA3999_dacC_at	PA3999_dacC	9.97	8.93	2.05	0.00E+00	0.00E+00	dacC	D-ala-D-ala-ca	gb|AE004091|AE	D-ala-D-ala-carb	Peptideglycan	Q9X6V7; G3XD74
PA1124_dgt_at	PA1124_dgt	8.64	7.59	2.08	0.00E+00	0.00E+00	dgt	deoxyguanosi	gb|AE004091|AE	deoxyguanosine	Purine metabo	Q9I4L1
PA5256_dsbH_at	PA5256_dsbH	8.61	7.12	2.82	0.00E+00	0.00E+00	dsbH	disulfide bond	gb|AE004091|AE	disulfide bond formation protei		P21482
PA5321_dut_at	PA5321_dut	7.11	8.99	-3.69	0.00E+00	0.00E+00	dut	deoxyuridine 5	gb|AE004091|AE	deoxyuridine 5'-	Pyrimidine metabolism // ---	
PA2952_etfB_at	PA2952_etfB	7.08	8.77	-3.21	0.00E+00	0.00E+00	etfB	electron trans	gb|AE004091|AE	electron transfer flavoprotein b		Q9HZP6
PA4024_eutB_at	PA4024_eutB	7.09	9.09	-3.98	0.00E+00	0.00E+00	eutB	ethanolamine	gb|AE004091|AE	ethanolamine ammonia-lyase la		Q9HX03
PA1982_exaA_at	PA1982_exaA	5.14	8.53	-10.51	0.00E+00	0.00E+00	exaA	quinoprotein a	gb|AE004091|AE	quinoprotein alcohol dehydrog		Q9Z4J7
PA1983_exaB_at	PA1983_exaB	6.74	9.05	-4.96	0.00E+00	0.00E+00	exaB	cytochrome c5	gb|AE004091|AE	cytochrome c550		Q9I2C5
PA1712_exsB_at	PA1712_exsB	5.94	8.73	-6.93	0.00E+00	0.00E+00	exsB	exoenzyme S s	gb|AE004091|AE	exoenzyme S sy	Type III Secreti	P26994
PA3645_fabZ_at	PA3645_fabZ	7.7	9.1	-2.64	0.00E+00	0.00E+00	fabZ	(3R)-hydroxym	gb|AE004091|AE	(3R)-hydroxymy	Lipopolysaccha	Q9HXY7
PA3299_fadD1_at	PA3299_fadD1	6.55	8.06	-2.86	0.00E+00	0.00E+00	fadD1	long-chain-fat	gb|AE004091|AE	long-chain-fatty	Fatty acid meta	Q9HYU4
PA2008_fahA_at	PA2008_fahA	8.91	7.73	2.26	0.00E+00	0.00E+00	fahA	fumarylacetoa	gb|AE004091|AE	fumarylacetoac	Aromatic comp	Q9I2A2
PA5110_fbp_at	PA5110_fbp	9.24	7.44	3.49	0.00E+00	0.00E+00	fbp	fructose-1,6-b	gb|AE004091|AE	fructose-1,6-bis	Carbon fixation	Q9HU73
PA0555_fda_at	PA0555_fda	7.68	8.9	-2.34	0.00E+00	0.00E+00	fda	fructose-1,6-b	gb|AE004091|AE	fructose-1,6-bis	Carbon fixation	Q9I5Y1
PA4811_fdnH_at	PA4811_fdnH	6.48	8.87	-5.22	0.00E+00	0.00E+00	fdnH	nitrate-induci	gb|AE004091|AE	nitrate-inducible	Glyoxylate and	Q9HUZ8
PA3901_fecA_at	PA3901_fecA	6.92	8.55	-3.09	0.00E+00	0.00E+00	fecA	Fe(III) dicitrate	gb|AE004091|AE	Fe(III) dicitrate transport protei		Q9HXB2
PA4161_fepG_at	PA4161_fepG	7.02	8.36	-2.54	0.00E+00	0.00E+00	fepG	ferric enterob	gb|AE004091|AE	ferric enterobactin transport pr		Q9HWM0
PA3746_ffh_at	PA3746_ffh	6.96	9.16	-4.58	0.00E+00	0.00E+00	ffh	signal recognit	gb|AE004091|AE	signal recognition particle prote		Q9HXP8
PA2664_fhp_at	PA2664_fhp	5.76	7.46	-3.25	0.00E+00	0.00E+00	fhp	flavohemopro	gb|AE004091|AE	flavohemoprotein		Q9I0H4
PA1097_fleQ_at	PA1097_fleQ	9.9	7.6	4.91	0.00E+00	0.00E+00	fleQ	transcriptiona	gb|AE004091|AE	transcriptional regulator FleQ		G3XCV0
PA1077_flgB_at	PA1077_flgB	6.7	9.08	-5.23	0.00E+00	0.00E+00	flgB	flagellar basal-	gb|AE004091|AE	flagellar basal-b	Flagella assemb	Q9I4Q2
PA1082_flgG_at	PA1082_flgG	6.83	9.08	-4.75	0.00E+00	0.00E+00	flgG	flagellar basal-	gb|AE004091|AE	flagellar basal-b	Flagella assemb	Q9I4P7
PA1449_flhB_at	PA1449_flhB	9.8	8	3.48	0.00E+00	0.00E+00	flhB	flagellar biosy	gb|AE004091|AE	flagellar biosynt	Flagella assemb	Q9I3Q2
PA1105_fliJ_at	PA1105_fliJ	6.77	9.64	-7.31	0.00E+00	0.00E+00	fliJ	flagellar prote	gb|AE004091|AE	flagellar protein	Flagella assemb	Q9I4N0
PA1445_fliO_at	PA1445_fliO	5.07	3.37	3.25	0.00E+00	0.00E+00	fliO	flagellar prote	gb|AE004091|AE	flagellar protein	Flagella assemb	Q51467
PA3013_foaB_at	PA3013_foaB	7.47	6.21	2.39	0.00E+00	0.00E+00	foaB	fatty-acid oxid	gb|AE004091|AE	fatty-acid oxidat	Bile acid biosyr	Q9HZJ3
PA0350_folA_at	PA0350_folA	7.61	6.04	2.96	0.00E+00	0.00E+00	folA	dihydrofolate	gb|AE004091|AE	dihydrofolate re	Folate biosynth	Q9I6E3
PA0582_folB_at	PA0582_folB	5.54	8.17	-6.2	0.00E+00	0.00E+00	folB	dihydroneopte	gb|AE004091|AE	dihydroneopterin aldolase		Q9I5V5

PA1796_folD_at	PA1796_folD	6.8	8.59	-3.46	0.00E+00	0.00E+00	folD	5,10-methyler	gb|AE004091|AE	5,10-methylene	Glyoxylate and	Q9I2U6
PA1674_folE2_at	PA1674_folE2	7.24	8.5	-2.39	0.00E+00	0.00E+00	folE2	GTP cyclohydr	gb|AE004091|AE	GTP cyclohydrol	Folate biosynth	Q9I351
PA3439_folX_at	PA3439_folX	7.61	9.25	-3.12	0.00E+00	0.00E+00	folX	d-erythro-7,8-	gb|AE004091|AE	d-erythro-7,8-di	Folate biosynthesis // ---	
PA3397_fpr_at	PA3397_fpr	5.51	6.87	-2.57	0.00E+00	0.00E+00	fpr	ferredoxin--NA	gb|AE004091|AE	ferredoxin--NAD	Porphyrin and	Q9HYK7
PA4418_ftsI_at	PA4418_ftsI	6.92	8.72	-3.49	0.00E+00	0.00E+00	ftsI	penicillin-bind	gb|AE004091|AE	penicillin-bindin	Peptideglycan	Q51504; G3XD46
PA0373_ftsY_at	PA0373_ftsY	8.65	6.06	5.99	0.00E+00	0.00E+00	ftsY	signal recognit	gb|AE004091|AE	signal recognition particle recep		Q9I6C1
PA0928_at	PA0928	7.84	8.99	-2.21	0.00E+00	0.00E+00	gacS	sensor/respon	gb|AE004091|AE	sensor/response	Two-componen	O31138; G3XD98
PA1422_at	PA1422	6.51	8.12	-3.06	0.00E+00	0.00E+00	gbuR	GbuR	gb|AE004091|AE	GbuR	Arginine and p	Q9I3S2
PA5213_gcvP1_at	PA5213_gcvP1	10.16	8.88	2.43	0.00E+00	0.00E+00	gcvP1	glycine cleava	gb|AE004091|AE	glycine cleavage	Glycine, serine	Q9HTX7
PA5215_gcvT1_at	PA5215_gcvT1	7.76	8.9	-2.2	0.00E+00	0.00E+00	gcvT1	glycine-cleava	gb|AE004091|AE	glycine-cleavage	Glycine, serine	Q9HTX5
PA2153_glgB_at	PA2153_glgB	7.83	8.98	-2.22	0.00E+00	0.00E+00	glgB	1,4-alpha-gluc	gb|AE004091|AE	1,4-alpha-glucan	Starch and suc	Q9I1W2
PA5111_gloA3_at	PA5111_gloA3	7.46	9.97	-5.7	0.00E+00	0.00E+00	gloA3	lactoylglutathi	gb|AE004091|AE	lactoylglutathio	Pyruvate metal	Q9HU72
PA3585_glpM_at	PA3585_glpM	6.25	8.75	-5.65	0.00E+00	0.00E+00	glpM	membrane pro	gb|AE004091|AE	membrane protein GlpM		P52112
PA2444_glyA2_at	PA2444_glyA2	5.91	7.87	-3.9	0.00E+00	0.00E+00	glyA2	serine hydrox	gb|AE004091|AE	serine hydroxym	Cyanoamino ac	Q9I138
PA4602_glyA3_at	PA4602_glyA3	8.09	6.28	3.5	0.00E+00	0.00E+00	glyA3	serine hydrox	gb|AE004091|AE	serine hydroxym	Cyanoamino ac	Q9HVI7
PA2012_at	PA2012	6.24	5	2.35	0.00E+00	0.00E+00	gnyA	alpha subunit	gb|AE004091|AE	alpha subunit of	Acyclic isopren	Q9I299
PA2011_at	PA2011	9.32	7.37	3.86	0.00E+00	0.00E+00	gnyL	3-hydroxy-gan	gb|AE004091|AE	3-hydroxy-gamr	Acyclic isopren	Q9I2A0
PA5203_gshA_at	PA5203_gshA	6.39	8.38	-3.97	0.00E+00	0.00E+00	gshA	glutamate--cy	gb|AE004091|AE	glutamate--cyst	Glutamate met	Q9HTY6
PA2470_gtdA_at	PA2470_gtdA	7.05	9.38	-5.01	0.00E+00	0.00E+00	gtdA	gentisate 1,2-	gb|AE004091|AE	gentisate 1,2-di	Aromatic comp	Q9I112
PA3407_hasAp_at	PA3407_hasAp	5.87	7.22	-2.56	0.00E+00	0.00E+00	hasAp	heme acquisiti	gb|AE004091|AE	heme acquisition protein HasA		O69756; G3XD33
PA5243_hemB_at	PA5243_hemB	5.62	7.24	-3.08	0.00E+00	0.00E+00	hemB	delta-aminole	gb|AE004091|AE	delta-aminolevu	Porphyrin and	Q59643
PA5260_hemC_at	PA5260_hemC	6.44	7.79	-2.54	0.00E+00	0.00E+00	hemC	porphobilinog	gb|AE004091|AE	porphobilinogen	Porphyrin and	Q60169
PA4664_hemK_at	PA4664_hemK	8.52	7.48	2.05	0.00E+00	0.00E+00	hemK	probable meth	gb|AE004091|AE	probable methy	Porphyrin and	Q9HVC8
PA5141_hisA_at	PA5141_hisA	9.42	6.7	6.58	0.00E+00	0.00E+00	hisA	phosphoribosy	gb|AE004091|AE	phosphoribosylf	Histidine metal	Q9HU43
PA4448_hisD_at	PA4448_hisD	4.99	6.23	-2.35	0.00E+00	0.00E+00	hisD	histidinol dehy	gb|AE004091|AE	histidinol dehyd	Histidine metal	Q9HVW9
PA5142_hisH1_at	PA5142_hisH1	7.7	8.99	-2.45	0.00E+00	0.00E+00	hisH1	glutamine ami	gb|AE004091|AE	glutamine amid	Histidine metal	Q9HU42
PA5066_hisI_at	PA5066_hisI	10.32	8.88	2.71	0.00E+00	0.00E+00	hisI	phosphoribosy	gb|AE004091|AE	phosphoribosyl-	Histidine metal	Q9HUB7
PA2961_holB_at	PA2961_holB	8.89	5.9	7.97	0.00E+00	0.00E+00	holB	DNA polymera	gb|AE004091|AE	DNA polymerase III, delta prime subunit		
PA3736_hom_at	PA3736_hom	6.53	8.84	-4.94	0.00E+00	0.00E+00	hom	homoserine de	gb|AE004091|AE	homoserine del	Glycine, serine	P29365
PA0865_hpd_at	PA0865_hpd	7.13	8.18	-2.07	0.00E+00	0.00E+00	hpd	4-hydroxyphe	gb|AE004091|AE	4-hydroxyphenylpyruvate dioxy		Q9I576
PA4626_hprA_at	PA4626_hprA	9.55	6.47	8.41	0.00E+00	0.00E+00	hprA	glycerate dehy	gb|AE004091|AE	glycerate dehydrogenase		Q9HVG5
PA5105_hutC_at	PA5105_hutC	6.28	8.09	-3.53	0.00E+00	0.00E+00	hutC	histidine utiliz	gb|AE004091|AE	histidine utilizat	Histidine metal	Q9HU78
PA4694_ilvC_at	PA4694_ilvC	7.47	9.23	-3.39	0.00E+00	0.00E+00	ilvC	ketol-acid red	gb|AE004091|AE	ketol-acid reduc	Pantothenate a	Q9HVA2
PA0353_ilvD_at	PA0353_ilvD	6.85	9.36	-5.69	0.00E+00	0.00E+00	ilvD	dihydroxy-acic	gb|AE004091|AE	dihydroxy-acid	Pantothenate a	Q9I6E0
PA4696_ilvI_at	PA4696_ilvI	8.44	9.91	-2.76	0.00E+00	0.00E+00	ilvI	acetolactate s	gb|AE004091|AE	acetolactate syr	Butanoate met	Q9HVA0
PA1635_kdpC_at	PA1635_kdpC	6	8.74	-6.65	0.00E+00	0.00E+00	kdpC	potassium-tra	gb|AE004091|AE	potassium-transporting ATPase		P57686
PA3636_kdsA_at	PA3636_kdsA	5.71	8.06	-5.1	0.00E+00	0.00E+00	kdsA	2-dehydro-3-d	gb|AE004091|AE	2-dehydro-3-de	Fructose and m	Q9ZFK4
PA0927_ldhA_at	PA0927_ldhA	6.66	8.87	-4.61	0.00E+00	0.00E+00	ldhA	D-lactate dehy	gb|AE004091|AE	D-lactate dehyd	Pyruvate metal	Q9I530

PA0767_lepA_at	PA0767_lepA	8.11	9.28	-2.25	0.00E+00	0.00E+00	lepA	GTP-binding p	gb|AE004091|AE	GTP-binding protein LepA		Q9I5G8
PA3121_leuC_at	PA3121_leuC	7.87	6.26	3.06	0.00E+00	0.00E+00	leuC	3-isopropylma	gb|AE004091|AE	3-isopropylmala	Valine, leucine	Q9HZA3
PA3987_leuS_at	PA3987_leuS	8.56	7.12	2.71	0.00E+00	0.00E+00	leuS	leucyl-tRNA sy	gb|AE004091|AE	leucyl-tRNA syn	Aminoacyl-tRN	Q9HX33
PA3646_lpxD_at	PA3646_lpxD	6.85	8.18	-2.51	0.00E+00	0.00E+00	lpxD	UDP-3-O-[3-hy	gb|AE004091|AE	UDP-3-O-[3-hyd	Lipopolysaccha	Q9HXY6
PA2981_lpxK_at	PA2981_lpxK	4.96	6.08	-2.18	0.00E+00	0.00E+00	lpxK	tetraacyldisac	gb|AE004091|AE	tetraacyldisacch	Lipopolysaccharide biosynthesis // ---	
PA0936_at	PA0936	9.45	8.04	2.65	0.00E+00	0.00E+00	lpxO2	lipopolysaccha	gb|AE004091|AE	lipopolysaccharide biosynthetic		Q9I522
PA5308_lrp_at	PA5308_lrp	5.17	9.52	-20.44	0.00E+00	0.00E+00	lrp	leucine-respor	gb|AE004091|AE	leucine-responsive regulatory p		Q9HTP6
PA0212_mdcE_at	PA0212_mdcE	6.24	8.25	-4.03	0.00E+00	0.00E+00	mdcE	malonate deca	gb|AE004091|AE	malonate decarboxylase gamm		Q9I6S6
PA4901_mdlC_at	PA4901_mdlC	6.41	8.42	-4.04	0.00E+00	0.00E+00	mdlC	benzoylformat	gb|AE004091|AE	benzoylformate	Aromatic comp	Q9HUR2
PA5025_metY_at	PA5025_metY	4.91	8.48	-11.82	0.00E+00	0.00E+00	metY	homocysteine	gb|AE004091|AE	homocysteine s	Cysteine metal	Q9HUE4
PA2493_mexE_at	PA2493_mexE	8.67	7.6	2.1	0.00E+00	0.00E+00	mexE	Resistance-No	gb|AE004091|AE	Resistance-Nodulation-Cell Divi		Q9I0Y9
PA4825_mgtA_at	PA4825_mgtA	10.59	9.14	2.74	0.00E+00	0.00E+00	mgtA	Mg(2+) transp	gb|AE004091|AE	Mg(2+) transport ATPase, P-typ		Q9HUY5
PA0382_micA_at	PA0382_micA	4.57	6.9	-5.01	0.00E+00	0.00E+00	micA	DNA mismatch	gb|AE004091|AE	DNA mismatch repair protein M		Q9I6B3
PA3243_minC_at	PA3243_minC	7.5	9.1	-3.03	0.00E+00	0.00E+00	minC	cell division inl	gb|AE004091|AE	cell division inhibitor MinC		Q9HYZ7
PA3870_moaA1_at	PA3870_moaA	7.23	5.92	2.48	0.00E+00	0.00E+00	moaA1	molybdopterir	gb|AE004091|AE	molybdopterin l	Molybdopterin	Q9HXD6
PA1505_moaA2_at	PA1505_moaA	6.49	8.2	-3.26	0.00E+00	0.00E+00	moaA2	molybdopterir	gb|AE004091|AE	molybdopterin l	Molybdopterin	Q9I3K7
PA3029_moaB2_at	PA3029_moaB	6.61	8.15	-2.92	0.00E+00	0.00E+00	moaB2	molybdopterir	gb|AE004091|AE	molybdopterin l	Molybdopterin	Q9HZH7
PA3917_moaD_at	PA3917_moaD	7.56	8.81	-2.38	0.00E+00	0.00E+00	moaD	molybdopterir	gb|AE004091|AE	molybdopterin c	Molybdopterin	Q9HX96
PA3028_moeA2_at	PA3028_moeA	9.54	8.44	2.14	0.00E+00	0.00E+00	moeA2	molybdenum c	gb|AE004091|AE	molybdenum cc	Molybdopterin	Q9HZH8
PA2344_mtlZ_at	PA2344_mtlZ	9.73	8.61	2.17	0.00E+00	0.00E+00	mtlZ	fructokinase	gb|AE004091|AE	fructokinase	Fructose and m	Q9I1D4
PA2977_murB_at	PA2977_murB	5.43	8.53	-8.57	0.00E+00	0.00E+00	murB	UDP-N-acetylp	gb|AE004091|AE	UDP-N-acetylpy	Aminosugars m	Q9HZM7
PA4662_murI_at	PA4662_murI	6.97	8.67	-3.26	0.00E+00	0.00E+00	murI	glutamate rac	gb|AE004091|AE	glutamate racer	Peptideglycan l	Q9HVD0
PA5147_mutY_at	PA5147_mutY	5.32	8.41	-8.55	0.00E+00	0.00E+00	mutY	A / G specific a	gb|AE004091|AE	A / G specific adenine glycosyla		Q9HU37
PA4006_at	PA4006	7.97	5.27	6.49	0.00E+00	0.00E+00	nadD	nicotinic acid r	gb|AE004091|AE	nicotinic acid m	Nicotinate and	Q9HX21
PA1176_napF_at	PA1176_napF	4.3	8.51	-18.45	0.00E+00	0.00E+00	napF	ferredoxin pro	gb|AE004091|AE	ferredoxin prote	Nitrogen metal	Q9I4G1
PA3875_narG_at	PA3875_narG	7.83	8.99	-2.23	0.00E+00	0.00E+00	narG	respiratory nit	gb|AE004091|AE	respiratory nitra	Nitrogen metal	O54043; G3XCX1
PA3878_narX_at	PA3878_narX	6.91	9.34	-5.38	0.00E+00	0.00E+00	narX	two-compone	gb|AE004091|AE	two-component	Nitrogen metal	O54040; G3XD66
PA1783_nasA_at	PA1783_nasA	9.07	7.73	2.54	0.00E+00	0.00E+00	nasA	nitrate transp	gb|AE004091|AE	nitrate transpor	Nitrogen metal	Q9I2V9
PA3807_ndk_at	PA3807_ndk	8.86	7.77	2.13	0.00E+00	0.00E+00	ndk	nucleoside dip	gb|AE004091|AE	nucleoside diph	Purine metabo	Q59636
PA1781_nirB_at	PA1781_nirB	7.61	8.77	-2.24	0.00E+00	0.00E+00	nirB	assimilatory ni	gb|AE004091|AE	assimilatory nitr	Nitrogen metal	Q9I2W1
PA0516_nirF_at	PA0516_nirF	6.06	7.36	-2.45	0.00E+00	0.00E+00	nirF	heme d1 biosy	gb|AE004091|AE	heme d1 biosyn	Biosynthesis of	Q51480
PA0514_nirL_at	PA0514_nirL	5.46	7.83	-5.17	0.00E+00	0.00E+00	nirL	heme d1 biosy	gb|AE004091|AE	heme d1 biosyn	Biosynthesis of	P95413
PA0520_nirQ_at	PA0520_nirQ	6.78	9.27	-5.6	0.00E+00	0.00E+00	nirQ	regulatory pro	gb|AE004091|AE	regulatory prote	Denitrification	Q51481
PA2999_nqrA_at	PA2999_nqrA	7.62	8.72	-2.15	0.00E+00	0.00E+00	nqrA	Na+-transloca	gb|AE004091|AE	Na+-translocating NADH:ubiqui		Q9HZK6
PA2850_ohr_at	PA2850_ohr	9.15	7	4.42	0.00E+00	0.00E+00	ohr	organic hydro	gb|AE004091|AE	organic hydroperoxide resistan		Q9HZZ3
PA0291_oprE_at	PA0291_oprE	6.09	8.87	-6.84	0.00E+00	0.00E+00	oprE	Anaerobically-	gb|AE004091|AE	Anaerobically-induced outer m		Q51510; G3XDA5
PA1758_pabB_at	PA1758_pabB	5.23	8.2	-7.87	0.00E+00	0.00E+00	pabB	para-aminobe	gb|AE004091|AE	para-aminobenz	Folate biosynth	Q9I2Y1
PA2964_pabC_at	PA2964_pabC	8.28	9.47	-2.29	0.00E+00	0.00E+00	pabC	4-amino-4-dec	gb|AE004091|AE	4-amino-4-deoxychorismate lya		Q9HZN6

PA4397_panE_at	PA4397_panE	7.42	9.48	-4.15	0.00E+00	0.00E+00	panE	ketopantoate	gb|AE004091|AE	ketopantoate re	Pantothenate a	Q9HW09
PA2272_pbpC_at	PA2272_pbpC	6.53	8.91	-5.22	0.00E+00	0.00E+00	pbpC	penicillin-bind	gb|AE004091|AE	penicillin-binding protein 3A		Q9I1K1
PA0154_pcaG_at	PA0154_pcaG	7.71	9.35	-3.12	0.00E+00	0.00E+00	pcaG	protocatechua	gb|AE004091|AE	protocatechuate	Aromatic comp	Q9I6X8
PA4229_pchC_at	PA4229_pchC	8.54	9.79	-2.38	0.00E+00	0.00E+00	pchC	pyochelin bios	gb|AE004091|AE	pyochelin biosy	Pyochelin synth	Q9HWG2
PA4225_pchF_at	PA4225_pchF	6.14	7.91	-3.39	0.00E+00	0.00E+00	pchF	pyochelin synt	gb|AE004091|AE	pyochelin synth	Pyochelin synth	Q9HWG4
PA4224_at	PA4224	6.97	9.03	-4.17	0.00E+00	0.00E+00	pchG	pyochelin bios	gb|AE004091|AE	pyochelin biosynthetic protein I		G3XCL0; Q9HWG5
PA5292_at	PA5292	6.12	8.75	-6.21	0.00E+00	0.00E+00	pchP	phosphorylcho	gb|AE004091|AE	phosphorylcholine phosphatase		Q9HTR2
PA1165_at	PA1165	10.53	9.4	2.19	0.00E+00	0.00E+00	pcpS	PcpS	gb|AE004091|AE	PcpS	Fatty acid biosy	Q9I4H2
PA1704_pcrR_at	PA1704_pcrR	6.05	7.64	-3.01	0.00E+00	0.00E+00	pcrR	transcriptiona	gb|AE004091|AE	transcriptional r	Type III Secreti	G3XCW4
PA4309_pctA_at	PA4309_pctA	7.1	9.22	-4.35	0.00E+00	0.00E+00	pctA	chemotactic tr	gb|AE004091|AE	chemotactic tra	Chemotactic tr	G3XD24
PA1049_pdxH_at	PA1049_pdxH	6.86	8.54	-3.21	0.00E+00	0.00E+00	pdxH	pyridoxine 5'-p	gb|AE004091|AE	pyridoxine 5'-ph	Vitamin B6 metabolism // ---	
PA3064_at	PA3064	5.83	8.46	-6.18	0.00E+00	0.00E+00	pelA	hypothetical p	gb|AE004091|AE	hypothetical protein		Q9HZE4
PA3061_at	PA3061	6.56	8.55	-3.97	0.00E+00	0.00E+00	pelD	hypothetical p	gb|AE004091|AE	hypothetical protein		Q9HZE7
PA3831_pepA_at	PA3831_pepA	9.4	8.16	2.35	0.00E+00	0.00E+00	pepA	leucine amino	gb|AE004091|AE	leucine aminope	Alginate biosyr	O68822
PA2686_pfeR_at	PA2686_pfeR	7.18	8.36	-2.25	0.00E+00	0.00E+00	pfeR	two-compone	gb|AE004091|AE	two-component	Two-componer	Q04803
PA2687_pfeS_at	PA2687_pfeS	9.58	8.48	2.14	0.00E+00	0.00E+00	pfeS	two-compone	gb|AE004091|AE	two-component	Two-componer	Q04804
PA4732_pgi_at	PA4732_pgi	7.86	9.46	-3.04	0.00E+00	0.00E+00	pgi	glucose-6-pho	gb|AE004091|AE	glucose-6-phosp	Fructose and m	Q9HV67
PA5131_pgm_at	PA5131_pgm	6.92	8.49	-2.97	0.00E+00	0.00E+00	pgm	phosphoglycer	gb|AE004091|AE	phosphoglycera	Glycolysis / Glu	Q9HU53
PA3166_pheA_at	PA3166_pheA	7.35	6.27	2.1	0.00E+00	0.00E+00	pheA	chorismate mu	gb|AE004091|AE	chorismate mut	Phenylalanine,	Q9HZ67
PA2740_pheS_at	PA2740_pheS	7.88	6.56	2.49	0.00E+00	0.00E+00	pheS	phenylalanyl-t	gb|AE004091|AE	phenylalanyl-tR	Aminoacyl-tRN	Q9I0A3
PA1311_phnX_at	PA1311_phnX	6.26	7.69	-2.71	0.00E+00	0.00E+00	phnX	2-phosphonoa	gb|AE004091|AE	2-phosphonoacetaldehyde hyd		Q9I433
PA1904_s_at	PA1904	9.07	7.63	2.72	0.00E+00	0.00E+00	phzF1; phzI	probable pher	gb|AE004091|AE	probable phena	Phenazine bios	O69754
PA0051_at	PA0051	7.28	8.29	-2.01	0.00E+00	0.00E+00	phzH	potential pher	gb|AE004091|AE	potential phena	Phenazine bios	Q9I781
PA0409_pilH_at	PA0409_pilH	9.18	10.22	-2.04	0.00E+00	0.00E+00	pilH	twitching moti	gb|AE004091|AE	twitching motili	Chemotaxis //	P43501
PA5040_pilQ_at	PA5040_pilQ	6.51	9.31	-6.95	0.00E+00	0.00E+00	pilQ	Type 4 fimbria	gb|AE004091|AE	Type 4 fimbrial I	Pilin biosynthes	P34750
PA4547_pilR_at	PA4547_pilR	7.42	8.65	-2.34	0.00E+00	0.00E+00	pilR	two-compone	gb|AE004091|AE	two-component	Pilin biosynthes	Q00934
PA4553_pilX_at	PA4553_pilX	7.06	8.34	-2.44	0.00E+00	0.00E+00	pilX	type 4 fimbrial	gb|AE004091|AE	type 4 fimbrial b	Pilin biosynthes	Q9HVM9
PA2960_pilZ_at	PA2960_pilZ	6.58	9.23	-6.27	0.00E+00	0.00E+00	pilZ	type 4 fimbrial	gb|AE004091|AE	type 4 fimbrial b	Pilin biosynthes	Q51538; G3XCZ3
PA4472_pmbA_at	PA4472_pmbA	7.04	4.68	5.13	0.00E+00	0.00E+00	pmbA	PmbA protein	gb|AE004091|AE	PmbA protein		Q9HVU9
PA0196_pntB_at	PA0196_pntB	6.45	8.52	-4.21	0.00E+00	0.00E+00	pntB	pyridine nucle	gb|AE004091|AE	pyridine nucleot	Nicotinate and	Q9I6T9
PA5493_polA_at	PA5493_polA	5.15	8.64	-11.3	0.00E+00	0.00E+00	polA	DNA polymera	gb|AE004091|AE	DNA polymerase I		Q9HT80
PA1698_popN_at	PA1698_popN	8.06	6.45	3.05	0.00E+00	0.00E+00	popN	Type III secreti	gb|AE004091|AE	Type III secretio	Type III Secreti	G3XCX6
PA3610_potD_at	PA3610_potD	7.15	8.63	-2.77	0.00E+00	0.00E+00	potD	polyamine tran	gb|AE004091|AE	polyamine transport protein Po		Q9HY16
PA1770_ppsA_at	PA1770_ppsA	6.05	8.41	-5.11	0.00E+00	0.00E+00	ppsA	phosphoenolp	gb|AE004091|AE	phosphoenolpyr	Pyruvate metal	Q9I2W9
PA1985_pqqA_at	PA1985_pqqA	8.29	10.92	-6.18	0.00E+00	0.00E+00	pqqA	pyrroloquinoli	gb|AE004091|AE	pyrroloquinoline	Pyrroloquinolir	Q9ZAA0
PA0996_at	PA0996	6.89	9.46	-5.96	0.00E+00	0.00E+00	pqsA	probable coen	gb|AE004091|AE	probable coenzyme A ligase		Q9I4X3
PA0998_at	PA0998	5.24	8.8	-11.76	0.00E+00	0.00E+00	pqsC	Homologous t	gb|AE004091|AE	Homologous to beta-keto-acyl-		Q9I4X1
PA4590_pra_at	PA4590_pra	7.98	9.05	-2.09	0.00E+00	0.00E+00	pra	protein activat	gb|AE004091|AE	protein activator		G3XDA9

PA3701_prfB_at	PA3701_prfB	6.55	8	-2.73	0.00E+00	0.00E+00	prfB	peptide chain	gb|AE004091|AE	peptide chain release factor 2		
PA5050_priA_at	PA5050_priA	7.44	9.18	-3.34	0.00E+00	0.00E+00	priA	primosomal pr	gb|AE004091|AE	primosomal protein N'		
PA0956_proS_at	PA0956_proS	9.03	6.79	4.73	0.00E+00	0.00E+00	proS	prolyl-tRNA sy	gb|AE004091|AE	prolyl-tRNA synt	Aminoacyl-tRN	Q9I502
PA0796_prpB_at	PA0796_prpB	5.29	8.2	-7.49	0.00E+00	0.00E+00	prpB	carboxyphospl	gb|AE004091|AE	carboxyphosphc	Aminophosphc	Q9I5E2
PA0780_at	PA0780	6.44	8.8	-5.13	0.00E+00	0.00E+00	pruR	proline utilizat	gb|AE004091|AE	proline utilizatic	Proline utilizati	Q9I5F8
PA1715_pscB_at	PA1715_pscB	5.86	8.65	-6.94	0.00E+00	0.00E+00	pscB	type III export	gb|AE004091|AE	type III export a	Type III Secreti	Q9I320
PA1717_pscD_at	PA1717_pscD	8.41	6.63	3.43	0.00E+00	0.00E+00	pscD	type III export	gb|AE004091|AE	type III export p	Type III Secreti	Q9I318
PA1720_pscG_at	PA1720_pscG	7.8	8.89	-2.13	0.00E+00	0.00E+00	pscG	type III export	gb|AE004091|AE	type III export p	Type III Secreti	P95435
PA1723_pscJ_at	PA1723_pscJ	9.32	7.55	3.39	0.00E+00	0.00E+00	pscJ	type III export	gb|AE004091|AE	type III export p	Type III Secreti	Q9I314
PA1693_pscR_at	PA1693_pscR	5.9	8.19	-4.88	0.00E+00	0.00E+00	pscR	translocation p	gb|AE004091|AE	translocation pr	Type III Secreti	Q9I334
PA4957_psd_at	PA4957_psd	4.92	7.38	-5.51	0.00E+00	0.00E+00	psd	phosphatidylse	gb|AE004091|AE	phosphatidylser	Glycerolipid me	Q9HUK8
PA2232_at	PA2232	6.98	8.07	-2.12	0.00E+00	0.00E+00	pslB	probable phos	gb|AE004091|AE	probable phosp	Fructose and m	Q9I1N7
PA2236_at	PA2236	6.6	8.85	-4.76	0.00E+00	0.00E+00	pslF	hypothetical p	gb|AE004091|AE	hypothetical protein		Q9I1N3
PA2238_at	PA2238	6.74	8.2	-2.74	0.00E+00	0.00E+00	pslH	hypothetical p	gb|AE004091|AE	hypothetical protein		Q9I1N1
PA5368_pstC_at	PA5368_pstC	6.48	9.21	-6.62	0.00E+00	0.00E+00	pstC	membrane prc	gb|AE004091|AE	membrane protein component		Q51544; G3XD02
PA0835_pta_at	PA0835_pta	6.71	8.74	-4.08	0.00E+00	0.00E+00	pta	phosphate ace	gb|AE004091|AE	phosphate acet	Pyruvate metal	Q9I5A5
PA2258_ptxR_at	PA2258_ptxR	8.54	5.35	9.11	0.00E+00	0.00E+00	ptxR	transcriptiona	gb|AE004091|AE	transcriptional regulator PtxR		P72131
PA4855_purD_at	PA4855_purD	6.4	8.85	-5.47	0.00E+00	0.00E+00	purD	phosphoribosy	gb|AE004091|AE	phosphoribosyla	Purine metabo	Q9HUV8
PA0783_putP_at	PA0783_putP	7.56	9.09	-2.9	0.00E+00	0.00E+00	putP	sodium/prolin	gb|AE004091|AE	sodium/proline symporter PutP		Q9I5F5
PA2254_pvcA_at	PA2254_pvcA	6.34	8.29	-3.88	0.00E+00	0.00E+00	pvcA	pyoverdine bic	gb|AE004091|AE	pyoverdine bios	Pyoverdine syn	Q9I1L5
PA2399_pvdD_at	PA2399_pvdD	9.18	8.06	2.17	0.00E+00	0.00E+00	pvdD	pyoverdine syı	gb|AE004091|AE	pyoverdine synthetase D		Q9I182
PA2425_at	PA2425	9.15	8.11	2.06	0.00E+00	0.00E+00	pvdG	PvdG	gb|AE004091|AE	PvdG	Pyoverdine syn	Q9I156
PA2424_at	PA2424	8.05	6.27	3.43	0.00E+00	0.00E+00	pvdL	PvdL	gb|AE004091|AE	PvdL	Pyoverdine syn	Q9I157
PA5331_pyrE_at	PA5331_pyrE	6.37	8.11	-3.34	0.00E+00	0.00E+00	pyrE	orotate phosp	gb|AE004091|AE	orotate phosphc	Pyrimidine met	P50587
PA5541_at	PA5541	9.71	8.68	2.04	0.00E+00	0.00E+00	pyrQ	dihydroorotas	gb|AE004091|AE	dihydroorotase	Pyrimidine met	Q9HT33
PA0023_qor_at	PA0023_qor	8.29	9.37	-2.12	0.00E+00	0.00E+00	qor	quinone oxido	gb|AE004091|AE	quinone oxidoreductase		P43903
PA3824_queA_at	PA3824_queA	6.05	8.42	-5.15	0.00E+00	0.00E+00	queA	S-adenosylme	gb|AE004091|AE	S-adenosylmethionine:trna ribc		Q9HXH8
PA4743_rbfA_at	PA4743_rbfA	7.38	5.41	3.9	0.00E+00	0.00E+00	rbfA	ribosome-binc	gb|AE004091|AE	ribosome-binding factor A		Q9HV56
PA1947_rbsA_at	PA1947_rbsA	8.54	6.56	3.96	0.00E+00	0.00E+00	rbsA	ribose transpo	gb|AE004091|AE	ribose transport protein RbsA		Q9I2F7
PA4285_recC_at	PA4285_recC	7.64	8.83	-2.28	0.00E+00	0.00E+00	recC	exodeoxyribor	gb|AE004091|AE	exodeoxyribonuclease V gamm		Q9HWB5
PA1534_recR_at	PA1534_recR	5.47	8.35	-7.37	0.00E+00	0.00E+00	recR	recombinatior	gb|AE004091|AE	recombination protein RecR		Q9I3H9
PA5296_rep_at	PA5296_rep	7.63	8.99	-2.57	0.00E+00	0.00E+00	rep	ATP-depender	gb|AE004091|AE	ATP-dependent DNA helicase R		Q9HTQ8
PA3337_rfaD_at	PA3337_rfaD	5.91	8.52	-6.08	0.00E+00	0.00E+00	rfaD	ADP-L-glycero-	gb|AE004091|AE	ADP-L-glycero-D	Fructose and m	Q9HYQ8
PA3477_rhlR_at	PA3477_rhlR	8.25	7.14	2.15	0.00E+00	0.00E+00	rhlR	transcriptiona	gb|AE004091|AE	transcriptional regulator RhlR		P54292
PA4055_ribC_at	PA4055_ribC	5.97	7.98	-4.03	0.00E+00	0.00E+00	ribC	riboflavin synt	gb|AE004091|AE	riboflavin syntha	Riboflavin meta	Q9HWX3
PA4561_ribF_at	PA4561_ribF	8.23	9.52	-2.45	0.00E+00	0.00E+00	ribF	riboflavin kina	gb|AE004091|AE	riboflavin kinase	Riboflavin meta	Q9HVM3
PA5197_rimK_at	PA5197_rimK	9.95	7.8	4.45	0.00E+00	0.00E+00	rimK	ribosomal prot	gb|AE004091|AE	ribosomal protein S6 modificati		Q9HTZ2
PA5454_rmd_at	PA5454_rmd	7.5	8.89	-2.62	0.00E+00	0.00E+00	rmd	oxidoreductas	gb|AE004091|AE	oxidoreductase	Lipopolysaccha	Q9HTB6

PA5161_rmlB_at	PA5161_rmlB	7.04	8.99	-3.85	0.00E+00	0.00E+00	rmlB	dTDP-D-glucos	gb|AE004091|AE	dTDP-D-glucose	Lipopolysaccha	Q9HU24
PA1815_rnhA_at	PA1815_rnhA	7.49	9.02	-2.9	0.00E+00	0.00E+00	rnhA	ribonuclease H	gb|AE004091|AE	ribonuclease H		Q9I2S9
PA4937_rnr_at	PA4937_rnr	10.81	9.69	2.17	0.00E+00	0.00E+00	rnr	exoribonuclea	gb|AE004091|AE	exoribonuclease RNase R		Q9HUM7
PA4002_rodA_at	PA4002_rodA	6.81	9.25	-5.41	0.00E+00	0.00E+00	rodA	rod shape-det	gb|AE004091|AE	rod shape-determining protein		Q9X6V4; G3XD88
PA4433_rplM_at	PA4433_rplM	7.41	8.59	-2.26	0.00E+00	0.00E+00	rplM	50S ribosomal	gb|AE004091|AE	50S ribosomal protein L13		Q9HVY2
PA3742_rplS_at	PA3742_rplS	6.53	8.75	-4.66	0.00E+00	0.00E+00	rplS	50S ribosomal	gb|AE004091|AE	50S ribosomal protein L19		Q9HXQ2
PA2741_rplT_at	PA2741_rplT	7.77	6.63	2.21	0.00E+00	0.00E+00	rplT	50S ribosomal	gb|AE004091|AE	50S ribosomal protein L20		Q9I0A2
PA4261_rplW_at	PA4261_rplW	10.26	9.14	2.17	0.00E+00	0.00E+00	rplW	50S ribosomal	gb|AE004091|AE	50S ribosomal protein L23		Q9HWD7
PA4255_rpmC_at	PA4255_rpmC	5.47	7.23	-3.38	0.00E+00	0.00E+00	rpmC	50S ribosomal	gb|AE004091|AE	50S ribosomal protein L29		Q9HWE3
PA5049_rpmE_at	PA5049_rpmE	8.38	9.43	-2.07	0.00E+00	0.00E+00	rpmE	50S ribosomal	gb|AE004091|AE	50S ribosomal protein L31		Q9HUD0
PA4270_rpoB_at	PA4270_rpoB	6.53	9.23	-6.49	0.00E+00	0.00E+00	rpoB	DNA-directed	gb|AE004091|AE	DNA-directed RNA polymerase		Q51561
PA3656_rpsB_at	PA3656_rpsB	6.48	8.58	-4.27	0.00E+00	0.00E+00	rpsB	30S ribosomal	gb|AE004091|AE	30S ribosomal protein S2		O82850
PA4257_rpsC_at	PA4257_rpsC	7.72	8.9	-2.27	0.00E+00	0.00E+00	rpsC	30S ribosomal	gb|AE004091|AE	30S ribosomal protein S3		Q9HWE1
PA4239_rpsD_at	PA4239_rpsD	7.92	4.95	7.85	0.00E+00	0.00E+00	rpsD	30S ribosomal	gb|AE004091|AE	30S ribosomal protein S4		O52759
PA4264_rpsJ_at	PA4264_rpsJ	9.09	10.61	-2.87	0.00E+00	0.00E+00	rpsJ	30S ribosomal	gb|AE004091|AE	30S ribosomal protein S10		Q9HWD4
PA3745_rpsP_at	PA3745_rpsP	5.96	7.58	-3.06	0.00E+00	0.00E+00	rpsP	30S ribosomal	gb|AE004091|AE	30S ribosomal protein S16		Q9HXP9
PA4563_rpsT_at	PA4563_rpsT	9.55	11	-2.73	0.00E+00	0.00E+00	rpsT	30S ribosomal	gb|AE004091|AE	30S ribosomal p	Urea cycle and	Q9HVM1
PA4316_sbcB_at	PA4316_sbcB	4.95	8.5	-11.74	0.00E+00	0.00E+00	sbcB	exodeoxyribor	gb|AE004091|AE	exodeoxyribonuclease I		Q9HW85
PA1583_sdhA_at	PA1583_sdhA	5.47	7.53	-4.16	0.00E+00	0.00E+00	sdhA	succinate deh	gb|AE004091|AE	succinate dehydrogenase (A sul		Q9I3D5
PA1642_selD_at	PA1642_selD	8.47	7.15	2.5	0.00E+00	0.00E+00	selD	selenophosph	gb|AE004091|AE	selenophosphat	Selenoamino a	Q9I383
PA4768_smpB_at	PA4768_smpB	6.07	7.79	-3.29	0.00E+00	0.00E+00	smpB	SmpB protein	gb|AE004091|AE	SmpB protein		Q9HV40
PA4468_sodM_at	PA4468_sodM	6.71	8.8	-4.26	0.00E+00	0.00E+00	sodM	superoxide dis	gb|AE004091|AE	superoxide dismutase		P53652
PA5417_soxD_at	PA5417_soxD	7.52	8.97	-2.73	0.00E+00	0.00E+00	soxD	sarcosine oxid	gb|AE004091|AE	sarcosine oxidase delta subunit		Q9HTE7
PA5562_spoOJ_at	PA5562_spoOJ	9.56	6.29	9.61	0.00E+00	0.00E+00	spoOJ	chromosome	gb|AE004091|AE	chromosome partitioning prote		Q9HT12
PA0297_at	PA0297	9.24	8.13	2.16	0.00E+00	0.00E+00	spuA	probable gluta	gb|AE004091|AE	probable glutamine amidotrans		Q9I6J4
PA0300_potF2_at	PA0300_potF2	7.3	9.26	-3.89	0.00E+00	0.00E+00	spuD	polyamine tra	gb|AE004091|AE	polyamine transport protein		Q9I6J1
PA0301_potF3_at	PA0301_potF3	7.59	9.4	-3.51	0.00E+00	0.00E+00	spuE	polyamine tra	gb|AE004091|AE	polyamine transport protein		Q9I6J0
PA0302_potG_at	PA0302_potG	6.44	8.26	-3.53	0.00E+00	0.00E+00	spuF	polyamine tra	gb|AE004091|AE	polyamine transport protein Po		Q9I6I9
PA1671_stk1_at	PA1671_stk1	6.77	8.06	-2.45	0.00E+00	0.00E+00	stk1	serine-threoni	gb|AE004091|AE	serine-threonine kinase Stk1		Q9I354
PA1670_stp1_at	PA1670_stp1	10.67	9.63	2.05	0.00E+00	0.00E+00	stp1	serine/threoni	gb|AE004091|AE	serine/threonine phosphoprote		Q9I355
PA1858_str_at	PA1858_str	9.67	8.63	2.06	0.00E+00	0.00E+00	str	streptomycin 3	gb|AE004091|AE	streptomycin 3''-phosphotransf		Q9I2N7
PA0594_surA_at	PA0594_surA	7.57	9.16	-3	0.00E+00	0.00E+00	surA	peptidyl-proly	gb|AE004091|AE	peptidyl-prolyl cis-trans isomer		Q9I5U3
PA5070_tatC_at	PA5070_tatC	6.43	7.89	-2.76	0.00E+00	0.00E+00	tatC	transport prot	gb|AE004091|AE	transport protein TatC		Q9HUB3
PA0651_trpC_at	PA0651_trpC	6.7	8.69	-3.98	0.00E+00	0.00E+00	trpC	indole-3-glyce	gb|AE004091|AE	indole-3-glycerc	Phenylalanine,	P20577
PA4439_trpS_at	PA4439_trpS	9.69	8.01	3.21	0.00E+00	0.00E+00	trpS	tryptophanyl-t	gb|AE004091|AE	tryptophanyl-tR	Aminoacyl-tRN	Q9HVX6
PA3114_truA_at	PA3114_truA	7.54	9.03	-2.81	0.00E+00	0.00E+00	truA	tRNA-pseudou	gb|AE004091|AE	tRNA-pseudouri	Pyrimidine met	O87016
PA5358_ubiA_at	PA5358_ubiA	7.95	9.41	-2.75	0.00E+00	0.00E+00	ubiA	4-hydroxyben:	gb|AE004091|AE	4-hydroxybenzc	Ubiquinone bic	Q9HTK0
PA5063_ubiE_at	PA5063_ubiE	10.05	9.03	2.02	0.00E+00	0.00E+00	ubiE	ubiquinone bi	gb|AE004091|AE	ubiquinone bios	Ubiquinone bic	Q9HUC0

PA3171_ubiG_at	PA3171_ubiG	7.51	8.6	-2.14	0.00E+00	0.00E+00	ubiG	3-demethylub	gb|AE004091|AE	3-demethylubiq	Ubiquinone bic	Q9HZ63
PA5223_ubiH_at	PA5223_ubiH	7.54	8.56	-2.03	0.00E+00	0.00E+00	ubiH	ubiH protein	gb|AE004091|AE	ubiH protein	Ubiquinone bic	Q9HTW7
PA4647_uraA_at	PA4647_uraA	8.9	7.86	2.05	0.00E+00	0.00E+00	uraA	uracil permeas	gb|AE004091|AE	uracil permease		Q9HVE5
PA4867_ureB_at	PA4867_ureB	4.61	8.54	-15.24	0.00E+00	0.00E+00	ureB	urease beta su	gb|AE004091|AE	urease beta subunit		Q9HUU6
PA4904_vanA_at	PA4904_vanA	7.19	8.73	-2.9	0.00E+00	0.00E+00	vanA	vanillate O-der	gb|AE004091|AE	vanillate O-dem	Aromatic comp	Q9HUQ9
PA4905_vanB_at	PA4905_vanB	7.19	8.29	-2.14	0.00E+00	0.00E+00	vanB	vanillate O-der	gb|AE004091|AE	vanillate O-dem	Aromatic comp	Q9HUQ8
PA3155_wbpE_at	PA3155_wbpE	7.78	8.99	-2.32	0.00E+00	0.00E+00	wbpE	probable amin	gb|AE004091|AE	probable amino	Lipopolysaccha	Q9HZ76
PA3148_wbpI_at	PA3148_wbpI	5.77	8.32	-5.85	0.00E+00	0.00E+00	wbpI	probable UDP-	gb|AE004091|AE	probable UDP-N	Lipopolysaccha	G3XD61
PA3706_at	PA3706	5.45	7.3	-3.61	0.00E+00	0.00E+00	wspC	probable prote	gb|AE004091|AE	probable protei	Chemosensory	Q9HXT5
PA3704_at	PA3704	6.71	8.57	-3.64	0.00E+00	0.00E+00	wspE	probable chen	gb|AE004091|AE	probable chemo	Chemosensory	Q9HXT7
PA5450_wzt_at	PA5450_wzt	6.26	8.12	-3.62	0.00E+00	0.00E+00	wzt	ABC subunit o	gb|AE004091|AE	ABC subunit of /	Lipopolysaccha	P72163; G3XD58
PA3105_xcpQ_at	PA3105_xcpQ	7.97	9.33	-2.56	0.00E+00	0.00E+00	xcpQ	general secret	gb|AE004091|AE	general secretion pathway prot		P35818
PA2518_xylX_at	PA2518_xylX	6.37	8.61	-4.74	0.00E+00	0.00E+00	xylX	toluate 1,2-dic	gb|AE004091|AE	toluate 1,2-diox	Aromatic comp	Q9I0W4
PA2517_xylY_at	PA2517_xylY	7.85	9.03	-2.25	0.00E+00	0.00E+00	xylY	toluate 1,2-dic	gb|AE004091|AE	toluate 1,2-diox	Aromatic comp	Q9I0W5
PA5193_yrfI_at	PA5193_yrfI	7.71	9.25	-2.89	0.00E+00	0.00E+00	yrfI	heat shock prc	gb|AE004091|AE	heat shock protein HSP33		Q9HTZ6
PA5501_znuB_at	PA5501_znuB	6.78	9.09	-4.97	0.00E+00	0.00E+00	znuB	permease of A	gb|AE004091|AE	permease of ABC zinc transport		Q9HT72
PA3905_at	PA3905	6.47	4.7	3.41	0.00E+00	0.00E+00			gb|AE004091|AE004091:4374329-4374857 (+) /			Q9HXA8
PA3909_at	PA3909	7.81	8.85	-2.05	0.00E+00	0.00E+00			gb|AE004091|AE004091:4377310-4379650 (-) //			Q9HXA4
PA3939_at	PA3939	7.65	8.81	-2.24	0.00E+00	0.00E+00			gb|AE004091|AE004091:4417013-4417934 (-) //			Q9HX77
PA3908_at	PA3908	5.08	6.68	-3.04	0.00E+00	0.00E+00			gb|AE004091|AE004091:4376011-4376731 (+) /			Q9HXA5
PA3936_at	PA3936	7.22	8.88	-3.17	0.00E+00	0.00E+00			gb|AE004091|AE004091:4414131-4414950 (-) //			Q9HX80
PA4118_at	PA4118	8.25	7.08	2.24	0.00E+00	0.00E+00			gb|AE004091|AE004091:4606884-4607454 (+) /			G3XD59
Pae_AF241171cds12	Pae_AF241171	6.03	4.02	4.03	0.00E+00	0.00E+00						
PA3906_at	PA3906	6.92	9.14	-4.68	0.00E+00	0.00E+00			gb|AE004091|AE004091:4374849-4375233 (+) /			Q9HXA7
PA4103_at	PA4103	7.21	8.44	-2.34	0.00E+00	0.00E+00			gb|AE004091|AE004091:4587319-4587949 (+) /			Q9HWS5
PA4177_at	PA4177	6.34	8.66	-4.97	0.00E+00	0.00E+00			gb|AE004091|AE004091:4673461-4673863 (+) /			Q9HWK4
PA3953_at	PA3953	6.8	8.42	-3.05	0.00E+00	0.00E+00			gb|AE004091|AE004091:4433410-4433992 (+) /			Q9HX63
PA4075_at	PA4075	9.11	7.35	3.4	0.00E+00	0.00E+00			gb|AE004091|AE004091:4553523-4554360 (-) //			Q9HWV3
PA4072_at	PA4072	8.75	7.67	2.11	0.00E+00	0.00E+00			gb|AE004091|AE004091:4549484-4550975 (-) //			Q9HWV6
PA4070_at	PA4070	5.27	8.67	-10.51	0.00E+00	0.00E+00			gb|AE004091|AE004091:4547820-4548771 (-) //			Q9HWV8
Pae_AF241171cds19	Pae_AF241171	5.54	7.44	-3.73	0.00E+00	0.00E+00						
PA4145_at	PA4145	6.62	8.05	-2.68	0.00E+00	0.00E+00			gb|AE004091|AE004091:4637808-4638699 (+) /			Q9HWN6
Pae_AF241171cds16	Pae_AF241171	5.99	7.97	-3.94	0.00E+00	0.00E+00						
PA4033_at	PA4033	7.31	8.8	-2.79	0.00E+00	0.00E+00			gb|AE004091|AE004091:4514078-4514348 (+) /			Q9HWZ4
PA4143_at	PA4143	9.37	8.21	2.24	0.00E+00	0.00E+00			gb|AE004091|AE004091:4634138-4636298 (+) /			Q9HWN8
Pae_AF241171cds14	Pae_AF241171	5.33	4.18	2.23	0.00E+00	0.00E+00						
PA4048_at	PA4048	7.82	5.81	4.03	0.00E+00	0.00E+00			gb|AE004091|AE004091:4530742-4531387 (-) //			Q9HWY0
PA4069_at	PA4069	8.3	7.1	2.31	0.00E+00	0.00E+00			gb|AE004091|AE004091:4546667-4547552 (-) //			Q9HWV9

PA4136_at	PA4136	8	6.22	3.43	0.00E+00	0.00E+00	gb|AE004091|AE004091:4625088-4626297 (-) //	Q9HWP5
PA4063_at	PA4063	6.28	8.79	-5.7	0.00E+00	0.00E+00	gb|AE004091|AE004091:4541271-4541862 (+) /	Q9HWW5
PA4065_at	PA4065	9.57	8.27	2.46	0.00E+00	0.00E+00	gb|AE004091|AE004091:4542657-4543923 (+) /	Q9HWW3
PA4066_at	PA4066	9.01	7.94	2.09	0.00E+00	0.00E+00	gb|AE004091|AE004091:4543940-4544459 (+) /	Q9HWW2
PA4154_at	PA4154	7.95	9.01	-2.1	0.00E+00	0.00E+00	gb|AE004091|AE004091:4647894-4648563 (-) //	Q9HWM7
PA4078_at	PA4078	9.78	8.7	2.11	0.00E+00	0.00E+00	gb|AE004091|AE004091:4555253-4558229 (+) /	Q9HWV0
PA4018_r_at	PA4018	7.72	8.76	-2.06	0.00E+00	0.00E+00	gb|AE004091|AE004091:4497572-4497848 (-) //	Q9HX09
PA4089_at	PA4089	6.06	8.54	-5.58	0.00E+00	0.00E+00	gb|AE004091|AE004091:4572310-4573072 (+) /	Q9HWT9
PA3957_at	PA3957	8.95	7.73	2.33	0.00E+00	0.00E+00	gb|AE004091|AE004091:4436491-4437328 (-) //	Q9HX59
PA4185_at	PA4185	7.89	9.73	-3.58	0.00E+00	0.00E+00	gb|AE004091|AE004091:4681420-4682149 (+) /	Q9HWJ6
PA3974_at	PA3974	9.42	8.23	2.28	0.00E+00	0.00E+00	gb|AE004091|AE004091:445328 Two-componen	Q9HX42
PA3978_at	PA3978	8.99	10.1	-2.16	0.00E+00	0.00E+00	gb|AE004091|AE004091:4458853-4459402 (+) /	Q51469; G3XD26
PA3979_at	PA3979	6.8	9.08	-4.87	0.00E+00	0.00E+00	gb|AE004091|AE004091:4459417-4459750 (-) //	Q9HX39
PA3980_at	PA3980	6.85	9.17	-4.99	0.00E+00	0.00E+00	gb|AE004091|AE004091:4459878-4461219 (+) /	Q51470
PA4099_at	PA4099	7.75	8.94	-2.27	0.00E+00	0.00E+00	gb|AE004091|AE004091:4581392-4582697 (+) /	Q9HWS9
PA3944_at	PA3944	5.67	8.18	-5.66	0.00E+00	0.00E+00	gb|AE004091|AE004091:4421801-4422380 (-) //	Q9HX72
PA4097_at	PA4097	6.38	8.04	-3.15	0.00E+00	0.00E+00	gb|AE004091|AE004091:4579509-4580568 (+) /	Q9HWT1
PA4168_at	PA4168	8	9.14	-2.21	0.00E+00	0.00E+00	gb|AE004091|AE004091:4663853-4666262 (+) /	Q9HWL3
PA4096_at	PA4096	9.14	8.12	2.03	0.00E+00	0.00E+00	gb|AE004091|AE004091:4578253-4579513 (+) /	Q9HWT2
PA4167_at	PA4167	6.84	8.24	-2.63	0.00E+00	0.00E+00	gb|AE004091|AE004091:4662692-4663511 (-) //	Q9HWL4
Pae_AF241171cds22	Pae_AF241171	6.77	4.53	4.73	0.00E+00	0.00E+00		
Pae_AF241171cds11	Pae_AF241171	5.14	7.79	-6.28	0.00E+00	0.00E+00		
PA4013_at	PA4013	7.8	9.52	-3.29	0.00E+00	0.00E+00	gb|AE004091|AE004091:4493214-4493925 (-) //	Q9HX14
PA4016_at	PA4016	8.3	5.32	7.88	0.00E+00	0.00E+00	gb|AE004091|AE004091:4495125-4496865 (+) /	Q9HX11
PA4169_at	PA4169	6.88	8.7	-3.55	0.00E+00	0.00E+00	gb|AE004091|AE004091:4666326-4666755 (-) //	Q9HWL2
PA3899_at	PA3899	7.34	8.82	-2.78	0.00E+00	0.00E+00	gb|AE004091|AE004091:4367282-4367792 (+) /	Q9HXB4
Pae_AF133699cds2_	Pae_AF133699	9.58	7.64	3.83	0.00E+00	0.00E+00		
PA3894_at	PA3894	7.95	9.16	-2.31	0.00E+00	0.00E+00	gb|AE004091|AE004091:4361493-4362984 (-) //	Q9HXB9
PA3460_at	PA3460	5.19	8.52	-10.08	0.00E+00	0.00E+00	gb|AE004091|AE004091:3867706-3869464 (+) /	Q9HYE6
PA3465_at	PA3465	7.08	8.4	-2.5	0.00E+00	0.00E+00	gb|AE004091|AE004091:3876195-3877911 (+) /	Q9HYE1
PA3470_i_at	PA3470	9.48	8.16	2.5	0.00E+00	0.00E+00	gb|AE004091|AE004091:3882979-3883438 (+) /	Q9HYD6
PA3472_at	PA3472	4.84	8.54	-13	0.00E+00	0.00E+00	gb|AE004091|AE004091:3885710-3886307 (+) /	Q9HYD4
PA3484_at	PA3484	7.84	9.14	-2.47	0.00E+00	0.00E+00	gb|AE004091|AE004091:3898277-3899504 (+) /	Q9HYC5
PA3490_at	PA3490	5.93	8.3	-5.18	0.00E+00	0.00E+00	gb|AE004091|AE004091:3907848-3908415 (+) /	Q9HYB9
PA3494_at	PA3494	7.48	9.01	-2.88	0.00E+00	0.00E+00	gb|AE004091|AE004091:3912409-3913132 (+) /	Q9HYB5
PA3498_at	PA3498	6.41	7.9	-2.8	0.00E+00	0.00E+00	gb|AE004091|AE004091:3915806-3916763 (-) //	Q9HYB1
PA3503_at	PA3503	7.02	5.7	2.49	0.00E+00	0.00E+00	gb|AE004091|AE004091:3918795-3919431 (-) // 100.0 //	
PA3506_at	PA3506	6.28	8.55	-4.82	0.00E+00	0.00E+00	gb|AE004091|AE004091:3922083-3923766 (-) //	Q9HYA3

Pae_AF241171cds43	Pae_AF241171	6.19	3.9	4.86	0.00E+00	0.00E+00		
Pae_AF241171cds40	Pae_AF241171	7.43	3.46	15.63	0.00E+00	0.00E+00		
PA3455_at	PA3455	5.96	8.59	-6.21	0.00E+00	0.00E+00	gb|AE004091|AE004091:3860674-3862165 (-) //	Q9HYF1
PA3509_at	PA3509	7.27	8.59	-2.51	0.00E+00	0.00E+00	gb|AE004091|AE004091:3925368-3926238 (-) //	Q9HYA0
Pae_AF241171cds39	Pae_AF241171	6.85	5.15	3.26	0.00E+00	0.00E+00		
PA3526_at	PA3526	5.06	8.98	-15.16	0.00E+00	0.00E+00	gb|AE004091|AE004091:3945996-3946962 (-) //	Q9HY83
PA3534_at	PA3534	6.69	9.07	-5.22	0.00E+00	0.00E+00	gb|AE004091|AE004091:3952499-3954608 (-) //	Q9HY76
Pae_AF241171cds31	Pae_AF241171	5.13	6.48	-2.55	0.00E+00	0.00E+00		
Pae_AF241171cds30	Pae_AF241171	8.66	6.7	3.89	0.00E+00	0.00E+00		
PA3552_at	PA3552	7.61	9.27	-3.15	0.00E+00	0.00E+00	gb|AE004091|AE004091:3979859-3981008 (+) /	Q9HY65
PA3555_at	PA3555	9.47	8.41	2.08	0.00E+00	0.00E+00	gb|AE004091|AE004091:3984005-3984893 (+) /	Q9HY62
PA3559_at	PA3559	4.76	8.41	-12.52	0.00E+00	0.00E+00	gb|AE004091|AE004091:3987289-3988684 (+) /	Q9HY58
PA3564_at	PA3564	5.91	8.12	-4.64	0.00E+00	0.00E+00	gb|AE004091|AE004091:3995840-3996518 (+) /	Q9HY53
PA3567_at	PA3567	4.69	9.49	-27.89	0.00E+00	0.00E+00	gb|AE004091|AE004091:3998136-3999150 (+) /	Q9HY50
PA3574_at	PA3574	6	8.37	-5.17	0.00E+00	0.00E+00	gb|AE004091|AE004091:4006509-4007148 (+) /	Q9HY46
PA3575_at	PA3575	6.85	8.7	-3.6	0.00E+00	0.00E+00	gb|AE004091|AE004091:4007506-4008037 (+) /	Q9HY45
PA3513_at	PA3513	5.01	8.07	-8.33	0.00E+00	0.00E+00	gb|AE004091|AE004091:3928330-3929338 (-) //	Q9HY96
PA3577_i_at	PA3577	8.61	9.83	-2.32	0.00E+00	0.00E+00	gb|AE004091|AE004091:4008937-4009051 (-) //	Q9HY43
PA3454_at	PA3454	6.58	9.01	-5.37	0.00E+00	0.00E+00	gb|AE004091|AE004091:3859337-3860522 (-) //	Q9HYF2
PA3449_at	PA3449	6.61	8.87	-4.79	0.00E+00	0.00E+00	gb|AE004091|AE004091:3854240-3855242 (-) //	Q9HYF7
PA3312_at	PA3312	9.85	8.55	2.46	0.00E+00	0.00E+00	gb|AE004091|AE004091:3714914-3715805 (-) //	Q9HYT2
PA3313_at	PA3313	7.24	8.85	-3.05	0.00E+00	0.00E+00	gb|AE004091|AE004091:3715803-3716811 (+) /	Q9HYT1
PA3322_at	PA3322	8.15	9.35	-2.29	0.00E+00	0.00E+00	gb|AE004091|AE004091:3724455-3725154 (-) //	Q9HYS3
PA3325_at	PA3325	7.79	9.16	-2.59	0.00E+00	0.00E+00	gb|AE004091|AE004091:3728239-3729127 (+) /	Q9HYS0
PA3338_at	PA3338	6.17	8	-3.54	0.00E+00	0.00E+00	gb|AE004091|AE004091:3747165-3747456 (-) //	Q9HYQ7
PA3346_at	PA3346	6.3	8.19	-3.72	0.00E+00	0.00E+00	gb|AE004091|AE004091:375765 Two-componei	Q9HYP9
PA3352_at	PA3352	7.57	9.25	-3.21	0.00E+00	0.00E+00	gb|AE004091|AE004091:3763181-3763652 (+) /	Q9HYP4
PA3356_at	PA3356	10.78	9.76	2.03	0.00E+00	0.00E+00	gb|AE004091|AE004091:3766616-3767858 (-) //	Q9HYP0
PA3358_at	PA3358	9.01	7.88	2.19	0.00E+00	0.00E+00	gb|AE004091|AE004091:3769586-3770465 (+) /	Q9HYN8
PA3360_at	PA3360	5.83	9.05	-9.35	0.00E+00	0.00E+00	gb|AE004091|AE004091:3771502-3772561 (-) //	Q9HYN6
PA3367_at	PA3367	7.95	4.67	9.69	0.00E+00	0.00E+00	gb|AE004091|AE004091:3778265-3778604 (+) /	Q9HYN4
Pae_AF241171cds45	Pae_AF241171	7.68	3.93	13.51	0.00E+00	0.00E+00		
PA3453_at	PA3453	8.28	9.41	-2.18	0.00E+00	0.00E+00	gb|AE004091|AE004091:3858543-3859209 (-) //	Q9HYF3
PA3370_at	PA3370	8.23	9.59	-2.57	0.00E+00	0.00E+00	gb|AE004091|AE004091:3779929-3780076 (+) /	Q9HYN1
PA3380_at	PA3380	5.33	8.26	-7.65	0.00E+00	0.00E+00	gb|AE004091|AE004091:378702(Phosphonate u	Q9HYM1
PA3383_at	PA3383	7.99	9	-2.01	0.00E+00	0.00E+00	gb|AE004091|AE004091:378909 Phosphonate u	Q9HYL8
PA3390_at	PA3390	8.34	6.99	2.54	0.00E+00	0.00E+00	gb|AE004091|AE004091:3793814-3794117 (-) //	Q9HYL5
PA3402_at	PA3402	6.36	8.74	-5.21	0.00E+00	0.00E+00	gb|AE004091|AE004091:3807245-3808217 (-) //	Q9HYK2

PA3404_at	PA3404	7.6	8.94	-2.54	0.00E+00	0.00E+00	gb|AE004091|AE004091:3809257-3810613 (-) // Q9HYK0
PA3409_at	PA3409	9.78	8.46	2.5	0.00E+00	0.00E+00	gb|AE004091|AE004091:3817545-3818532 (-) // Q9HYJ6
PA3419_at	PA3419	10.05	8.16	3.71	0.00E+00	0.00E+00	gb|AE004091|AE004091:3826018-3826831 (+) / Q9HYI6
PA3420_at	PA3420	7.51	8.99	-2.79	0.00E+00	0.00E+00	gb|AE004091|AE004091:3826837-3829321 (-) // Q9HYI5
PA3426_at	PA3426	6.2	8.27	-4.18	0.00E+00	0.00E+00	gb|AE004091|AE004091:3835578-3836349 (-) // Q9HYH9
PA3432_i_at	PA3432	9.59	8.24	2.55	0.00E+00	0.00E+00	gb|AE004091|AE004091:3840358-3840748 (-) // Q9HYH3
PA3442_at	PA3442	9.97	8.92	2.08	0.00E+00	0.00E+00	gb|AE004091|AE004091:3847973-3848798 (-) // Q9HYG4
PA3447_at	PA3447	6.21	7.76	-2.93	0.00E+00	0.00E+00	gb|AE004091|AE004091:3852666-3853416 (-) // Q9HYF9
PA3379_at	PA3379	6.23	7.31	-2.11	0.00E+00	0.00E+00	gb|AE004091|AE004091:378639: Phosphonate u Q9HYM2
PA3897_at	PA3897	8.79	6.83	3.91	0.00E+00	0.00E+00	gb|AE004091|AE004091:4365272-4366175 (-) // Q9HXB6
PA3579_at	PA3579	4.97	8.57	-12.13	0.00E+00	0.00E+00	gb|AE004091|AE004091:401052: Glycerolipid me Q9HY41
PA3592_at	PA3592	6.91	8.91	-4	0.00E+00	0.00E+00	gb|AE004091|AE004091:4025585-4026773 (-) // Q9HY34
PA3758_at	PA3758	6.42	8.47	-4.16	0.00E+00	0.00E+00	gb|AE004091|AE004091:4208167-4209259 (+) / Q9HXN7
PA3759_at	PA3759	9.71	8.7	2.01	0.00E+00	0.00E+00	gb|AE004091|AE004091:4209255-4210278 (+) / Q9HXN6
PA3760_at	PA3760	9.22	7.46	3.39	0.00E+00	0.00E+00	gb|AE004091|AE004091:4210294-4212823 (+) / Q9HXN5
PA3762_at	PA3762	6.1	9.06	-7.77	0.00E+00	0.00E+00	gb|AE004091|AE004091:4214761-4215079 (-) // Q9HXN3
PA3765_at	PA3765	6.97	8.76	-3.47	0.00E+00	0.00E+00	gb|AE004091|AE004091:4221212-4221797 (-) // Q9HXN0
PA3774_at	PA3774	6.87	8.24	-2.59	0.00E+00	0.00E+00	gb|AE004091|AE004091:4232257-4233400 (+) / Q9HXM1
Pae_AF241171cds25	Pae_AF241171	9.08	7.57	2.84	0.00E+00	0.00E+00	
PA3780_at	PA3780	8.23	6.42	3.49	0.00E+00	0.00E+00	gb|AE004091|AE004091:4238807-4239302 (+) / Q9HXL5
PA3782_at	PA3782	6.97	8.33	-2.56	0.00E+00	0.00E+00	gb|AE004091|AE004091:4241341-4242295 (-) // Q9HXL3
PA3786_at	PA3786	7.58	9.33	-3.38	0.00E+00	0.00E+00	gb|AE004091|AE004091:4244314-4244704 (-) // Q9HXK9
PA3799_at	PA3799	7.33	8.88	-2.94	0.00E+00	0.00E+00	gb|AE004091|AE004091:4257695-4259177 (-) // Q9HXJ8
PA3801_at	PA3801	9.82	8.59	2.35	0.00E+00	0.00E+00	gb|AE004091|AE004091:4260398-4261043 (-) // Q9HXJ6
PA3750_at	PA3750	5.61	7.76	-4.44	0.00E+00	0.00E+00	gb|AE004091|AE004091:4202572-4203322 (-) // Q9HXP4
PA3830_at	PA3830	7.58	8.72	-2.2	0.00E+00	0.00E+00	gb|AE004091|AE004091:4287793-4288606 (-) // Q9HXH2
PA3846_at	PA3846	8	9.08	-2.11	0.00E+00	0.00E+00	gb|AE004091|AE004091:4307265-4307808 (-) // Q9HXG0
PA3851_at	PA3851	6.46	7.87	-2.67	0.00E+00	0.00E+00	gb|AE004091|AE004091:4311950-4312703 (-) // Q9HXF5
PA3852_at	PA3852	9	10.08	-2.11	0.00E+00	0.00E+00	gb|AE004091|AE004091:4312706-4313642 (-) // Q9HXF4
PA3856_at	PA3856	9.04	7.68	2.57	0.00E+00	0.00E+00	gb|AE004091|AE004091:4315555-4316038 (-) // Q9HXF0
PA3858_at	PA3858	7.33	8.85	-2.87	0.00E+00	0.00E+00	gb|AE004091|AE004091:4316936-4317962 (-) // Q9HXE8
PA3860_at	PA3860	10.6	9.36	2.36	0.00E+00	0.00E+00	gb|AE004091|AE004091:4318921-4320820 (-) // Q9HXE6
PA3882_at	PA3882	6.88	8.43	-2.94	0.00E+00	0.00E+00	gb|AE004091|AE004091:4348665-4349415 (+) / Q9HXD0
PA3883_at	PA3883	5.17	6.39	-2.33	0.00E+00	0.00E+00	gb|AE004091|AE004091:4349501-4350332 (+) / Q9HXC9
PA3884_at	PA3884	9.25	7.01	4.72	0.00E+00	0.00E+00	gb|AE004091|AE004091:4350352-4350739 (+) / Q9HXC8
PA3885_at	PA3885	5.93	7.56	-3.1	0.00E+00	0.00E+00	gb|AE004091|AE004091:4350833-4351490 (+) / Q9HXC7
PA3888_at	PA3888	5.82	8.79	-7.88	0.00E+00	0.00E+00	gb|AE004091|AE004091:4354543-4355266 (+) / Q9HXC5
PA3889_at	PA3889	6.4	9.3	-7.42	0.00E+00	0.00E+00	gb|AE004091|AE004091:4355265-4356201 (+) / Q9HXC4

PA3843_at	PA3843	6.42	9.1	-6.41	0.00E+00	0.00E+00	gb|AE004091|AE004091:4305063-4305426 (+) // 100.0 //
PA3580_at	PA3580	7.15	8.76	-3.04	0.00E+00	0.00E+00	gb|AE004091|AE004091:4012070-4012541 (-) // Q51388; G3XCT4
PA3749_at	PA3749	5.8	8.39	-5.99	0.00E+00	0.00E+00	gb|AE004091|AE004091:4201251-4202565 (+) / Q9HXP5
Pae_AF241171cds27	Pae_AF241171	6.49	5.23	2.39	0.00E+00	0.00E+00	
PA3596_at	PA3596	8.35	9.43	-2.11	0.00E+00	0.00E+00	gb|AE004091|AE004091:4031164-4032238 (-) // Q9HY30
PA3597_at	PA3597	5.56	8.19	-6.18	0.00E+00	0.00E+00	gb|AE004091|AE004091:4032327-4033650 (-) // Q9HY29
PA3598_at	PA3598	6.72	8.64	-3.79	0.00E+00	0.00E+00	gb|AE004091|AE004091:4033849-4034665 (-) // Q9HY28
PA3606_at	PA3606	8.5	7.3	2.3	0.00E+00	0.00E+00	gb|AE004091|AE004091:4040103-4040820 (-) // Q9HY20
PA3614_at	PA3614	9.87	8.48	2.61	0.00E+00	0.00E+00	gb|AE004091|AE004091:4048518-4049922 (+) / Q9HY12
PA3623_at	PA3623	6.58	8.74	-4.47	0.00E+00	0.00E+00	gb|AE004091|AE004091:4059017-4059911 (-) // P45682
PA3628_at	PA3628	8.34	9.73	-2.62	0.00E+00	0.00E+00	gb|AE004091|AE004091:4062969-4063821 (-) // Q9HY02
PA3630_at	PA3630	5.51	8.47	-7.74	0.00E+00	0.00E+00	gb|AE004091|AE004091:4065118-4066027 (+) / Q9HY00
Pae_AF241171cds28	Pae_AF241171	5.69	4.08	3.04	0.00E+00	0.00E+00	
PA3660_at	PA3660	6.76	8.78	-4.05	0.00E+00	0.00E+00	gb|AE004091|AE004091:4100103-4101849 (-) // Q9HXX9
PA3663_at	PA3663	7.55	8.77	-2.33	0.00E+00	0.00E+00	gb|AE004091|AE004091:4103075-4103429 (-) // Q9HXX6
PA3664_at	PA3664	8.55	9.86	-2.49	0.00E+00	0.00E+00	gb|AE004091|AE004091:4103731-4104079 (+) / Q9HXX5
PA3747_at	PA3747	6.28	8.91	-6.17	0.00E+00	0.00E+00	gb|AE004091|AE004091:4198842-4199643 (+) / Q9HXP7
PA3679_at	PA3679	6.74	9.24	-5.66	0.00E+00	0.00E+00	gb|AE004091|AE004091:4121113-4122391 (-) // Q9HXW1
PA3685_at	PA3685	7.45	5.74	3.27	0.00E+00	0.00E+00	gb|AE004091|AE004091:4126163-4126844 (-) // Q9HXV5
PA3689_at	PA3689	9.84	8.49	2.55	0.00E+00	0.00E+00	gb|AE004091|AE004091:4130951-4131422 (-) // Q9HXV1
PA3693_at	PA3693	6.42	8.37	-3.86	0.00E+00	0.00E+00	gb|AE004091|AE004091:4135451-4135973 (-) // Q9HXU7
PA3695_at	PA3695	7.77	9.06	-2.45	0.00E+00	0.00E+00	gb|AE004091|AE004091:4136289-4137195 (-) // Q9HXU5
PA3698_at	PA3698	6.19	9.22	-8.21	0.00E+00	0.00E+00	gb|AE004091|AE004091:4139310-4139862 (-) // Q9HXU2
PA3710_at	PA3710	6.8	8.94	-4.39	0.00E+00	0.00E+00	gb|AE004091|AE004091:4154338-4156012 (-) // Q9HXT1
PA3711_at	PA3711	7.69	6.41	2.42	0.00E+00	0.00E+00	gb|AE004091|AE004091:4156183-4157089 (+) / Q9HXT0
PA3713_at	PA3713	7.65	8.89	-2.36	0.00E+00	0.00E+00	gb|AE004091|AE004091:4158142-4160005 (+) / Q9HXS8
PA3721_at	PA3721	6.73	7.96	-2.35	0.00E+00	0.00E+00	gb|AE004091|AE004091:4166517-4167159 (+) / Q9HXS0
PA3722_at	PA3722	7.53	9.06	-2.89	0.00E+00	0.00E+00	gb|AE004091|AE004091:4167171-4167522 (-) // Q9HXR9
PA3730_at	PA3730	6.38	7.76	-2.6	0.00E+00	0.00E+00	gb|AE004091|AE004091:4181377-4182019 (-) // Q9HXR2
PA4189_at	PA4189	6.65	8.69	-4.11	0.00E+00	0.00E+00	gb|AE004091|AE004091:4685993-4687484 (+) / Q9HWJ2
PA3680_at	PA3680	5.29	8.14	-7.2	0.00E+00	0.00E+00	gb|AE004091|AE004091:4122419-4123205 (-) // Q9HXW0
PA4195_at	PA4195	7.93	9.48	-2.93	0.00E+00	0.00E+00	gb|AE004091|AE004091:4692125-4692956 (-) // Q9HWI6
PA4534_at	PA4534	6.51	8.27	-3.39	0.00E+00	0.00E+00	gb|AE004091|AE004091:5075876-5076302 (-) // Q9HVP3
PA4198_at	PA4198	7.52	9.12	-3.02	0.00E+00	0.00E+00	gb|AE004091|AE004091:4696280-4697903 (+) / Q9HWI3
PA5232_at	PA5232	10.75	8.25	5.65	0.00E+00	0.00E+00	gb|AE004091|AE004091:5889828-5890902 (-) // Q9HTV8
PA5245_at	PA5245	5.97	8.28	-4.96	0.00E+00	0.00E+00	gb|AE004091|AE004091:5906625-5907294 (+) / Q9HTU9
PA5246_at	PA5246	6.13	7.31	-2.28	0.00E+00	0.00E+00	gb|AE004091|AE004091:5907389-5907863 (+) / Q9HTU8
PA5251_at	PA5251	6.63	8.73	-4.29	0.00E+00	0.00E+00	gb|AE004091|AE004091:5912026-5912605 (-) // Q9HTU3

PA5252_at	PA5252	8.89	7.34	2.93	0.00E+00	0.00E+00	gb|AE004091|AE004091:5912604-5914521 (-) //	Q9HTU2
Pae_AF147795cds9_	Pae_AF147795	5.48	4.44	2.06	0.00E+00	0.00E+00		
PA5273_at	PA5273	6.2	7.65	-2.75	0.00E+00	0.00E+00	gb|AE004091|AE004091:5939262-5939976 (+) /	Q9HTS7
PA5275_at	PA5275	8.16	6.58	2.98	0.00E+00	0.00E+00	gb|AE004091|AE004091:5940746-5941082 (-) //	Q9HTS5
PA5279_at	PA5279	8.17	9.53	-2.55	0.00E+00	0.00E+00	gb|AE004091|AE004091:5943605-5944307 (+) /	G3XD70
PA5283_at	PA5283	7.35	9.03	-3.2	0.00E+00	0.00E+00	gb|AE004091|AE004091:5947294-5948671 (+) /	Q9HTS1
PA5284_at	PA5284	7.63	3.96	12.76	0.00E+00	0.00E+00	gb|AE004091|AE004091:5948695-5949610 (-) //	Q9HTS0
PA5286_at	PA5286	8.82	7.47	2.56	0.00E+00	0.00E+00	gb|AE004091|AE004091:5950428-5950854 (-) //	Q9HTR8
PA5228_at	PA5228	11	9.7	2.46	0.00E+00	0.00E+00	gb|AE004091|AE004091:5884507-5885119 (+) /	Q9HTW2
Pae_AF147795cds8_	Pae_AF147795	6.28	7.52	-2.35	0.00E+00	0.00E+00		
PA5298_at	PA5298	5.9	8.91	-8.05	0.00E+00	0.00E+00	gb|AE004091|AE004091:5966705-5967278 (+) /	Q9HTQ6
PA5301_at	PA5301	8.19	9.59	-2.64	0.00E+00	0.00E+00	gb|AE004091|AE004091:5969986-5970535 (-) //	Q9HTQ3
PA5311_at	PA5311	6.83	8.34	-2.84	0.00E+00	0.00E+00	gb|AE004091|AE004091:5980786-5981950 (-) //	Q9HTP3
PA5318_at	PA5318	6.6	8.51	-3.75	0.00E+00	0.00E+00	gb|AE004091|AE004091:5988080-5988629 (+) /	Q9HTN6
Pae_AF147795cds5_	Pae_AF147795	3.51	5.05	-2.91	0.00E+00	0.00E+00		
PA5342_at	PA5342	7.5	9.32	-3.53	0.00E+00	0.00E+00	gb|AE004091|AE004091:6010257-6011058 (-) //	Q9HTL6
PA5357_at	PA5357	9.36	8.17	2.29	0.00E+00	0.00E+00	gb|AE004091|AE004091:602474 Ubiquinone bic	Q9HTK1
PA5362_at	PA5362	6.34	8.17	-3.55	0.00E+00	0.00E+00	gb|AE004091|AE004091:6028975-6030316 (+) /	Q9HTJ8
PA5370_at	PA5370	8.14	9.39	-2.38	0.00E+00	0.00E+00	gb|AE004091|AE004091:6045309-6046626 (-) //	Q9HTJ4
PA5371_at	PA5371	8.29	9.77	-2.8	0.00E+00	0.00E+00	gb|AE004091|AE004091:6046913-6047318 (+) /	Q9HTJ3
PA5376_at	PA5376	9.17	7.22	3.87	0.00E+00	0.00E+00	gb|AE004091|AE004091:6053411-6054590 (-) //	Q9HTI8
PA5385_at	PA5385	6.59	8.58	-3.98	0.00E+00	0.00E+00	gb|AE004091|AE004091:6063352-6063832 (-) //	Q9HTH9
PA5294_at	PA5294	6.42	5.4	2.02	0.00E+00	0.00E+00	gb|AE004091|AE004091:5959473-5960940 (+) /	Q9HTR0
PA5391_at	PA5391	6.05	7.11	-2.09	0.00E+00	0.00E+00	gb|AE004091|AE004091:6069155-6069830 (-) //	Q9HTH3
PA5225_at	PA5225	8.26	6.87	2.64	0.00E+00	0.00E+00	gb|AE004091|AE004091:5883022-5883577 (-) //	Q9HTW5
PA5220_at	PA5220	10.45	9.01	2.7	0.00E+00	0.00E+00	gb|AE004091|AE004091:5877895-5878717 (+) /	Q9HTX0
PA5104_at	PA5104	7.38	9.72	-5.06	0.00E+00	0.00E+00	gb|AE004091|AE004091:5748012-5748603 (-) /	Q9HU79
PA5108_at	PA5108	9.65	7.92	3.31	0.00E+00	0.00E+00	gb|AE004091|AE004091:5751526-5751784 (-) //	Q9HU75
Pae_L37109cds2_at	Pae_L37109cds	7.55	6.18	2.57	0.00E+00	0.00E+00		
PA5109_at	PA5109	6.28	7.41	-2.18	0.00E+00	0.00E+00	gb|AE004091|AE004091:5751856-5752459 (-) //	Q9HU74
PA5114_at	PA5114	6.79	7.8	-2.02	0.00E+00	0.00E+00	gb|AE004091|AE004091:5757737-5761343 (-) //	Q9HU70
PA5122_at	PA5122	8.1	9.44	-2.54	0.00E+00	0.00E+00	gb|AE004091|AE004091:5770936-5771458 (+) /	Q9HU62
PA5127_at	PA5127	4.33	6.85	-5.75	0.00E+00	0.00E+00	gb|AE004091|AE004091:5776085-5776547 (+) /	Q9HU57
PA5134_at	PA5134	7.25	8.91	-3.16	0.00E+00	0.00E+00	gb|AE004091|AE004091:5782277-5783588 (+) /	Q9HU50
PA5135_at	PA5135	7.16	8.73	-2.98	0.00E+00	0.00E+00	gb|AE004091|AE004091:5783587-5784361 (+) /	Q9HU49
PA5137_at	PA5137	8.59	7.3	2.43	0.00E+00	0.00E+00	gb|AE004091|AE004091:5785947-5786703 (-) //	Q9HU47
PA5138_at	PA5138	6.27	8.24	-3.93	0.00E+00	0.00E+00	gb|AE004091|AE004091:5786852-5787605 (-) //	Q9HU46
PA5144_i_at	PA5144	9.07	7.99	2.11	0.00E+00	0.00E+00	gb|AE004091|AE004091:5791836-5792235 (+) /	Q9HU40

PA5221_at	PA5221	4.72	7.84	-8.7	0.00E+00	0.00E+00	gb|AE004091|AE004091:5878753-5879971 (-) // Q9HTW9
PA5145_at	PA5145	6.88	8.11	-2.34	0.00E+00	0.00E+00	gb|AE004091|AE004091:5792504-5793611 (+) / Q9HU39
Pae_L37109cds1_at	Pae_L37109cds	8.03	6.64	2.62	0.00E+00	0.00E+00	
PA5186_at	PA5186	10.09	8.75	2.53	0.00E+00	0.00E+00	gb|AE004091|AE004091:5837914-5839078 (-) // Q9HU03
Pae_AF080442cds2_	Pae_AF080442	8.08	4.59	11.19	0.00E+00	0.00E+00	
PA5188_at	PA5188	6.35	7.76	-2.65	0.00E+00	0.00E+00	gb|AE004091|AE004091:5840896-5842132 (-) // Q9HU01
PA5198_at	PA5198	5.6	8.86	-9.61	0.00E+00	0.00E+00	gb|AE004091|AE004091:5850238-5851162 (+) / Q9HTZ1
PA5208_at	PA5208	7.24	8.76	-2.87	0.00E+00	0.00E+00	gb|AE004091|AE004091:5863033-5863711 (-) // Q9HTY2
PA5212_i_at	PA5212	8.14	9.54	-2.62	0.00E+00	0.00E+00	gb|AE004091|AE004091:5867816-5868146 (+) / Q9HTX8
PA5216_at	PA5216	9.33	8.22	2.16	0.00E+00	0.00E+00	gb|AE004091|AE004091:5872899-5874519 (-) // Q9HTX4
Pae_AF043558cds_a	Pae_AF043558	7.25	6.04	2.32	0.00E+00	0.00E+00	
Pae_AF147795cds11	Pae_AF147795	4.96	6.24	-2.42	0.00E+00	0.00E+00	
PA5218_at	PA5218	6.42	8.59	-4.51	0.00E+00	0.00E+00	gb|AE004091|AE004091:5875652-5876567 (-) // Q9HTX2
Pae_AF147795cds10	Pae_AF147795	4.62	7.85	-9.39	0.00E+00	0.00E+00	
PA5156_at	PA5156	7.4	8.83	-2.69	0.00E+00	0.00E+00	gb|AE004091|AE004091:5803866-5804802 (+) // 100.0 //
PA5392_at	PA5392	8.8	7.57	2.35	0.00E+00	0.00E+00	gb|AE004091|AE004091:6069843-6070266 (-) // 100.0 //
PA5393_at	PA5393	7.47	8.52	-2.06	0.00E+00	0.00E+00	gb|AE004091|AE004091:6070265-6071618 (-) // Q9HTH1
PA5398_at	PA5398	5.4	8.41	-8.05	0.00E+00	0.00E+00	gb|AE004091|AE004091:6075879-6077940 (+) / Q9HTG6
ig_2250595_225127	ig_2250595_22	6	7.24	-2.36	0.00E+00	0.00E+00	gb|AE004091|AE004091:2250595-2251276 (+) // 100.0 //
ig_3115632_311665	ig_3115632_31	7.29	8.69	-2.65	0.00E+00	0.00E+00	gb|AE004091|AE004091:3115632-3116654 (+) // 100.0 //
ig_5207621_520846	ig_5207621_52	8.04	9.19	-2.22	0.00E+00	0.00E+00	gb|AE004091|AE004091:5207621-5208464 (+) // 100.0 //
ig_5308424_530932	ig_5308424_53	10.53	12.11	-2.98	0.00E+00	0.00E+00	gb|AE004091|AE004091:5308424-5309326 (+) // 100.0 //
ig_5457716_545849	ig_5457716_54	3.75	7.76	-16.15	0.00E+00	0.00E+00	gb|AE004091|AE004091:5457716-5458500 (+) // 100.0 //
ig_5513111_551373	ig_5513111_55	4.4	6.97	-5.95	0.00E+00	0.00E+00	gb|AE004091|AE004091:5513111-5513732 (+) // 100.0 //
ig_5820113_582090	ig_5820113_58	7.26	4.83	5.41	0.00E+00	0.00E+00	gb|AE004091|AE004091:5820113-5820910 (+) // 100.0 //
ig_65339_64729_at	ig_65339_6472	10.15	9.11	2.05	0.00E+00	0.00E+00	gb|AE004091|AE004091:64729-65340 (-) // 100.0 //
ig_189120_188448_	ig_189120_188	9.74	8.67	2.09	0.00E+00	0.00E+00	gb|AE004091|AE004091:188448-189121 (-) // 100.0 //
ig_518083_517462_	ig_518083_517	8.53	4.36	17.97	0.00E+00	0.00E+00	gb|AE004091|AE004091:517462-518084 (-) // 100.0 //
ig_774416_773696_	ig_774416_773	8.31	9.62	-2.49	0.00E+00	0.00E+00	gb|AE004091|AE004091:773696-774417 (-) // 100.0 //
ig_991830_991198_	ig_991830_991	6.44	4.95	2.82	0.00E+00	0.00E+00	gb|AE004091|AE004091:991203-991831 (-) // 99.21 //
ig_2226144_222687	ig_2226144_22	5.82	8.02	-4.61	0.00E+00	0.00E+00	gb|AE004091|AE004091:2226144-2226880 (+) // 100.0 //
ig_1087843_108709	ig_1087843_10	8.77	5.45	9.98	0.00E+00	0.00E+00	gb|AE004091|AE004091:1087095-1087844 (-) // 100.0 //
ig_2928538_292792	ig_2928538_29	4.98	6.06	-2.11	0.00E+00	0.00E+00	gb|AE004091|AE004091:2927920-2928539 (-) // 100.0 //
ig_3051956_305134	ig_3051956_30	7.54	9.16	-3.08	0.00E+00	0.00E+00	gb|AE004091|AE004091:3051348-3051957 (-) // 100.0 //
ig_3123598_312258	ig_3123598_31	7.57	9.13	-2.93	0.00E+00	0.00E+00	gb|AE004091|AE004091:3122585-3123599 (-) // 100.0 //
ig_3962824_396192	ig_3962824_39	6.86	4.45	5.32	0.00E+00	0.00E+00	gb|AE004091|AE004091:3961922-3962825 (-) // 100.0 //
ig_4510970_450999	ig_4510970_45	6.41	8.56	-4.42	0.00E+00	0.00E+00	gb|AE004091|AE004091:4509996-4510971 (-) // 100.0 //
ig_5458499_545771	ig_5458499_54	7.61	9.05	-2.72	0.00E+00	0.00E+00	gb|AE004091|AE004091:5457716-5458500 (-) // 100.0 //

ig_5513731_551311	ig_5513731_55	8.55	7.53	2.02	0.00E+00	0.00E+00	gb|AE004091|AE004091:5513111-5513732 (-) // 100.0 //
Pae_AF291700cds_a	Pae_AF291700	5.5	6.77	-2.41	0.00E+00	0.00E+00	
Pae_AF133699cds4_	Pae_AF133699	6.34	4.34	4	0.00E+00	0.00E+00	
Pae_AF191564cds4_	Pae_AF191564	4.89	7.22	-5.02	0.00E+00	0.00E+00	
Pae_AF140629cds1_	Pae_AF140629	5.66	7.05	-2.62	0.00E+00	0.00E+00	
Pae_AF140629cds2_	Pae_AF140629	8.25	6.61	3.1	0.00E+00	0.00E+00	
ig_2894451_289382	ig_2894451_28	9.76	7.4	5.14	0.00E+00	0.00E+00	gb|AE004091|AE004091:2893827-2894452 (-) // 100.0 //
ig_2164548_216521	ig_2164548_21	6.52	8.13	-3.05	0.00E+00	0.00E+00	gb|AE004091|AE004091:2164548-2165214 (+) // 100.0 //
ig_2116265_211703	ig_2116265_21	7.78	8.84	-2.08	0.00E+00	0.00E+00	gb|AE004091|AE004091:2116265-2117031 (+) // 100.0 //
ig_1637696_163837	ig_1637696_16	6.75	8.91	-4.45	0.00E+00	0.00E+00	gb|AE004091|AE004091:1637696-1638380 (+) // 100.0 //
PA5400_at	PA5400	6.43	7.64	-2.32	0.00E+00	0.00E+00	gb|AE004091|AE004091:6080369-6081356 (+) // 100.0 //
PA5402_at	PA5402	5.12	7.29	-4.5	0.00E+00	0.00E+00	gb|AE004091|AE004091:6082289-6082859 (+) /, Q9HTG2
PA5403_at	PA5403	6.84	8.23	-2.63	0.00E+00	0.00E+00	gb|AE004091|AE004091:6082866-6083073 (+) /, Q9HTG1
PA5422_at	PA5422	4.95	6	-2.07	0.00E+00	0.00E+00	gb|AE004091|AE004091:6103166-6104123 (+) /, Q9HTE2
PA5431_at	PA5431	6.35	8.56	-4.62	0.00E+00	0.00E+00	gb|AE004091|AE004091:6112298-6113774 (-) // Q9HTD5
PA5435_at	PA5435	6.7	8.37	-3.17	0.00E+00	0.00E+00	gb|AE004091|AE004091:611649: Pyruvate metal Q9HTD1
PA5437_at	PA5437	8.18	9.3	-2.19	0.00E+00	0.00E+00	gb|AE004091|AE004091:6119960-6120896 (+) /, Q9HTC9
PA5441_at	PA5441	9.18	7.7	2.79	0.00E+00	0.00E+00	gb|AE004091|AE004091:6125795-6127997 (-) // Q9HTC5
PA5444_at	PA5444	7.93	9.54	-3.06	0.00E+00	0.00E+00	gb|AE004091|AE004091:6133311-6133740 (+) /, Q9HTC3
PA5464_at	PA5464	9.41	8.11	2.47	0.00E+00	0.00E+00	gb|AE004091|AE004091:6152717-6154676 (+) /, Q9HTA6
PA5470_at	PA5470	9.2	8.02	2.26	0.00E+00	0.00E+00	gb|AE004091|AE004091:6158948-6159563 (-) // Q9HTA0
PA5509_at	PA5509	6.61	8.46	-3.62	0.00E+00	0.00E+00	gb|AE004091|AE004091:6200965-6201634 (+) /, Q9HT64
PA5511_at	PA5511	5.63	8.34	-6.56	0.00E+00	0.00E+00	gb|AE004091|AE004091:620310: Two-componer Q9HT62
Pae_AF147795cds3_	Pae_AF147795	7.3	4.03	9.68	0.00E+00	0.00E+00	
PA5522_at	PA5522	6.57	8.59	-4.06	0.00E+00	0.00E+00	gb|AE004091|AE004091:6214433-6215801 (-) // Q9HT51
PA5525_at	PA5525	8.95	7.77	2.27	0.00E+00	0.00E+00	gb|AE004091|AE004091:6218099-6218840 (+) /, Q9HT48
PA5528_at	PA5528	6.64	8.35	-3.26	0.00E+00	0.00E+00	gb|AE004091|AE004091:6219884-6220739 (-) // Q9HT45
PA5529_at	PA5529	9.21	7.85	2.57	0.00E+00	0.00E+00	gb|AE004091|AE004091:6221100-6222858 (+) /, Q9HT44
PA5537_at	PA5537	6.34	8.9	-5.9	0.00E+00	0.00E+00	gb|AE004091|AE004091:6229974-6230391 (+) /, Q9HT37
Pae_AF147795cds2_	Pae_AF147795	6.37	7.93	-2.96	0.00E+00	0.00E+00	
Pae_AF133697cds_a	Pae_AF133697	7.17	2.13	32.83	0.00E+00	0.00E+00	
PA5566_at	PA5566	9.39	8.04	2.54	0.00E+00	0.00E+00	gb|AE004091|AE004091:6259670-6260054 (-) // Q9HT08
ig_223454_224101_	ig_223454_224	7.92	9.15	-2.36	0.00E+00	0.00E+00	gb|AE004091|AE004091:223454-224102 (+) // 100.0 //
ig_545644_546334_	ig_545644_546	5.45	7.84	-5.23	0.00E+00	0.00E+00	gb|AE004091|AE004091:545644-546335 (+) // 100.0 //
ig_901046_901934_	ig_901046_901	7.96	8.99	-2.04	0.00E+00	0.00E+00	gb|AE004091|AE004091:901046-901935 (+) // 100.0 //
ig_1254309_125504	ig_1254309_12	6.97	8.86	-3.72	0.00E+00	0.00E+00	gb|AE004091|AE004091:1254309-1255043 (+) // 100.0 //
ig_1489095_148981	ig_1489095_14	8.06	9.15	-2.13	0.00E+00	0.00E+00	gb|AE004091|AE004091:1489095-1489816 (+) // 100.0 //
Pae_AF027291cds_a	Pae_AF027291	8.46	5.8	6.35	0.00E+00	0.00E+00	

PA5102_at	PA5102	10.15	7.99	4.44	0.00E+00	0.00E+00	gb|AE004091|AE004091:5745894-5746833 (-) // Q9HU81
PA5094_at	PA5094	8.88	9.97	-2.13	0.00E+00	0.00E+00	gb|AE004091|AE004091:5735642-5736473 (-) // Q9HU89
PA5088_at	PA5088	6.46	8.17	-3.28	0.00E+00	0.00E+00	gb|AE004091|AE004091:5726356-5727238 (-) // Q9HU95
Pae_L81176cds5_at	Pae_L81176cds	5.89	3.18	6.53	0.00E+00	0.00E+00	
PA4489_at	PA4489	5.07	8.59	-11.46	0.00E+00	0.00E+00	gb|AE004091|AE004091:5020401-5024952 (-) // Q9HVT2
PA4491_at	PA4491	7.85	8.94	-2.13	0.00E+00	0.00E+00	gb|AE004091|AE004091:5025613-5027383 (-) // Q9HVT0
PA4493_at	PA4493	10.25	8.79	2.76	0.00E+00	0.00E+00	gb|AE004091|AE004091:502839 Two-componen Q9HVS8
PA4494_at	PA4494	6.34	7.87	-2.87	0.00E+00	0.00E+00	gb|AE004091|AE004091:502897 Two-componen Q9HVS7
PA4495_at	PA4495	10.93	9.81	2.18	0.00E+00	0.00E+00	gb|AE004091|AE004091:5030524-5031235 (+) / Q9HVS6
Pae_L81176cds4_at	Pae_L81176cds	8.1	6.34	3.39	0.00E+00	0.00E+00	
PA4514_at	PA4514	8.38	9.4	-2.03	0.00E+00	0.00E+00	gb|AE004091|AE004091:5053615-5055877 (-) // O68590; G3XCY8
Pae_AF035937cds9_	Pae_AF035937	4.19	3.16	2.03	0.00E+00	0.00E+00	
Pae_AF035937cds8_	Pae_AF035937	4.99	7.45	-5.47	0.00E+00	0.00E+00	
PA4531_at	PA4531	7.75	8.94	-2.27	0.00E+00	0.00E+00	gb|AE004091|AE004091:5074408-5074618 (-) // Q9HVP6
Pae_AF133699cds3_	Pae_AF133699	5.95	4.86	2.12	0.00E+00	0.00E+00	
PA4473_at	PA4473	8.07	9.12	-2.06	0.00E+00	0.00E+00	gb|AE004091|AE004091:5001821-5002424 (+) / Q9HVU8
PA4539_at	PA4539	7.79	8.81	-2.03	0.00E+00	0.00E+00	gb|AE004091|AE004091:5079116-5080208 (-) // Q9HVN8
PA4543_at	PA4543	7.43	8.65	-2.33	0.00E+00	0.00E+00	gb|AE004091|AE004091:5090127-5090856 (-) // P33663
Pae_AF035937cds7_	Pae_AF035937	3.83	7.19	-10.24	0.00E+00	0.00E+00	
PA4573_at	PA4573	7.65	9.2	-2.92	0.00E+00	0.00E+00	gb|AE004091|AE004091:5121336-5121642 (-) // Q9HVL1
PA4586_at	PA4586	7.05	9.31	-4.77	0.00E+00	0.00E+00	gb|AE004091|AE004091:5135815-5136193 (+) / Q9HVJ8
Pae_AF035937cds5_	Pae_AF035937	4.57	2.94	3.1	0.00E+00	0.00E+00	
PA4595_at	PA4595	8.37	9.62	-2.38	0.00E+00	0.00E+00	gb|AE004091|AE004091:5146906-5148571 (-) // Q9HVJ1
PA4603_at	PA4603	8.89	7.77	2.18	0.00E+00	0.00E+00	gb|AE004091|AE004091:5162068-5162449 (-) // Q9HVI6
PA3305_at	PA3305	10.09	9.01	2.12	0.00E+00	0.00E+00	gb|AE004091|AE004091:3703166-3705161 (-) // Q9HYT9
PA4608_at	PA4608	10.48	9.43	2.07	0.00E+00	0.00E+00	gb|AE004091|AE004091:5166899-5167277 (+) / Q9HVI1
PA4612_at	PA4612	9.28	7.37	3.74	0.00E+00	0.00E+00	gb|AE004091|AE004091:5169579-5170131 (-) // G3XD34
PA4618_at	PA4618	6.35	8.34	-3.97	0.00E+00	0.00E+00	gb|AE004091|AE004091:5176679-5177651 (-) // Q9HVH3
PA4622_at	PA4622	6.43	9.23	-6.98	0.00E+00	0.00E+00	gb|AE004091|AE004091:5182578-5183790 (+) / Q9HVG9
PA4540_at	PA4540	7.05	9.3	-4.76	0.00E+00	0.00E+00	gb|AE004091|AE004091:5080756-5082394 (+) / Q9HVN7
PA4435_at	PA4435	6.22	7.77	-2.92	0.00E+00	0.00E+00	gb|AE004091|AE004091:4967312-4968458 (-) // Q9HVY0
PA4420_at	PA4420	6.86	9.06	-4.6	0.00E+00	0.00E+00	gb|AE004091|AE004091:4954629-4955571 (-) // Q9HVZ5
PA4405_at	PA4405	6.36	8.59	-4.67	0.00E+00	0.00E+00	gb|AE004091|AE004091:4937819-4938215 (+) / Q9HW03
PA4218_at	PA4218	6.7	8.28	-2.99	0.00E+00	0.00E+00	gb|AE004091|AE004091:4721613-4722858 (-) // Q9HWG8
Pae_AF241171cds10	Pae_AF241171	7.2	4.33	7.32	0.00E+00	0.00E+00	
PA4278_at	PA4278	6.68	7.85	-2.24	0.00E+00	0.00E+00	gb|AE004091|AE004091:4786020-4786725 (-) // Q9HWC2
Pae_AF241171cds9_	Pae_AF241171	5.54	7.48	-3.84	0.00E+00	0.00E+00	
PA4287_at	PA4287	6.91	7.95	-2.06	0.00E+00	0.00E+00	gb|AE004091|AE004091:4809546-4810437 (-) // Q9HWB3

Pae_AF241171cds8_	Pae_AF241171	6.68	7.76	-2.12	0.00E+00	0.00E+00			
PA4288_at	PA4288	8.31	7.17	2.21	0.00E+00	0.00E+00	gb|AE004091|AE004091:4810537-4811341 (+) /		Q9HWB2
PA4291_at	PA4291	7.81	9.28	-2.77	0.00E+00	0.00E+00	gb|AE004091|AE004091:4814212-4814899 (+) /		Q9HWA9
Pae_AF241171cds7_	Pae_AF241171	9.72	8.35	2.58	0.00E+00	0.00E+00			
Pae_AF241171cds5_	Pae_AF241171	6.79	4.31	5.61	0.00E+00	0.00E+00			
PA4293_at	PA4293	6.42	7.99	-2.96	0.00E+00	0.00E+00	gb|AE004091|AE004091:481657	Two-componen	Q9HWA7
PA4295_at	PA4295	8.67	6.61	4.18	0.00E+00	0.00E+00	gb|AE004091|AE004091:4819927-4820410 (+) /		Q9HWA5
PA4302_at	PA4302	6.38	8.81	-5.41	0.00E+00	0.00E+00	gb|AE004091|AE004091:4825922-4827188 (-) //		Q9HW98
PA4308_at	PA4308	5.79	9.2	-10.59	0.00E+00	0.00E+00	gb|AE004091|AE004091:4833373-4834864 (-) //		Q9HW92
PA4328_at	PA4328	6.06	8.72	-6.35	0.00E+00	0.00E+00	gb|AE004091|AE004091:4855962-4856877 (+) /		Q9HW73
PA4330_at	PA4330	6.49	4.88	3.06	0.00E+00	0.00E+00	gb|AE004091|AE004091:4858489-4859263 (-) //		Q9HW71
PA4336_at	PA4336	9.9	7.8	4.28	0.00E+00	0.00E+00	gb|AE004091|AE004091:4864993-4865578 (-) //		Q9HW65
PA4338_at	PA4338	8.95	7.32	3.09	0.00E+00	0.00E+00	gb|AE004091|AE004091:4866386-4867667 (-) //		Q9HW63
PA4357_r_at	PA4357	8.05	9.82	-3.42	0.00E+00	0.00E+00	gb|AE004091|AE004091:4884718-4884961 (-) //		Q9HW44
PA4359_i_at	PA4359	7.7	9.15	-2.73	0.00E+00	0.00E+00	gb|AE004091|AE004091:4887278-4887506 (-) //		Q9HW42
PA4361_at	PA4361	6.09	8.46	-5.16	0.00E+00	0.00E+00	gb|AE004091|AE004091:4889111-4890101 (-) //		Q9HW40
PA4362_at	PA4362	6.93	9.48	-5.88	0.00E+00	0.00E+00	gb|AE004091|AE004091:4890285-4891344 (+) /		Q9HW39
PA4367_at	PA4367	6.48	8.99	-5.7	0.00E+00	0.00E+00	gb|AE004091|AE004091:4894458-4896522 (+) /		Q9HW35
PA4381_at	PA4381	8.02	9.03	-2.02	0.00E+00	0.00E+00	gb|AE004091|AE004091:491214	Two-componen	Q9HW21
PA4389_at	PA4389	9.17	7.17	4.01	0.00E+00	0.00E+00	gb|AE004091|AE004091:4919041-4919800 (+) /		Q9HW15
PA4400_at	PA4400	7.6	8.73	-2.19	0.00E+00	0.00E+00	gb|AE004091|AE004091:4930747-4931695 (-) //		Q9HW06
PA4401_at	PA4401	6.95	8.23	-2.43	0.00E+00	0.00E+00	gb|AE004091|AE004091:4931745-4932381 (-) //		Q9HW05
PA4633_at	PA4633	7.63	8.92	-2.44	0.00E+00	0.00E+00	gb|AE004091|AE004091:520048	Chemotactic tr	Q9HVF8
PA4197_at	PA4197	6.58	8.01	-2.69	0.00E+00	0.00E+00	gb|AE004091|AE004091:469375	Two-componen	Q9HWI4
PA4650_at	PA4650	7.63	8.88	-2.37	0.00E+00	0.00E+00	gb|AE004091|AE004091:5216202-5216745 (+) /		Q9HVE2
PA4656_at	PA4656	6.83	8.38	-2.94	0.00E+00	0.00E+00	gb|AE004091|AE004091:5223558-5224476 (-) //		Q9HVD6
PA4886_at	PA4886	6.28	7.99	-3.26	0.00E+00	0.00E+00	gb|AE004091|AE004091:548106	Two-componen	Q9HUS7
PA4906_at	PA4906	5.47	8.34	-7.29	0.00E+00	0.00E+00	gb|AE004091|AE004091:5505070-5505784 (-) //		Q9HUQ7
PA4911_at	PA4911	6.22	7.95	-3.33	0.00E+00	0.00E+00	gb|AE004091|AE004091:5509550-5510828 (-) //		Q9HUQ2
PA4914_at	PA4914	8.26	7.1	2.24	0.00E+00	0.00E+00	gb|AE004091|AE004091:5513731-5514670 (+) /		Q9HUP9
PA4916_at	PA4916	6.2	7.47	-2.41	0.00E+00	0.00E+00	gb|AE004091|AE004091:5516398-5517094 (-) //		Q9HUP7
PA4917_at	PA4917	9.76	8.54	2.34	0.00E+00	0.00E+00	gb|AE004091|AE004091:5517108-5517711 (-) //		Q9HUP6
PA4918_at	PA4918	7.32	8.63	-2.49	0.00E+00	0.00E+00	gb|AE004091|AE004091:5517820-5518480 (+) /		Q9HUP5
PA4960_at	PA4960	6.37	4.57	3.47	0.00E+00	0.00E+00	gb|AE004091|AE004091:556765	Glycine, serine	Q9HUK5
PA4969_at	PA4969	6.88	8.89	-4.02	0.00E+00	0.00E+00	gb|AE004091|AE004091:5578680-5579499 (-) //		Q9HUJ6
PA4983_at	PA4983	7.51	5.35	4.48	0.00E+00	0.00E+00	gb|AE004091|AE004091:559830	Two-componen	Q9HUI2
Pae_M98270cds2_at	Pae_M98270cc	8.6	7.33	2.41	0.00E+00	0.00E+00			
Pae_L06157cds2_at	Pae_L06157cds	6.18	4.12	4.18	0.00E+00	0.00E+00			

PA4884_at	PA4884	5.87	8.43	-5.89	0.00E+00	0.00E+00	gb|AE004091|AE004091:5479640-5480264 (-) // Q9HUS9
PA4984_at	PA4984	7.47	8.73	-2.4	0.00E+00	0.00E+00	gb|AE004091|AE004091:5599159-5599807 (-) // Q9HUI1
PA4990_at	PA4990	8.48	4.23	19.01	0.00E+00	0.00E+00	gb|AE004091|AE004091:5606102-5606435 (+) / Q9HUH5
PA4995_at	PA4995	7.67	8.84	-2.26	0.00E+00	0.00E+00	gb|AE004091|AE004091:5610850-5612140 (-) // Q9HUH0
Pae_U85514cds_at	Pae_U85514cd	8.55	5.92	6.18	0.00E+00	0.00E+00	
PA5022_at	PA5022	9.34	7.8	2.9	0.00E+00	0.00E+00	gb|AE004091|AE004091:5649521-5652878 (+) / Q9HUE7
PA5024_at	PA5024	6.3	7.6	-2.47	0.00E+00	0.00E+00	gb|AE004091|AE004091:5654810-5655527 (+) // 100.0 //
PA5037_at	PA5037	5.9	8.15	-4.78	0.00E+00	0.00E+00	gb|AE004091|AE004091:5672365-5674021 (-) // Q9HUD4
PA5048_at	PA5048	7.88	9.14	-2.4	0.00E+00	0.00E+00	gb|AE004091|AE004091:5686277-5687045 (-) // Q9HUD1
PA5059_at	PA5059	4.97	7.24	-4.8	0.00E+00	0.00E+00	gb|AE004091|AE004091:5700096-5700714 (+) // 100.0 //
PA5061_at	PA5061	9.91	8.85	2.08	0.00E+00	0.00E+00	gb|AE004091|AE004091:5701697-5702114 (-) // Q9HUC2
PA5074_at	PA5074	6.69	7.93	-2.38	0.00E+00	0.00E+00	gb|AE004091|AE004091:5711476-5712211 (-) // Q9HUA9
PA5075_at	PA5075	8.98	7.68	2.47	0.00E+00	0.00E+00	gb|AE004091|AE004091:5712203-5713166 (-) // Q9HUA8
PA5085_at	PA5085	6.42	8.91	-5.59	0.00E+00	0.00E+00	gb|AE004091|AE004091:5723581-5724538 (+) / Q9HU98
PA4986_at	PA4986	7.84	5.63	4.61	0.00E+00	0.00E+00	gb|AE004091|AE004091:5601148-5603095 (+) / Q9HUH9
PA4883_at	PA4883	7.2	8.52	-2.49	0.00E+00	0.00E+00	gb|AE004091|AE004091:5478972-5479587 (-) // Q9HUT0
PA4877_at	PA4877	8.48	9.63	-2.22	0.00E+00	0.00E+00	gb|AE004091|AE004091:5473211-5473619 (-) // Q9HUT6
PA4866_at	PA4866	7.66	8.7	-2.05	0.00E+00	0.00E+00	gb|AE004091|AE004091:5463917-5464436 (+) / Q9HUU7
PA4677_at	PA4677	6.78	9.28	-5.68	0.00E+00	0.00E+00	gb|AE004091|AE004091:5246184-5247423 (-) // Q9HVB8
PA4680_at	PA4680	7.74	9.14	-2.64	0.00E+00	0.00E+00	gb|AE004091|AE004091:5249031-5249568 (-) // Q9HVB5
PA4683_at	PA4683	7.9	9.09	-2.27	0.00E+00	0.00E+00	gb|AE004091|AE004091:5251870-5252473 (-) // Q9HVB2
PA4689_at	PA4689	5.67	8.08	-5.34	0.00E+00	0.00E+00	gb|AE004091|AE004091:5260342-5262649 (-) // Q9HVA6
PA4692_at	PA4692	6.47	8.23	-3.4	0.00E+00	0.00E+00	gb|AE004091|AE004091:5270410-5271424 (-) // Q9HVA4
PA4711_at	PA4711	6.63	8.29	-3.16	0.00E+00	0.00E+00	gb|AE004091|AE004091:5291613-5291961 (+) / Q9HV87
PA4724_at	PA4724	6.55	8.72	-4.51	0.00E+00	0.00E+00	gb|AE004091|AE004091:5302899-5303781 (+) / Q9HV75
Pae_AF035937cds4_	Pae_AF035937	7.63	6.6	2.03	0.00E+00	0.00E+00	
Pae_AF035937cds3_	Pae_AF035937	5.56	3.7	3.65	0.00E+00	0.00E+00	
Pae_L06160cds_at	Pae_L06160cds	8.07	3.96	17.28	0.00E+00	0.00E+00	
Pae_M29695cds1_at	Pae_M29695cc	5.67	8.39	-6.58	0.00E+00	0.00E+00	
PA4772_at	PA4772	7.22	8.7	-2.79	0.00E+00	0.00E+00	gb|AE004091|AE004091:5358441-5361258 (+) / Q9HV36
PA4779_at	PA4779	6.52	5.31	2.32	0.00E+00	0.00E+00	gb|AE004091|AE004091:5367186-5368080 (-) // Q9HV29
PA4787_at	PA4787	6.64	8.37	-3.33	0.00E+00	0.00E+00	gb|AE004091|AE004091:5375589-5376591 (-) // Q9HV21
PA4790_at	PA4790	7.05	9.17	-4.34	0.00E+00	0.00E+00	gb|AE004091|AE004091:5378092-5378842 (+) / Q9HV18
PA4791_at	PA4791	11.08	9.95	2.2	0.00E+00	0.00E+00	gb|AE004091|AE004091:5378907-5379528 (+) / Q9HV17
PA4792_at	PA4792	8.78	7.61	2.25	0.00E+00	0.00E+00	gb|AE004091|AE004091:5379512-5380448 (-) // Q9HV16
Pae_M98270cds3_at	Pae_M98270cc	4.09	7.93	-14.34	0.00E+00	0.00E+00	
PA4794_at	PA4794	6.35	7.57	-2.33	0.00E+00	0.00E+00	gb|AE004091|AE004091:5381144-5381627 (+) / Q9HV14
PA4801_at	PA4801	5.14	8.08	-7.68	0.00E+00	0.00E+00	gb|AE004091|AE004091:5386368-5386737 (-) // Q9HV08

PA4806_at	PA4806	7.12	8.41	-2.45	0.00E+00	0.00E+00	gb|AE004091|AE004091:5391835-5392519 (+) /, Q9HV03
PA4827_at	PA4827	6.71	7.94	-2.34	0.00E+00	0.00E+00	gb|AE004091|AE004091:5418898-5419738 (-) // Q9HUY3
PA4844_at	PA4844	9.22	8.21	2.02	0.00E+00	0.00E+00	gb|AE004091|AE004091:543778· Chemotactic tr Q9HUW6
PA4852_at	PA4852	10.14	8.84	2.46	0.00E+00	0.00E+00	gb|AE004091|AE004091:5447647-5448646 (+) /, Q9HUW1
PA4857_at	PA4857	7.78	8.88	-2.15	0.00E+00	0.00E+00	gb|AE004091|AE004091:5455433-5456027 (+) /, Q9HUV6
PA4858_at	PA4858	6.56	8.88	-4.98	0.00E+00	0.00E+00	gb|AE004091|AE004091:5456451-5457717 (+) /, Q9HUV5
PA4862_at	PA4862	6.89	8.43	-2.91	0.00E+00	0.00E+00	gb|AE004091|AE004091:5461519-5462218 (+) /, Q9HUV1
PA4654_at	PA4654	5.71	8.51	-6.95	0.00E+00	0.00E+00	gb|AE004091|AE004091:5220886-5222260 (-) // Q9HVD8
PA4605_at	PA4605	6.31	7.99	-3.2	0.00E+00	0.00E+00	gb|AE004091|AE004091:5163535-5163739 (-) // Q9HVI4
Pae_flgL_at	Pae_flgL	6.05	4.9	2.21	0.00E+00	0.00E+00	
PA3288_at	PA3288	7.17	8.93	-3.38	0.00E+00	0.00E+00	gb|AE004091|AE004091:3680464-3680968 (-) // Q9HYV5
PA1025_at	PA1025	6.28	8.28	-4	0.00E+00	0.00E+00	gb|AE004091|AE004091:1110947-1112198 (+) /, Q9I4U9
PA1027_at	PA1027	6.61	8.72	-4.31	0.00E+00	0.00E+00	gb|AE004091|AE004091:1113129-1114719 (+) /, Q9I4U7
PA1030_at	PA1030	7.75	8.98	-2.35	0.00E+00	0.00E+00	gb|AE004091|AE004091:1116635-1117391 (+) /, Q9I4U4
PA1032_at	PA1032	6.81	8.73	-3.78	0.00E+00	0.00E+00	gb|AE004091|AE004091:1119674-1122218 (-) // Q9I4U2
PA1033_at	PA1033	8.07	9.08	-2.02	0.00E+00	0.00E+00	gb|AE004091|AE004091:1122340-1122982 (-) // Q9I4U1
PA1041_at	PA1041	10.85	9.72	2.19	0.00E+00	0.00E+00	gb|AE004091|AE004091:1126969-1127602 (+) /, Q9I4T3
PA1044_at	PA1044	7.63	8.85	-2.32	0.00E+00	0.00E+00	gb|AE004091|AE004091:1128857-1129322 (-) // Q9I4T0
PA1046_at	PA1046	5.92	8.26	-5.08	0.00E+00	0.00E+00	gb|AE004091|AE004091:1131674-1133948 (+) /, Q9I4S8
PA1050_at	PA1050	6.49	8.83	-5.08	0.00E+00	0.00E+00	gb|AE004091|AE004091:1137043-1138156 (+) /, Q9I4S4
PA1054_at	PA1054	8.13	9.19	-2.09	0.00E+00	0.00E+00	gb|AE004091|AE004091:1142061-1144863 (+) /, Q9I4S0
PA1056_at	PA1056	5.98	7.7	-3.3	0.00E+00	0.00E+00	gb|AE004091|AE004091:1145197-1146697 (+) /, Q9I4R8
PA1058_at	PA1058	10.4	9.35	2.07	0.00E+00	0.00E+00	gb|AE004091|AE004091:1147202-1147472 (+) /, Q9I4R6
PA1060_at	PA1060	8.51	7.27	2.35	0.00E+00	0.00E+00	gb|AE004091|AE004091:1147928-1148834 (+) /, Q9I4R4
PA1065_at	PA1065	8.05	9.14	-2.13	0.00E+00	0.00E+00	gb|AE004091|AE004091:1151435-1151909 (+) /, Q9I4Q9
PA1023_at	PA1023	5.8	7.92	-4.37	0.00E+00	0.00E+00	gb|AE004091|AE004091:1108949-1109867 (+) /, Q9I4V1
PA1068_at	PA1068	8.23	9.25	-2.04	0.00E+00	0.00E+00	gb|AE004091|AE004091:1153637-1155548 (+) /, Q9I4Q6
PA1108_at	PA1108	7.25	9.11	-3.61	0.00E+00	0.00E+00	gb|AE004091|AE004091:1200063-1201221 (-) // Q9I4M7
PA1119_at	PA1119	8.61	9.66	-2.08	0.00E+00	0.00E+00	gb|AE004091|AE004091:1212717-1213224 (-) // Q9I4L6
PA1122_at	PA1122	8.16	9.74	-2.99	0.00E+00	0.00E+00	gb|AE004091|AE004091:1215284-1215728 (+) // 100.0 //
PA1133_at	PA1133	6.55	8.02	-2.76	0.00E+00	0.00E+00	gb|AE004091|AE004091:1225340-1225691 (-) // Q9I4K2
PA1144_at	PA1144	7.36	8.69	-2.51	0.00E+00	0.00E+00	gb|AE004091|AE004091:1234816-1236151 (-) // Q9I4J1
PA1149_at	PA1149	9.19	10.22	-2.04	0.00E+00	0.00E+00	gb|AE004091|AE004091:1242750-1243119 (-) // Q9I4I6
PA1152_at	PA1152	9.49	8.09	2.64	0.00E+00	0.00E+00	gb|AE004091|AE004091:1246444-1246789 (+) // 100.0 //
PA1157_at	PA1157	6.69	8.63	-3.85	0.00E+00	0.00E+00	gb|AE004091|AE004091:125504: Two-componer Q9I4I0
PA1158_at	PA1158	5.55	8.05	-5.66	0.00E+00	0.00E+00	gb|AE004091|AE004091:125609· Two-componer Q9I4H9
PA1168_at	PA1168	7.17	8.72	-2.94	0.00E+00	0.00E+00	gb|AE004091|AE004091:1267282-1267603 (+) /, Q9I4G9
PA1169_at	PA1169	4.95	7.24	-4.88	0.00E+00	0.00E+00	gb|AE004091|AE004091:1267680-1269738 (+) /, Q9I4G8

PA1170_at	PA1170	5.91	8.64	-6.63	0.00E+00	0.00E+00	gb|AE004091|AE004091:1269781-1270516 (-) //	Q9I4G7
PA1185_at	PA1185	6.64	8.23	-3	0.00E+00	0.00E+00	gb|AE004091|AE004091:1286990-1287620 (+) /	Q9I4F3
PA1204_at	PA1204	6.62	7.96	-2.54	0.00E+00	0.00E+00	gb|AE004091|AE004091:1303892-1304450 (+) /	Q9I4D4
PA1090_at	PA1090	6.48	8.49	-4.03	0.00E+00	0.00E+00	gb|AE004091|AE004091:1176958-1177621 (+) /	Q9I4N9
PA1210_at	PA1210	8.86	6.4	5.49	0.00E+00	0.00E+00	gb|AE004091|AE004091:1310688-1311387 (-) //	Q9I4C8
PA1011_at	PA1011	6.25	8.8	-5.86	0.00E+00	0.00E+00	gb|AE004091|AE004091:1094147-1095338 (+) /	Q9I4W2
PA1006_at	PA1006	6.12	8.83	-6.54	0.00E+00	0.00E+00	gb|AE004091|AE004091:1090606-1090858 (+) /	Q9I4W7
PA0811_at	PA0811	6.24	8.6	-5.13	0.00E+00	0.00E+00	gb|AE004091|AE004091:888315-889563 (-) // 1(	Q9I5C8
PA0824_at	PA0824	7.08	8.85	-3.43	0.00E+00	0.00E+00	gb|AE004091|AE004091:899138-899657 (-) // 1(	Q9I5B5
PA0827_at	PA0827	4.76	7.7	-7.68	0.00E+00	0.00E+00	gb|AE004091|AE004091:901934-902813 (-) // 1(	Q9I5B2
PA0828_at	PA0828	9.61	7.8	3.51	0.00E+00	0.00E+00	gb|AE004091|AE004091:902900-903584 (+) // 1	Q9I5B1
PA0840_at	PA0840	5.85	8.82	-7.8	0.00E+00	0.00E+00	gb|AE004091|AE004091:915043-916156 (+) // 1	Q9I5A0
PA0841_at	PA0841	6.7	8.89	-4.58	0.00E+00	0.00E+00	gb|AE004091|AE004091:916346-917306 (+) // 1	Q9I599
PA0845_at	PA0845	7.33	8.76	-2.68	0.00E+00	0.00E+00	gb|AE004091|AE004091:921792-923805 (-) // 1(	Q9I596
PA0847_at	PA0847	6.69	8.53	-3.6	0.00E+00	0.00E+00	gb|AE004091|AE004091:925007-927215 (-) // 1(	Q9I594
PA0848_at	PA0848	6.74	8.42	-3.19	0.00E+00	0.00E+00	gb|AE004091|AE004091:927147-927747 (+) // 1	Q9I593
PA0850_at	PA0850	5.32	8.03	-6.51	0.00E+00	0.00E+00	gb|AE004091|AE004091:929084-929504 (+) // 1	Q9I591
PA0853_at	PA0853	7.15	8.91	-3.39	0.00E+00	0.00E+00	gb|AE004091|AE004091:932102-932726 (-) // 1(	Q9I588
PA0863_at	PA0863	5.99	7.93	-3.82	0.00E+00	0.00E+00	gb|AE004091|AE004091:943482-944442 (-) // 1(	Q9I578
PA0867_at	PA0867	6.27	8.1	-3.54	0.00E+00	0.00E+00	gb|AE004091|AE004091:948776-949160 (+) // 1	Q9I574
PA0875_at	PA0875	6	8.48	-5.57	0.00E+00	0.00E+00	gb|AE004091|AE004091:955991-958187 (-) // 1(	Q9I570
PA1009_at	PA1009	8.38	9.48	-2.14	0.00E+00	0.00E+00	gb|AE004091|AE004091:1092510-1093068 (-) //	Q9I4W4
PA0879_at	PA0879	5.64	8.86	-9.3	0.00E+00	0.00E+00	gb|AE004091|AE004091:961370-962531 (+) // 1	Q9I566
PA0885_at	PA0885	5.72	7.13	-2.67	0.00E+00	0.00E+00	gb|AE004091|AE004091:967570-968209 (+) // 1	Q9I560
PA0886_at	PA0886	7.2	8.98	-3.43	0.00E+00	0.00E+00	gb|AE004091|AE004091:968205-969489 (+) // 1	Q9I559
PA0891_at	PA0891	7.74	9.38	-3.11	0.00E+00	0.00E+00	gb|AE004091|AE004091:974482-975595 (+) // 1	O50184; G3XCZ4
PA0915_at	PA0915	9.4	8.37	2.04	0.00E+00	0.00E+00	gb|AE004091|AE004091:997921-998383 (-) // 1(	Q9I542
PA0924_at	PA0924	10.19	9.06	2.18	0.00E+00	0.00E+00	gb|AE004091|AE004091:1009006-1010935 (-) //	Q9I533
PA0947_at	PA0947	7.92	6.26	3.17	0.00E+00	0.00E+00	gb|AE004091|AE004091:1035277-1035982 (+) /	Q9I511
PA0955_at	PA0955	5.47	7.14	-3.17	0.00E+00	0.00E+00	gb|AE004091|AE004091:1040724-1041681 (-) //	Q9I503
PA0957_at	PA0957	8.13	6.01	4.34	0.00E+00	0.00E+00	gb|AE004091|AE004091:1043512-1043920 (+) /	Q9I501
PA0960_at	PA0960	4.52	9.14	-24.67	0.00E+00	0.00E+00	gb|AE004091|AE004091:1046462-1046672 (+) /	Q9I4Z9
PA0961_at	PA0961	8.17	9.64	-2.78	0.00E+00	0.00E+00	gb|AE004091|AE004091:1046720-1046912 (-) // 100.0 //	
PA0976_at	PA0976	6.86	8.61	-3.38	0.00E+00	0.00E+00	gb|AE004091|AE004091:1059622-1060297 (+) /	Q9I4Z2
PA0977_at	PA0977	9.42	8.3	2.17	0.00E+00	0.00E+00	gb|AE004091|AE004091:1060510-1060834 (-) // 100.0 //	
PA0978_s_at	PA0978	7.85	9.08	-2.34	0.00E+00	0.00E+00	gb|AE004091|AE004091:2118918-2119746 (+) /	Q9I4Z0; Q9I2G6
PA0981_at	PA0981	6.06	7.16	-2.14	0.00E+00	0.00E+00	gb|AE004091|AE004091:1062921-1063545 (+) /	Q9I4Y8
PA0882_at	PA0882	8.97	5.8	9	0.00E+00	0.00E+00	gb|AE004091|AE004091:964372-965575 (+) // 1	Q9I563

PA0807_at	PA0807	8.15	9.76	-3.06	0.00E+00	0.00E+00	gb|AE004091|AE004091:884799-885567 (+) // 1	Q9I5D1
PA1211_at	PA1211	9.2	7.88	2.5	0.00E+00	0.00E+00	gb|AE004091|AE004091:1311504-1312140 (-) //	Q9I4C7
PA1223_at	PA1223	6.07	8	-3.81	0.00E+00	0.00E+00	gb|AE004091|AE004091:1326046-1326940 (-) //	Q9I4B5
PA1417_at	PA1417	7.86	9.36	-2.82	0.00E+00	0.00E+00	gb|AE004091|AE004091:1541145-1542747 (-) //	Q9I3S7
PA1423_at	PA1423	8.88	7.79	2.13	0.00E+00	0.00E+00	gb|AE004091|AE004091:154833 Chemotactic tr	Q9I3S1
PA1428_at	PA1428	5.54	8.28	-6.72	0.00E+00	0.00E+00	gb|AE004091|AE004091:1554415-1554847 (+) /	Q9I3R6
PA1439_at	PA1439	6.26	7.84	-2.99	0.00E+00	0.00E+00	gb|AE004091|AE004091:1569302-1569710 (-) //	Q9I3Q8
PA1450_at	PA1450	6.93	8.21	-2.43	0.00E+00	0.00E+00	gb|AE004091|AE004091:1577547-1578807 (+) /	Q9I3Q1
PA1467_at	PA1467	10.25	8.96	2.44	0.00E+00	0.00E+00	gb|AE004091|AE004091:1595827-1596799 (+) /	Q9I3P5
PA1486_at	PA1486	8.73	7.48	2.36	0.00E+00	0.00E+00	gb|AE004091|AE004091:1612126-1613227 (+) /	Q9I3M6
PA1490_at	PA1490	7.17	8.53	-2.57	0.00E+00	0.00E+00	gb|AE004091|AE004091:1617489-1618266 (-) //	Q9I3M2
PA1496_at	PA1496	6.57	8.15	-3	0.00E+00	0.00E+00	gb|AE004091|AE004091:1623864-1624716 (-) //	Q9I3L6
PA1497_at	PA1497	5.45	8.44	-7.96	0.00E+00	0.00E+00	gb|AE004091|AE004091:1624781-1625696 (-) //	Q9I3L5
PA1501_at	PA1501	9	7.68	2.5	0.00E+00	0.00E+00	gb|AE004091|AE004091:1630024-1630807 (-) //	Q9I3L1
PA1503_at	PA1503	6.18	8.03	-3.62	0.00E+00	0.00E+00	gb|AE004091|AE004091:1633006-1633438 (+) /	Q9I3K9
PA1506_at	PA1506	9.21	7.62	3	0.00E+00	0.00E+00	gb|AE004091|AE004091:1635908-1636238 (+) /	Q9I3K6
PA1519_at	PA1519	5.54	8.87	-10.06	0.00E+00	0.00E+00	gb|AE004091|AE004091:1650549-1651899 (-) //	Q9I3J4
PA1412_at	PA1412	9.06	7.74	2.5	0.00E+00	0.00E+00	gb|AE004091|AE004091:1536457-1537651 (-) //	Q9I3T2
PA1521_at	PA1521	7.38	8.92	-2.9	0.00E+00	0.00E+00	gb|AE004091|AE004091:165310 Purine metabo	Q9I3J2
PA1552_at	PA1552	6.04	8.64	-6.05	0.00E+00	0.00E+00	gb|AE004091|AE004091:1689557-1690514 (-) //	Q9I3G5
PA1559_at	PA1559	9.83	8.01	3.52	0.00E+00	0.00E+00	gb|AE004091|AE004091:1697188-1697920 (+) // 100.0 //	
PA1565_at	PA1565	7.61	6.5	2.16	0.00E+00	0.00E+00	gb|AE004091|AE004091:1704793-1706104 (-) //	Q9I3F2
PA1566_at	PA1566	5.52	7.53	-4.04	0.00E+00	0.00E+00	gb|AE004091|AE004091:1706172-1707537 (-) //	Q9I3F1
PA1576_at	PA1576	8.16	6.26	3.75	0.00E+00	0.00E+00	gb|AE004091|AE004091:1715809-1716676 (+) /	Q9I3E1
PA1579_at	PA1579	7.45	9.18	-3.31	0.00E+00	0.00E+00	gb|AE004091|AE004091:1718386-1718995 (+) /	Q9I3D8
PA1595_at	PA1595	6.76	7.91	-2.22	0.00E+00	0.00E+00	gb|AE004091|AE004091:1736189-1737419 (+) /	Q9I3C6
PA1598_at	PA1598	7.84	9.2	-2.57	0.00E+00	0.00E+00	gb|AE004091|AE004091:1740244-1741066 (-) //	Q9I3C3
PA1601_at	PA1601	5.89	7.85	-3.88	0.00E+00	0.00E+00	gb|AE004091|AE004091:1743290-1745537 (-) //	Q9I3C0
PA1606_at	PA1606	10.31	9.3	2.02	0.00E+00	0.00E+00	gb|AE004091|AE004091:1748699-1749176 (+) /	Q9I3B5
PA1608_at	PA1608	7.93	9.31	-2.6	0.00E+00	0.00E+00	gb|AE004091|AE004091:174989 Chemotactic tr	Q9I3B3
PA1620_at	PA1620	6.88	9.12	-4.72	0.00E+00	0.00E+00	gb|AE004091|AE004091:1763793-1764435 (+) /	Q9I3A2
PA1625_at	PA1625	8.07	9.55	-2.79	0.00E+00	0.00E+00	gb|AE004091|AE004091:1767928-1768996 (+) /	Q9I397
PA1626_at	PA1626	9.77	8.23	2.9	0.00E+00	0.00E+00	gb|AE004091|AE004091:1769047-1770322 (-) //	Q9I396
PA1531_at	PA1531	4.89	6.52	-3.1	0.00E+00	0.00E+00	gb|AE004091|AE004091:1669086-1669953 (-) //	Q9I3I2
PA1222_at	PA1222	7.41	9.1	-3.22	0.00E+00	0.00E+00	gb|AE004091|AE004091:1324793-1325951 (-) //	Q9I4B6
PA1395_at	PA1395	6.23	9.56	-10.05	0.00E+00	0.00E+00	gb|AE004091|AE004091:1516703-1517120 (+) /	Q9I3U9
PA1379_at	PA1379	4.91	7.25	-5.07	0.00E+00	0.00E+00	gb|AE004091|AE004091:1496087-1496921 (-) //	Q9I3W5
PA1224_at	PA1224	7.2	9.17	-3.92	0.00E+00	0.00E+00	gb|AE004091|AE004091:1327024-1327804 (+) /	Q9I4B4

PA1225_at	PA1225	5.71	8.44	-6.65	0.00E+00	0.00E+00	gb|AE004091|AE004091:1327813-1328440 (-) // Q9I4B3
PA1229_at	PA1229	6.78	9.34	-5.9	0.00E+00	0.00E+00	gb|AE004091|AE004091:1330577-1331384 (-) // Q9I4A9
PA1231_at	PA1231	8.9	7.8	2.14	0.00E+00	0.00E+00	gb|AE004091|AE004091:1331727-1332636 (+) /, Q9I4A7
PA1254_at	PA1254	6.24	8.11	-3.67	0.00E+00	0.00E+00	gb|AE004091|AE004091:1362371-1363289 (-) // Q9I490
PA1262_at	PA1262	9.45	8.22	2.35	0.00E+00	0.00E+00	gb|AE004091|AE004091:1370257-1371700 (-) // Q9I482
PA1270_at	PA1270	9.04	7.87	2.24	0.00E+00	0.00E+00	gb|AE004091|AE004091:1379413-1381453 (+) /, Q9I474
PA1271_at	PA1271	7.87	9.02	-2.22	0.00E+00	0.00E+00	gb|AE004091|AE004091:1381804-1383655 (+) /, Q9I473
PA1282_at	PA1282	6.26	8.2	-3.82	0.00E+00	0.00E+00	gb|AE004091|AE004091:1392564-1394070 (-) // Q9I462
PA1284_at	PA1284	7.15	8.37	-2.32	0.00E+00	0.00E+00	gb|AE004091|AE004091:1394779-1396600 (-) // Q9I460
PA1287_at	PA1287	7.09	9.33	-4.71	0.00E+00	0.00E+00	gb|AE004091|AE004091:1398618-1399173 (+) /, Q9I457
PA1288_at	PA1288	7.69	9.84	-4.42	0.00E+00	0.00E+00	gb|AE004091|AE004091:1399231-1400506 (-) // Q9I456
PA1290_at	PA1290	6.56	9.12	-5.9	0.00E+00	0.00E+00	gb|AE004091|AE004091:1401412-1402006 (-) // Q9I454
PA1292_at	PA1292	6.52	9.21	-6.48	0.00E+00	0.00E+00	gb|AE004091|AE004091:1402981-1403836 (-) // Q9I452
PA1386_at	PA1386	7.43	8.82	-2.63	0.00E+00	0.00E+00	gb|AE004091|AE004091:1505749-1507018 (+) /, Q9I3V8
PA1297_at	PA1297	6.58	8.3	-3.28	0.00E+00	0.00E+00	gb|AE004091|AE004091:1407951-1408932 (-) // Q9I447
PA1303_at	PA1303	5.84	8.12	-4.88	0.00E+00	0.00E+00	gb|AE004091|AE004091:1414147-1414687 (-) // Q9I441
PA1306_at	PA1306	6.56	8.07	-2.84	0.00E+00	0.00E+00	gb|AE004091|AE004091:1417500-1417962 (+) /, Q9I438
PA1312_at	PA1312	6.46	8.38	-3.79	0.00E+00	0.00E+00	gb|AE004091|AE004091:1422355-1423267 (-) // Q9I432
PA1316_at	PA1316	7.1	9.01	-3.75	0.00E+00	0.00E+00	gb|AE004091|AE004091:1425912-1427454 (+) /, Q9I428
PA1325_at	PA1325	7.35	8.89	-2.91	0.00E+00	0.00E+00	gb|AE004091|AE004091:1436663-1437071 (+) /, Q9I419
PA1328_at	PA1328	4.81	6.37	-2.95	0.00E+00	0.00E+00	gb|AE004091|AE004091:1440639-1441548 (-) // Q9I416
PA1344_at	PA1344	6.77	8.35	-2.99	0.00E+00	0.00E+00	gb|AE004091|AE004091:1457729-1458524 (+) /, Q9I400
PA1346_at	PA1346	8.11	9.34	-2.35	0.00E+00	0.00E+00	gb|AE004091|AE004091:1460307-1461855 (-) // Q9I3Z8
PA1355_at	PA1355	9.8	8.6	2.3	0.00E+00	0.00E+00	gb|AE004091|AE004091:1468510-1468891 (+) /, Q9I3Y9
PA1360_at	PA1360	9.05	6.64	5.31	0.00E+00	0.00E+00	gb|AE004091|AE004091:1471671-1472565 (+) /, Q9I3Y4
PA1363_at	PA1363	6.59	8.43	-3.57	0.00E+00	0.00E+00	gb|AE004091|AE004091:1474727-1475468 (+) /, Q9I3Y1
PA1370_at	PA1370	8.13	6.84	2.44	0.00E+00	0.00E+00	gb|AE004091|AE004091:1483898-1485764 (+) /, Q9I3X4
PA1372_at	PA1372	8.12	7.05	2.1	0.00E+00	0.00E+00	gb|AE004091|AE004091:1486960-1489096 (-) // Q9I3X2
PA1374_at	PA1374	7.47	9.39	-3.78	0.00E+00	0.00E+00	gb|AE004091|AE004091:1491192-1491699 (+) /, Q9I3X0
PA1300_at	PA1300	6.22	8.7	-5.6	0.00E+00	0.00E+00	gb|AE004091|AE004091:1409949-1410477 (+) /, Q9I444
PA0803_at	PA0803	4.83	6.27	-2.72	0.00E+00	0.00E+00	gb|AE004091|AE004091:881486-881927 (+) // 1 Q9I5D5
PA0802_i_at	PA0802	6.75	8.22	-2.78	0.00E+00	0.00E+00	gb|AE004091|AE004091:881077-881401 (+) // 1 Q9I5D6
PA0799_at	PA0799	5.56	7.1	-2.91	0.00E+00	0.00E+00	gb|AE004091|AE004091:876617-878609 (-) // 1(Q9I5D9
PA0193_at	PA0193	6.92	9.21	-4.89	0.00E+00	0.00E+00	gb|AE004091|AE004091:221585-222488 (+) // 1 Q9I6U1
PA0203_at	PA0203	7.65	8.73	-2.12	0.00E+00	0.00E+00	gb|AE004091|AE004091:232066-233101 (-) // 1(Q9I6T5
PA0204_at	PA0204	6.64	8.35	-3.28	0.00E+00	0.00E+00	gb|AE004091|AE004091:233123-233933 (-) // 1(Q9I6T4
PA0207_at	PA0207	10.61	8.79	3.54	0.00E+00	0.00E+00	gb|AE004091|AE004091:236218-237112 (+) // 1 Q9I6T1
PA0220_at	PA0220	6.41	8.93	-5.77	0.00E+00	0.00E+00	gb|AE004091|AE004091:248767-250201 (+) // 1 Q9I6R8

PA0222_at	PA0222	7.12	5.02	4.27	0.00E+00	0.00E+00	gb|AE004091|AE004091:251777-252836 (+) // 1	Q9I6R6
PA0223_at	PA0223	9.33	6.71	6.13	0.00E+00	0.00E+00	gb|AE004091|AE004091:252913-253795 (-) // 1(	Q9I6R5
PA0225_at	PA0225	6.66	8.93	-4.81	0.00E+00	0.00E+00	gb|AE004091|AE004091:254791-255331 (+) // 1	Q9I6R3
PA0226_at	PA0226	7.43	9.33	-3.74	0.00E+00	0.00E+00	gb|AE004091|AE004091:255481-256333 (+) // 1	Q9I6R2
PA0233_at	PA0233	5.49	7.45	-3.9	0.00E+00	0.00E+00	gb|AE004091|AE004091:262557-263499 (+) // 1	Q9I6Q5
PA0234_at	PA0234	7.74	8.96	-2.33	0.00E+00	0.00E+00	gb|AE004091|AE004091:263689-264523 (+) // 1	Q9I6Q4
PA0236_at	PA0236	10.17	8.93	2.35	0.00E+00	0.00E+00	gb|AE004091|AE004091:266616-267396 (+) // 1	Q9I6Q2
PA0239_at	PA0239	7.99	9.36	-2.59	0.00E+00	0.00E+00	gb|AE004091|AE004091:269669-270548 (+) // 1	Q9I6P9
PA0243_at	PA0243	6.54	7.71	-2.26	0.00E+00	0.00E+00	gb|AE004091|AE004091:275772-276441 (+) // 1	Q9I6P5
PA0188_at	PA0188	9.88	8.76	2.16	0.00E+00	0.00E+00	gb|AE004091|AE004091:214631-215513 (+) // 1	Q9I6U6
PA0249_at	PA0249	7.56	8.77	-2.32	0.00E+00	0.00E+00	gb|AE004091|AE004091:281799-282246 (-) // 1(	Q9I6P0
PA0256_at	PA0256	8.19	9.48	-2.46	0.00E+00	0.00E+00	gb|AE004091|AE004091:287188-288121 (-) // 1(	Q9I6N3
PA0272_at	PA0272	7.3	8.6	-2.47	0.00E+00	0.00E+00	gb|AE004091|AE004091:306896-307829 (-) // 1(	Q9I6L8
PA0277_at	PA0277	6.03	8.67	-6.23	0.00E+00	0.00E+00	gb|AE004091|AE004091:311451-312210 (-) // 1(	Q9I6L3
PA0287_at	PA0287	7.63	5.82	3.5	0.00E+00	0.00E+00	gb|AE004091|AE004091:322175-323561 (+) // 1	Q9I6K3
PA0289_at	PA0289	8.2	9.27	-2.1	0.00E+00	0.00E+00	gb|AE004091|AE004091:324625-325588 (+) // 1	Q9I6K1
PA0311_at	PA0311	7.46	9.12	-3.16	0.00E+00	0.00E+00	gb|AE004091|AE004091:351610-352165 (-) // 1(	Q9I6I0
PA0313_at	PA0313	7.85	8.87	-2.03	0.00E+00	0.00E+00	gb|AE004091|AE004091:352927-353620 (-) // 1(	Q9I6H8
PA0323_at	PA0323	7.95	9.16	-2.31	0.00E+00	0.00E+00	gb|AE004091|AE004091:363278-364322 (-) // 1(	Q9I6G8
PA0324_at	PA0324	6.66	8.98	-5	0.00E+00	0.00E+00	gb|AE004091|AE004091:364369-365158 (-) // 1(	Q9I6G7
PA0327_at	PA0327	4.88	7.78	-7.44	0.00E+00	0.00E+00	gb|AE004091|AE004091:367457-368423 (+) // 1	Q9I6G4
PA0332_at	PA0332	8.32	9.39	-2.1	0.00E+00	0.00E+00	gb|AE004091|AE004091:373725-374193 (+) // 1	Q9I6F9
PA0338_at	PA0338	6.29	7.67	-2.61	0.00E+00	0.00E+00	gb|AE004091|AE004091:380903-382034 (+) // 1	Q9I6F5
PA0339_at	PA0339	9.2	8.15	2.06	0.00E+00	0.00E+00	gb|AE004091|AE004091:382037-382793 (-) // 1(	Q9I6F4
PA0344_at	PA0344	9.64	8.36	2.44	0.00E+00	0.00E+00	gb|AE004091|AE004091:386386-387766 (-) // 1(	Q9I6E9
PA0250_at	PA0250	9.3	5.32	15.8	0.00E+00	0.00E+00	gb|AE004091|AE004091:282323-282758 (-) // 1(	Q9I6N9
PA0356_at	PA0356	6.06	7.41	-2.54	0.00E+00	0.00E+00	gb|AE004091|AE004091:400248-401073 (+) // 1	Q9I6D7
PA0184_at	PA0184	6.62	8.51	-3.7	0.00E+00	0.00E+00	gb|AE004091|AE004091:209621-210461 (-) // 1(	Q9I6U9
PA0180_at	PA0180	9.73	8.47	2.41	0.00E+00	0.00E+00	gb|AE004091|AE004091:204657- Chemotactic tr	Q9I6V2
Pae_orfA_vioA_at	Pae_orfA_vioA	9.07	6.82	4.77	0.00E+00	0.00E+00		
Pae_orfC_at	Pae_orfC	5.82	4.48	2.53	0.00E+00	0.00E+00		
Pae_orfF_at	Pae_orfF	6.09	8.25	-4.47	0.00E+00	0.00E+00		
Pae_orfK_at	Pae_orfK	4.57	6.02	-2.73	0.00E+00	0.00E+00		
PA0006_at	PA0006	7.28	5.95	2.5	0.00E+00	0.00E+00	gb|AE004091|AE004091:7803-8340 (-) // 100.0 ,	Q9I7C0
PA0007_at	PA0007	5.88	8.88	-8	0.00E+00	0.00E+00	gb|AE004091|AE004091:8671-10378 (+) // 100.(	Q9I7B9
PA0012_at	PA0012	6.7	8.65	-3.88	0.00E+00	0.00E+00	gb|AE004091|AE004091:15207-15474 (+) // 100	Q9I7B4
PA0027_at	PA0027	6.78	8.16	-2.61	0.00E+00	0.00E+00	gb|AE004091|AE004091:28613-29900 (+) // 100	Q9I7A3
PA0047_at	PA0047	5.69	8.52	-7.12	0.00E+00	0.00E+00	gb|AE004091|AE004091:61879-62389 (+) // 100	Q9I785

PA0056_at	PA0056	9.13	7.82	2.47	0.00E+00	0.00E+00	gb|AE004091|AE004091:70702-71623 (-) // 100.	Q9I776
PA0058_at	PA0058	6.37	8.62	-4.74	0.00E+00	0.00E+00	gb|AE004091|AE004091:72680-73385 (+) // 100	Q9I774
PA0064_at	PA0064	7.71	8.79	-2.1	0.00E+00	0.00E+00	gb|AE004091|AE004091:76416-77400 (-) // 100.	Q9I768
PA0069_at	PA0069	8.93	7.65	2.44	0.00E+00	0.00E+00	gb|AE004091|AE004091:81116-82175 (+) // 100	Q9I763
PA0071_at	PA0071	9.81	8.39	2.68	0.00E+00	0.00E+00	gb|AE004091|AE004091:83380-85093 (-) // 100.	Q9I761
PA0182_at	PA0182	5.72	8.54	-7.03	0.00E+00	0.00E+00	gb|AE004091|AE004091:207071-207824 (+) // 1	Q9I6V0
PA0078_at	PA0078	6.22	8.94	-6.6	0.00E+00	0.00E+00	gb|AE004091|AE004091:95048-96398 (-) // 100.	Q9I754
PA0089_at	PA0089	6.36	7.43	-2.09	0.00E+00	0.00E+00	gb|AE004091|AE004091:107182-108229 (+) // 1	Q9I743
PA0093_at	PA0093	9.38	8.3	2.11	0.00E+00	0.00E+00	gb|AE004091|AE004091:113303-114596 (-) // 1(	Q9I739
PA0096_at	PA0096	9.61	8.5	2.16	0.00E+00	0.00E+00	gb|AE004091|AE004091:117552-118002 (+) // 1	Q9I736
PA0116_at	PA0116	6.74	7.92	-2.27	0.00E+00	0.00E+00	gb|AE004091|AE004091:136518-136992 (+) // 1	Q9I716
PA0123_at	PA0123	8.2	9.21	-2.01	0.00E+00	0.00E+00	gb|AE004091|AE004091:142359-143268 (-) // 1(	Q9I709
PA0131_at	PA0131	6.97	8.45	-2.78	0.00E+00	0.00E+00	gb|AE004091|AE004091:149138-149426 (-) // 1(	Q9I701
PA0134_at	PA0134	8.1	9.27	-2.25	0.00E+00	0.00E+00	gb|AE004091|AE004091:151936- Purine metabo	Q9I6Z8
PA0135_at	PA0135	5.84	8.21	-5.18	0.00E+00	0.00E+00	gb|AE004091|AE004091:153696-153837 (+) // 1	Q9I6Z7
PA0144_at	PA0144	9.15	7.82	2.51	0.00E+00	0.00E+00	gb|AE004091|AE004091:164443-165070 (-) // 1(	Q9I6Y8
PA0145_i_at	PA0145	8.26	6.24	4.07	0.00E+00	0.00E+00	gb|AE004091|AE004091:165219-165738 (-) // 1(	Q9I6Y7
PA0157_at	PA0157	5.59	8.65	-8.33	0.00E+00	0.00E+00	gb|AE004091|AE004091:178455-179526 (+) // 1	Q9I6X5
PA0158_at	PA0158	6.23	7.37	-2.2	0.00E+00	0.00E+00	gb|AE004091|AE004091:179522-182570 (+) // 1	Q9I6X4
PA0172_at	PA0172	7	8.14	-2.21	0.00E+00	0.00E+00	gb|AE004091|AE004091:194757-196749 (-) // 1(	Q9I6W0
PA0176_at	PA0176	10.06	8.6	2.75	0.00E+00	0.00E+00	gb|AE004091|AE004091:199600- Chemotactic tr	Q9I6V6
PA0082_at	PA0082	6.64	8.08	-2.71	0.00E+00	0.00E+00	gb|AE004091|AE004091:100124-101159 (+) // 1	Q9I750
PA0358_at	PA0358	8.35	9.39	-2.06	0.00E+00	0.00E+00	gb|AE004091|AE004091:402020-402599 (+) // 1	Q9I6D6
PA0361_at	PA0361	5.88	7.92	-4.11	0.00E+00	0.00E+00	gb|AE004091|AE004091:404386- Glutathione me	Q9I6D3
PA0368_at	PA0368	10.11	8.65	2.73	0.00E+00	0.00E+00	gb|AE004091|AE004091:412329-413328 (-) // 1(	Q9I6C6
PA0641_at	PA0641	9.24	8.23	2.01	0.00E+00	0.00E+00	gb|AE004091|AE004091:695082-698697 (+) // 1	Q9I5S5
PA0645_at	PA0645	7.36	8.38	-2.02	0.00E+00	0.00E+00	gb|AE004091|AE004091:701151-701382 (+) // 1	Q9I5S1
PA0646_at	PA0646	7.87	8.94	-2.1	0.00E+00	0.00E+00	gb|AE004091|AE004091:701477-702530 (+) // 1	Q9I5S0
PA0647_at	PA0647	9.46	7.43	4.08	0.00E+00	0.00E+00	gb|AE004091|AE004091:702529-702832 (+) // 1	Q9I5R9
PA0653_at	PA0653	7.74	9.05	-2.48	0.00E+00	0.00E+00	gb|AE004091|AE004091:706944-707367 (+) // 1	G3XDA4
PA0660_at	PA0660	6.61	7.96	-2.56	0.00E+00	0.00E+00	gb|AE004091|AE004091:713279-714248 (-) // 1(	Q9I5R1
PA0666_at	PA0666	6.04	7.53	-2.8	0.00E+00	0.00E+00	gb|AE004091|AE004091:717635-718727 (-) // 1(	Q9I5Q5
PA0681_at	PA0681	4.97	8.4	-10.79	0.00E+00	0.00E+00	gb|AE004091|AE004091:738719-739169 (+) // 1	Q9I5P4
PA0686_at	PA0686	5.17	8.2	-8.19	0.00E+00	0.00E+00	gb|AE004091|AE004091:744333-745743 (+) // 1	Q9I5N9
PA0687_at	PA0687	6.93	8.79	-3.63	0.00E+00	0.00E+00	gb|AE004091|AE004091:745742-746957 (+) // 1	Q9I5N8
PA0690_at	PA0690	4.64	5.68	-2.06	0.00E+00	0.00E+00	gb|AE004091|AE004091:749957-762500 (+) // 1	Q9I5N6
PA0691_at	PA0691	7.51	8.7	-2.27	0.00E+00	0.00E+00	gb|AE004091|AE004091:762891-763494 (+) // 1	Q9I5N5
PA0695_at	PA0695	8.16	6.59	2.99	0.00E+00	0.00E+00	gb|AE004091|AE004091:767601-768348 (+) // 1	Q9I5N1

PA0696_at	PA0696	5.4	7.15	-3.38	0.00E+00	0.00E+00	gb|AE004091|AE004091:768415-770122 (+) // 1	Q9I5N0
PA0640_at	PA0640	8.59	7.08	2.84	0.00E+00	0.00E+00	gb|AE004091|AE004091:694421-695024 (+) // 1	Q9I5S6
PA0697_at	PA0697	9.36	8.01	2.55	0.00E+00	0.00E+00	gb|AE004091|AE004091:770156-770819 (+) // 1	Q9I5M9
PA0704_at	PA0704	6.08	7.63	-2.93	0.00E+00	0.00E+00	gb|AE004091|AE004091:776787-778182 (-) // 1(	Q9I5M2
PA0734_i_at	PA0734	8.18	6.07	4.34	0.00E+00	0.00E+00	gb|AE004091|AE004091:801967-802240 (-) // 1(	Q9I5J5
PA0735_at	PA0735	5.31	8.07	-6.78	0.00E+00	0.00E+00	gb|AE004091|AE004091:802260-803076 (-) // 1(	Q9I5J4
PA0740_at	PA0740	7.53	8.56	-2.04	0.00E+00	0.00E+00	gb|AE004091|AE004091:806805-808782 (+) // 1	Q9I5I9
PA0741_at	PA0741	8.96	6.07	7.41	0.00E+00	0.00E+00	gb|AE004091|AE004091:808816-809458 (-) // 1(	Q9I5I8
PA0742_at	PA0742	5.62	8.5	-7.38	0.00E+00	0.00E+00	gb|AE004091|AE004091:809574-809883 (-) // 100.0 //	
PA0749_at	PA0749	7.7	9.21	-2.84	0.00E+00	0.00E+00	gb|AE004091|AE004091:817082-817904 (+) // 1	Q9I5I0
PA0760_at	PA0760	6.22	7.95	-3.31	0.00E+00	0.00E+00	gb|AE004091|AE004091:828617-828872 (+) // 1	Q9I5G9
PA0779_at	PA0779	7.72	8.8	-2.12	0.00E+00	0.00E+00	gb|AE004091|AE004091:845793-848193 (-) // 1(	Q9I5F9
PA0784_at	PA0784	7.53	8.91	-2.6	0.00E+00	0.00E+00	gb|AE004091|AE004091:856942-857872 (-) // 1(	Q9I5F4
PA0785_at	PA0785	6.12	8.23	-4.32	0.00E+00	0.00E+00	gb|AE004091|AE004091:857998-858637 (+) // 1	Q9I5F3
PA0786_at	PA0786	8.06	4.98	8.46	0.00E+00	0.00E+00	gb|AE004091|AE004091:858646-858952 (+) // 1	Q9I5F2
PA0791_at	PA0791	9.16	7.91	2.38	0.00E+00	0.00E+00	gb|AE004091|AE004091:866558-867347 (+) // 1	Q9I5E7
PA0797_at	PA0797	7.75	9.09	-2.53	0.00E+00	0.00E+00	gb|AE004091|AE004091:875113-875836 (-) // 1(	Q9I5E1
PA0700_at	PA0700	7.71	8.75	-2.06	0.00E+00	0.00E+00	gb|AE004091|AE004091:772275-772701 (+) // 1	Q9I5M6
PA0638_at	PA0638	6.08	7.74	-3.16	0.00E+00	0.00E+00	gb|AE004091|AE004091:692898-693594 (+) // 1	Q9I5S8
PA0634_at	PA0634	5.26	7.13	-3.67	0.00E+00	0.00E+00	gb|AE004091|AE004091:690043-690391 (+) // 1	Q9R317; G3XD68
PA0632_at	PA0632	6.35	8.38	-4.08	0.00E+00	0.00E+00	gb|AE004091|AE004091:689236-689467 (+) // 100.0 //	
PA0385_at	PA0385	6.6	9.45	-7.22	0.00E+00	0.00E+00	gb|AE004091|AE004091:427182-427506 (-) // 1(	Q9I6B0
PA0401_at	PA0401	7.51	8.66	-2.23	0.00E+00	0.00E+00	gb|AE004091|AE004091:443419-444691 (-) // 100.0 //	
PA0418_at	PA0418	4.62	7.64	-8.12	0.00E+00	0.00E+00	gb|AE004091|AE004091:464568-465984 (-) // 1(	Q9I695
PA0439_at	PA0439	5.82	4.36	2.73	0.00E+00	0.00E+00	gb|AE004091|AE004091:492080-493358 (-) // 1(	Q9I678
PA0442_r_at	PA0442	4.75	2.32	5.39	0.00E+00	0.00E+00	gb|AE004091|AE004091:496362-496479 (-) // 1(	Q9I675
PA0443_at	PA0443	6.54	8.69	-4.41	0.00E+00	0.00E+00	gb|AE004091|AE004091:496871-498362 (+) // 1	Q9I674
PA0446_at	PA0446	6.77	8.49	-3.29	0.00E+00	0.00E+00	gb|AE004091|AE004091:501376-502600 (-) // 1(	Q9I672
PA0453_at	PA0453	8.06	6.58	2.79	0.00E+00	0.00E+00	gb|AE004091|AE004091:509825-510500 (-) // 100.0 //	
PA0454_at	PA0454	6.74	9.9	-8.96	0.00E+00	0.00E+00	gb|AE004091|AE004091:510589-512791 (-) // 1(	Q9I664
PA0456_at	PA0456	10.15	11.37	-2.32	0.00E+00	0.00E+00	gb|AE004091|AE004091:514775-514985 (+) // 1	Q9I662
PA0471_at	PA0471	7.76	8.9	-2.21	0.00E+00	0.00E+00	gb|AE004091|AE004091:532541· Two-componei	Q9I647
PA0479_at	PA0479	6.07	8.62	-5.86	0.00E+00	0.00E+00	gb|AE004091|AE004091:539785-540736 (-) // 1(	Q9I639
PA0480_at	PA0480	6.34	7.6	-2.39	0.00E+00	0.00E+00	gb|AE004091|AE004091:540839-541637 (+) // 1	Q9I638
PA0491_at	PA0491	8.88	7.76	2.17	0.00E+00	0.00E+00	gb|AE004091|AE004091:550867-551794 (-) // 1(	Q9I627
PA0493_at	PA0493	6.27	8.8	-5.78	0.00E+00	0.00E+00	gb|AE004091|AE004091:552746-552995 (+) // 1	Q9I625
PA0496_at	PA0496	7.87	9.15	-2.43	0.00E+00	0.00E+00	gb|AE004091|AE004091:555251-556229 (+) // 1	Q9I622
PA0497_at	PA0497	9.07	8.03	2.06	0.00E+00	0.00E+00	gb|AE004091|AE004091:556275-557277 (-) // 1(	Q9I621

PA0628_at	PA0628	7.23	9.27	-4.11	0.00E+00	0.00E+00	gb|AE004091|AE004091:686957-687947 (+) // 1		Q9S567; G3XCU8
PA0618_at	PA0618	10.36	9.32	2.06	0.00E+00	0.00E+00	gb|AE004091|AE004091:677406-678294 (+) // 1		Q9S578; G3XCX5
PA0617_at	PA0617	7.25	5.67	2.98	0.00E+00	0.00E+00	gb|AE004091|AE004091:677083-677410 (+) // 1		Q9S579; G3XD42
PA0612_i_at	PA0612	8.12	9.29	-2.25	0.00E+00	0.00E+00	gb|AE004091|AE004091:674419-674620 (+) // 1		Q9R2Q7; G3XDA6
PA0597_at	PA0597	8.15	5.11	8.19	0.00E+00	0.00E+00	gb|AE004091|AE004091:657667-658342 (+) // 1		Q9I5U0
PA0581_i_at	PA0581	5.7	8.31	-6.07	0.00E+00	0.00E+00	gb|AE004091|AE004091:640420-640990 (-) // 1(		Q9I5V6
PA1627_at	PA1627	6.63	8.72	-4.26	0.00E+00	0.00E+00	gb|AE004091|AE004091:1770337-1770985 (-) //		Q9I395
PA0578_at	PA0578	6.29	8.14	-3.61	0.00E+00	0.00E+00	gb|AE004091|AE004091:638381-638831 (-) // 1(		Q9I5V9
PA0558_at	PA0558	8.32	6.57	3.37	0.00E+00	0.00E+00	gb|AE004091|AE004091:615607-616375 (+) // 1		Q9I5X8
PA0556_at	PA0556	7.13	5.76	2.58	0.00E+00	0.00E+00	gb|AE004091|AE004091:614468-614951 (+) // 1		Q9I5Y0
PA0536_at	PA0536	6.88	8.53	-3.13	0.00E+00	0.00E+00	gb|AE004091|AE004091:595245-596271 (-) // 1(		Q9I600
PA0515_at	PA0515	8.29	9.36	-2.09	0.00E+00	0.00E+00	gb|AE004091|AE004091:576037-	Biosynthesis of	P95412
PA0512_at	PA0512	5.78	7.25	-2.76	0.00E+00	0.00E+00	gb|AE004091|AE004091:574590-	Biosynthesis of	P95415
PA0510_at	PA0510	6.77	9.44	-6.33	0.00E+00	0.00E+00	gb|AE004091|AE004091:572583-	Biosynthesis of	P95417; G3XD80
PA0573_at	PA0573	5.67	9.12	-10.91	0.00E+00	0.00E+00	gb|AE004091|AE004091:628335-628671 (-) // 1(		Q9I5W3
PA3294_s_at	PA3294	8.46	9.53	-2.1	0.00E+00	0.00E+00	gb|AE004091|AE004091:3900230-3902157 (+) /		Q9HYU9; Q9HYC3
PA1638_at	PA1638	9.57	7.98	3.01	0.00E+00	0.00E+00	gb|AE004091|AE004091:1784108-1785017 (+) /		Q9I387
PA1653_at	PA1653	6.14	8.24	-4.29	0.00E+00	0.00E+00	gb|AE004091|AE004091:1800071-1800548 (-) //		Q9I372
PA2607_at	PA2607	6.45	8.69	-4.72	0.00E+00	0.00E+00	gb|AE004091|AE004091:2949332-2949638 (+) /		Q9I0N1
PA2627_at	PA2627	8.8	7.61	2.29	0.00E+00	0.00E+00	gb|AE004091|AE004091:2971110-2971731 (+) /		Q9I0L1
PA2653_at	PA2653	7.95	9.28	-2.51	0.00E+00	0.00E+00	gb|AE004091|AE004091:3001872-3003198 (-) //		Q9I0I5
PA2655_i_at	PA2655	6.66	5.19	2.78	0.00E+00	0.00E+00	gb|AE004091|AE004091:3005605-3005914 (-) //		Q9I0I3
PA2658_at	PA2658	9.02	10.19	-2.24	0.00E+00	0.00E+00	gb|AE004091|AE004091:3008009-3008324 (-) //		Q9I0I0
PA2660_at	PA2660	6.03	8.31	-4.87	0.00E+00	0.00E+00	gb|AE004091|AE004091:3008846-3009929 (+) /		Q9I0H8
PA2665_at	PA2665	6.87	8.5	-3.11	0.00E+00	0.00E+00	gb|AE004091|AE004091:3013927-3015481 (+) /		Q9I0H3
PA2666_at	PA2666	5.76	8.31	-5.87	0.00E+00	0.00E+00	gb|AE004091|AE004091:3015581-3015938 (+) /		Q9I0H2
PA2670_at	PA2670	7.41	9.02	-3.06	0.00E+00	0.00E+00	gb|AE004091|AE004091:3017817-3018804 (-) //		Q9I0G8
PA2680_at	PA2680	5.89	7.79	-3.72	0.00E+00	0.00E+00	gb|AE004091|AE004091:3027077-3028052 (+) /		Q9I0F8
PA2682_at	PA2682	6.04	9.23	-9.1	0.00E+00	0.00E+00	gb|AE004091|AE004091:3029186-3030428 (+) /		Q9I0F6
PA2692_at	PA2692	8.45	9.55	-2.15	0.00E+00	0.00E+00	gb|AE004091|AE004091:3046420-3046945 (+) /		Q9I0F0
PA2704_at	PA2704	6.52	8.1	-3	0.00E+00	0.00E+00	gb|AE004091|AE004091:3057901-3058921 (-) //		Q9I0D8
PA2708_at	PA2708	6.65	8.37	-3.28	0.00E+00	0.00E+00	gb|AE004091|AE004091:3061732-3062818 (-) //		Q9I0D4
PA2592_at	PA2592	9.05	7.72	2.5	0.00E+00	0.00E+00	gb|AE004091|AE004091:2934540-2935644 (+) /		Q9I0P5
PA2719_at	PA2719	6.76	9.19	-5.38	0.00E+00	0.00E+00	gb|AE004091|AE004091:3073731-3074418 (+) /		Q9I0C3
PA2727_at	PA2727	6.48	8.08	-3.03	0.00E+00	0.00E+00	gb|AE004091|AE004091:3079849-3083482 (+) // 100.0 //		
PA2764_at	PA2764	6.24	8.94	-6.51	0.00E+00	0.00E+00	gb|AE004091|AE004091:3123598-3124294 (+) /		Q9I079
PA2765_at	PA2765	6.45	8.99	-5.8	0.00E+00	0.00E+00	gb|AE004091|AE004091:3124341-3125241 (-) //		Q9I078
PA2767_at	PA2767	7.46	8.55	-2.14	0.00E+00	0.00E+00	gb|AE004091|AE004091:3126251-3127220 (+) /		Q9I076

PA2770_at	PA2770	7.63	9.28	-3.14	0.00E+00	0.00E+00	gb|AE004091|AE004091:3128291-3129071 (-) // Q9I073
PA2771_at	PA2771	6.45	8.91	-5.51	0.00E+00	0.00E+00	gb|AE004091|AE004091:3129728-3130754 (+) / Q9I072
PA2776_at	PA2776	6.19	7.45	-2.38	0.00E+00	0.00E+00	gb|AE004091|AE004091:3133709-3134993 (+) / Q9I067
PA2784_at	PA2784	7.16	9.5	-5.06	0.00E+00	0.00E+00	gb|AE004091|AE004091:3141718-3142276 (+) / Q9I059
PA2785_at	PA2785	7.09	9.27	-4.56	0.00E+00	0.00E+00	gb|AE004091|AE004091:3142284-3142503 (+) / Q9I058
PA2791_at	PA2791	9.37	8.31	2.08	0.00E+00	0.00E+00	gb|AE004091|AE004091:3148338-3148629 (-) // Q9I052
PA2793_at	PA2793	7.21	8.96	-3.35	0.00E+00	0.00E+00	gb|AE004091|AE004091:3149399-3150434 (-) // Q9I050
PA2797_at	PA2797	7.01	8.94	-3.81	0.00E+00	0.00E+00	gb|AE004091|AE004091:3154592-3155075 (-) // Q9I046
PA2798_at	PA2798	5.63	8.77	-8.83	0.00E+00	0.00E+00	gb|AE004091|AE004091:315507: Two-componen Q9I045
PA2799_at	PA2799	6.94	8.91	-3.93	0.00E+00	0.00E+00	gb|AE004091|AE004091:3156502-3156802 (+) / Q9I044
PA2721_at	PA2721	6.21	7.79	-2.99	0.00E+00	0.00E+00	gb|AE004091|AE004091:3075410-3075890 (+) / Q9I0C1
PA2800_at	PA2800	6.69	9.81	-8.71	0.00E+00	0.00E+00	gb|AE004091|AE004091:3156859-3157564 (-) // Q9I043
PA2572_at	PA2572	8.08	9.11	-2.04	0.00E+00	0.00E+00	gb|AE004091|AE004091:290765! Two-componen Q9I0R4
PA2557_at	PA2557	7.49	8.66	-2.26	0.00E+00	0.00E+00	gb|AE004091|AE004091:2890355-2892050 (-) // Q9I0S7
PA2408_at	PA2408	7.04	8.33	-2.44	0.00E+00	0.00E+00	gb|AE004091|AE004091:2691109-2691865 (+) / Q9I173
PA2417_at	PA2417	8.04	6.3	3.35	0.00E+00	0.00E+00	gb|AE004091|AE004091:2700166-2701105 (-) // Q9I164
PA2418_at	PA2418	7.58	9.54	-3.89	0.00E+00	0.00E+00	gb|AE004091|AE004091:2701205-2702066 (+) / Q9I163
PA2439_at	PA2439	5.44	6.55	-2.16	0.00E+00	0.00E+00	gb|AE004091|AE004091:2735193-2737194 (-) // Q9I143
PA2441_at	PA2441	6.53	8.33	-3.48	0.00E+00	0.00E+00	gb|AE004091|AE004091:2738840-2739716 (+) / Q9I141
PA2449_at	PA2449	5.6	7.35	-3.38	0.00E+00	0.00E+00	gb|AE004091|AE004091:2750127-2751663 (-) // Q9I133
PA2452_at	PA2452	6.22	8.3	-4.23	0.00E+00	0.00E+00	gb|AE004091|AE004091:2753518-2754445 (-) // 100.0 //
PA2459_at	PA2459	5.45	6.47	-2.03	0.00E+00	0.00E+00	gb|AE004091|AE004091:2759481-2760087 (-) // Q9I123
PA2460_i_at	PA2460	9.69	10.87	-2.27	0.00E+00	0.00E+00	gb|AE004091|AE004091:2760322-2760619 (-) // Q9I122
PA2461_at	PA2461	7.82	4.8	8.11	0.00E+00	0.00E+00	gb|AE004091|AE004091:2760871-2761351 (-) // Q9I121
PA2465_at	PA2465	5.36	8.63	-9.6	0.00E+00	0.00E+00	gb|AE004091|AE004091:2781497-2782646 (-) // Q9I117
PA2468_at	PA2468	5.61	7.39	-3.44	0.00E+00	0.00E+00	gb|AE004091|AE004091:2786364-2786883 (-) // Q9I114
PA2474_at	PA2474	7.73	8.81	-2.12	0.00E+00	0.00E+00	gb|AE004091|AE004091:2791905-2792817 (+) / Q9I108
PA2478_at	PA2478	8.83	6.66	4.51	0.00E+00	0.00E+00	gb|AE004091|AE004091:2795808-2797572 (-) // Q9I104
PA2558_at	PA2558	6.98	8.02	-2.05	0.00E+00	0.00E+00	gb|AE004091|AE004091:2892270-2892963 (+) / Q9I0S6
PA2480_at	PA2480	6.71	4.59	4.36	0.00E+00	0.00E+00	gb|AE004091|AE004091:279841: Two-componen Q9I102
PA2489_at	PA2489	6.3	8.69	-5.23	0.00E+00	0.00E+00	gb|AE004091|AE004091:2805020-2805836 (+) / Q9I0Z3
PA2490_at	PA2490	9.43	7.71	3.31	0.00E+00	0.00E+00	gb|AE004091|AE004091:2805916-2806291 (+) / Q9I0Z2
PA2491_at	PA2491	7.15	8.43	-2.43	0.00E+00	0.00E+00	gb|AE004091|AE004091:2806349-2807369 (-) // Q9I0Z1
PA2498_at	PA2498	8.21	9.23	-2.03	0.00E+00	0.00E+00	gb|AE004091|AE004091:2816346-2816979 (+) / Q9I0Y4
PA2500_at	PA2500	9.39	7.24	4.43	0.00E+00	0.00E+00	gb|AE004091|AE004091:2817448-2818675 (+) / Q9I0Y2
PA2504_at	PA2504	8.25	9.36	-2.16	0.00E+00	0.00E+00	gb|AE004091|AE004091:2821704-2822322 (-) // Q9I0X8
PA2511_at	PA2511	9.66	8.07	3.02	0.00E+00	0.00E+00	gb|AE004091|AE004091:2828121-2829123 (-) // Q9I0X1
PA2529_at	PA2529	7.5	8.59	-2.13	0.00E+00	0.00E+00	gb|AE004091|AE004091:2855547-2856981 (+) / Q9I0V4

PA2535_at	PA2535	9.39	8.03	2.56	0.00E+00	0.00E+00	gb|AE004091|AE004091:2862943-2863939 (+) /, Q9I0U9
PA2537_at	PA2537	8.82	7.55	2.41	0.00E+00	0.00E+00	gb|AE004091|AE004091:2865106-2865736 (-) //, Q9I0U7
PA2540_at	PA2540	8.68	7.33	2.55	0.00E+00	0.00E+00	gb|AE004091|AE004091:2867541-2869302 (-) //, Q9I0U4
PA2546_at	PA2546	7.33	9.17	-3.59	0.00E+00	0.00E+00	gb|AE004091|AE004091:2877476-2877908 (-) //, Q9I0T8
PA2550_at	PA2550	7.77	8.83	-2.09	0.00E+00	0.00E+00	gb|AE004091|AE004091:2881753-2882983 (-) //, Q9I0T4
PA2553_at	PA2553	7.96	6.31	3.14	0.00E+00	0.00E+00	gb|AE004091|AE004091:2885361-2886552 (-) //, Q9I0T1
PA2486_at	PA2486	5.57	7.04	-2.78	0.00E+00	0.00E+00	gb|AE004091|AE004091:2803639-2803834 (+) /, Q9I0Z6
PA2402_at	PA2402	7.22	8.64	-2.67	0.00E+00	0.00E+00	gb|AE004091|AE004091:2671728-2687178 (-) //, Q9I179
PA2808_i_at	PA2808	4.58	8.52	-15.35	0.00E+00	0.00E+00	gb|AE004091|AE004091:3162387-3162579 (-) //, Q9I035
PA2813_at	PA2813	8.31	9.5	-2.28	0.00E+00	0.00E+00	gb|AE004091|AE004091:3166546-3167167 (-) //, Q9I030
PA3070_at	PA3070	9.55	8.48	2.1	0.00E+00	0.00E+00	gb|AE004091|AE004091:3442822-3443803 (+) /, Q9HZD8
PA3080_at	PA3080	7.1	9.28	-4.55	0.00E+00	0.00E+00	gb|AE004091|AE004091:3455265-3456363 (-) //, Q9HZC8
PA3088_at	PA3088	6.69	8.01	-2.51	0.00E+00	0.00E+00	gb|AE004091|AE004091:3466072-3466960 (-) //, Q9HZC0
PA3091_at	PA3091	7.2	8.85	-3.13	0.00E+00	0.00E+00	gb|AE004091|AE004091:3468987-3470427 (-) //, Q9HZB7
PA3094_at	PA3094	9.94	8.83	2.16	0.00E+00	0.00E+00	gb|AE004091|AE004091:3474132-3475170 (+) /, Q9HZB4
PA3125_at	PA3125	7.63	9.13	-2.82	0.00E+00	0.00E+00	gb|AE004091|AE004091:3507344-3508673 (+) /, Q9HZ99
PA3130_at	PA3130	8.01	5.9	4.31	0.00E+00	0.00E+00	gb|AE004091|AE004091:3511956-3512394 (-) //, Q9HZ94
PA3140_at	PA3140	6.06	8.6	-5.81	0.00E+00	0.00E+00	gb|AE004091|AE004091:3524160-3524490 (-) //, Q9HZ87
PA3144_f_at	PA3144	7.07	4.74	5.05	0.00E+00	0.00E+00	gb|AE004091|AE004091:3528230-3528350 (-) // 100.0 //
PA3179_at	PA3179	7.62	9.32	-3.25	0.00E+00	0.00E+00	gb|AE004091|AE004091:3569105-3570266 (-) //, Q9HZ55
PA3181_at	PA3181	5.94	7.67	-3.32	0.00E+00	0.00E+00	gb|AE004091|AE004091:357094(Glyoxylate and O68283
PA3185_at	PA3185	9.16	8.1	2.07	0.00E+00	0.00E+00	gb|AE004091|AE004091:3574845-3575697 (-) //, Q9HZ52
PA3189_at	PA3189	7.26	9	-3.35	0.00E+00	0.00E+00	gb|AE004091|AE004091:3579350-3580283 (-) //, Q9HZ49
PA3191_at	PA3191	5.92	8.02	-4.28	0.00E+00	0.00E+00	gb|AE004091|AE004091:358217(Two-componer Q9HZ47
PA3055_at	PA3055	10.56	9.55	2.01	0.00E+00	0.00E+00	gb|AE004091|AE004091:3420308-3420785 (-) //, Q9HZF3
PA3199_at	PA3199	7.27	8.67	-2.63	0.00E+00	0.00E+00	gb|AE004091|AE004091:3591414-3592044 (-) //, Q9HZ41
PA3218_at	PA3218	7.28	9.16	-3.68	0.00E+00	0.00E+00	gb|AE004091|AE004091:3607616-3608090 (+) // 100.0 //
PA3226_at	PA3226	9.88	8.76	2.18	0.00E+00	0.00E+00	gb|AE004091|AE004091:3613494-3614322 (+) /, Q9HZ14
PA3232_at	PA3232	7.68	8.72	-2.05	0.00E+00	0.00E+00	gb|AE004091|AE004091:3618992-3619619 (-) //, Q9HZ08
PA3236_at	PA3236	9.52	8.5	2.03	0.00E+00	0.00E+00	gb|AE004091|AE004091:3623685-3624543 (-) //, Q9HZ04
PA3238_at	PA3238	6.11	8.33	-4.66	0.00E+00	0.00E+00	gb|AE004091|AE004091:3625200-3626562 (+) /, Q9HZ02
PA3240_at	PA3240	8.4	9.43	-2.04	0.00E+00	0.00E+00	gb|AE004091|AE004091:3627508-3628366 (+) /, Q9HZ00
PA3241_at	PA3241	5.81	8.06	-4.76	0.00E+00	0.00E+00	gb|AE004091|AE004091:3628429-3629599 (-) //, Q9HYZ9
PA3253_at	PA3253	6.95	8.57	-3.06	0.00E+00	0.00E+00	gb|AE004091|AE004091:3639365-3640163 (+) /, Q9HYY7
PA3261_at	PA3261	7.5	8.55	-2.06	0.00E+00	0.00E+00	gb|AE004091|AE004091:3648169-3648916 (+) /, Q9HYX9
PA3269_at	PA3269	6.26	8.28	-4.06	0.00E+00	0.00E+00	gb|AE004091|AE004091:3658247-3659102 (-) //, Q9HYX2
PA3270_at	PA3270	9.9	8.8	2.14	0.00E+00	0.00E+00	gb|AE004091|AE004091:3659168-3659756 (-) //, Q9HYX1
PA3274_at	PA3274	6.37	8.94	-5.94	0.00E+00	0.00E+00	gb|AE004091|AE004091:3668473-3668761 (+) /, Q9HYW7

PA3276_at	PA3276	7.47	8.96	-2.81	0.00E+00	0.00E+00	gb|AE004091|AE004091:3669165-3669588 (-) // Q9HYW5
PA3283_at	PA3283	7.6	8.88	-2.44	0.00E+00	0.00E+00	gb|AE004091|AE004091:3675893-3676748 (-) // Q9HYW0
PA3214_at	PA3214	5.99	8.49	-5.65	0.00E+00	0.00E+00	gb|AE004091|AE004091:3603326-3603971 (+) / Q9HZ26
PA2812_at	PA2812	8.57	10.13	-2.96	0.00E+00	0.00E+00	gb|AE004091|AE004091:3165538-3166471 (-) // Q9I031
PA3052_at	PA3052	7.43	9.13	-3.25	0.00E+00	0.00E+00	gb|AE004091|AE004091:3416060-3417041 (+) / Q9HZF6
PA3047_at	PA3047	9.6	8.51	2.14	0.00E+00	0.00E+00	gb|AE004091|AE004091:341026: Peptideglycan I Q9HZG1
PA2814_at	PA2814	8.16	6.04	4.37	0.00E+00	0.00E+00	gb|AE004091|AE004091:3167281-3167953 (-) // Q9I029
PA2816_i_at	PA2816	8.8	5.9	7.46	0.00E+00	0.00E+00	gb|AE004091|AE004091:3170681-3171062 (+) / Q9I027
PA2820_at	PA2820	6.04	8.56	-5.75	0.00E+00	0.00E+00	gb|AE004091|AE004091:3174130-3174904 (+) / Q9I023
PA2829_at	PA2829	4.51	8.33	-14.12	0.00E+00	0.00E+00	gb|AE004091|AE004091:3182399-3182852 (+) / Q9I014
PA2836_at	PA2836	4.55	7.81	-9.61	0.00E+00	0.00E+00	gb|AE004091|AE004091:3189149-3190214 (+) / Q9I007
PA2837_at	PA2837	6.93	8.53	-3.03	0.00E+00	0.00E+00	gb|AE004091|AE004091:3190210-3191650 (+) / Q9I006
PA2857_at	PA2857	7.33	8.79	-2.75	0.00E+00	0.00E+00	gb|AE004091|AE004091:3209288-3209972 (+) / Q9HZY7
PA2864_at	PA2864	5.34	8.88	-11.59	0.00E+00	0.00E+00	gb|AE004091|AE004091:3216455-3216890 (-) // Q9HZY2
PA2865_at	PA2865	6.61	8.07	-2.76	0.00E+00	0.00E+00	gb|AE004091|AE004091:3216988-3218419 (-) // Q9HZY1
PA2873_at	PA2873	8.21	5.56	6.3	0.00E+00	0.00E+00	gb|AE004091|AE004091:3225380-3227387 (-) // Q9HZX3
PA2881_at	PA2881	9.32	8.25	2.1	0.00E+00	0.00E+00	gb|AE004091|AE004091:323334: Two-componer Q9HZW6
PA2884_at	PA2884	8.6	9.7	-2.14	0.00E+00	0.00E+00	gb|AE004091|AE004091:3236193-3236958 (+) / Q9HZW3
PA2890_at	PA2890	9.92	8.64	2.42	0.00E+00	0.00E+00	gb|AE004091|AE004091:3243504-3244299 (+) / Q9HZV7
PA2902_at	PA2902	8.95	7.82	2.19	0.00E+00	0.00E+00	gb|AE004091|AE004091:3255762-3256611 (-) // Q9HZU5
PA3048_at	PA3048	7.46	9.24	-3.43	0.00E+00	0.00E+00	gb|AE004091|AE004091:3411762-3413940 (-) // Q9HZG0
Pae_AF241171cds47	Pae_AF241171	4.55	2.23	4.99	0.00E+00	0.00E+00	
PA2927_at	PA2927	6.49	8.38	-3.7	0.00E+00	0.00E+00	gb|AE004091|AE004091:3281987-3283319 (+) / Q9HZS0
PA2928_at	PA2928	10.32	9.21	2.16	0.00E+00	0.00E+00	gb|AE004091|AE004091:3283374-3284598 (-) // Q9HZR9
PA2947_i_at	PA2947	6.89	9.05	-4.49	0.00E+00	0.00E+00	gb|AE004091|AE004091:330708 Cobalamin bios Q9HZQ0
PA2987_at	PA2987	9.55	8.32	2.34	0.00E+00	0.00E+00	gb|AE004091|AE004091:3345112-3345796 (-) // Q9HZL7
PA2988_at	PA2988	5.36	7.13	-3.41	0.00E+00	0.00E+00	gb|AE004091|AE004091:3345788-3347039 (-) // Q9HZL6
PA2992_at	PA2992	8.53	9.97	-2.71	0.00E+00	0.00E+00	gb|AE004091|AE004091:3350410-3350638 (-) // Q9HZL3
PA3001_at	PA3001	7.55	9.07	-2.87	0.00E+00	0.00E+00	gb|AE004091|AE004091:335926 Glycolysis / Glu Q9HZK4
PA3004_at	PA3004	6.91	8.04	-2.2	0.00E+00	0.00E+00	gb|AE004091|AE004091:3365006-3365744 (-) // 100.0 //
PA3018_at	PA3018	7.22	9.52	-4.92	0.00E+00	0.00E+00	gb|AE004091|AE004091:3378978-3379665 (-) // Q9HZI8
PA3022_at	PA3022	9.66	8.35	2.48	0.00E+00	0.00E+00	gb|AE004091|AE004091:3384377-3385184 (-) // Q9HZI4
PA3024_at	PA3024	6.72	7.85	-2.19	0.00E+00	0.00E+00	gb|AE004091|AE004091:3386269-3387829 (-) // Q9HZI2
PA3027_at	PA3027	7.49	8.86	-2.58	0.00E+00	0.00E+00	gb|AE004091|AE004091:3391207-3392239 (+) / Q9HZH9
PA3033_at	PA3033	7.01	9.84	-7.1	0.00E+00	0.00E+00	gb|AE004091|AE004091:3397095-3397374 (-) // Q9HZH5
PA3036_at	PA3036	6.99	8.93	-3.82	0.00E+00	0.00E+00	gb|AE004091|AE004091:3398716-3399649 (+) // 100.0 //
PA2919_at	PA2919	7.2	8.66	-2.75	0.00E+00	0.00E+00	gb|AE004091|AE004091:3274327-3274585 (+) / Q9HZS8
PA2387_at	PA2387	5.59	8.21	-6.15	0.00E+00	0.00E+00	gb|AE004091|AE004091:2640392-2640872 (-) // Q9I193

PA2378_at	PA2378	6.3	8.85	-5.86	0.00E+00	0.00E+00	gb|AE004091|AE004091:2629916-2632232 (-) //	Q9I1A1
PA2376_at	PA2376	8.81	7.75	2.09	0.00E+00	0.00E+00	gb|AE004091|AE004091:2627452-2628094 (+) /,	Q9I1A3
PA1872_at	PA1872	9.55	8.33	2.34	0.00E+00	0.00E+00	gb|AE004091|AE004091:2034066-2034858 (-) //	G3XD32
PA1876_at	PA1876	6.26	7.99	-3.31	0.00E+00	0.00E+00	gb|AE004091|AE004091:2045114-2047286 (+) /,	Q9I2M1
PA1877_at	PA1877	5.8	8.67	-7.3	0.00E+00	0.00E+00	gb|AE004091|AE004091:2047275-2048463 (+) /,	Q9I2M0
PA1879_at	PA1879	9.63	8.53	2.14	0.00E+00	0.00E+00	gb|AE004091|AE004091:2049237-2049792 (-) //	Q9I2L8
PA1884_at	PA1884	8.96	7.43	2.88	0.00E+00	0.00E+00	gb|AE004091|AE004091:2053672-2054224 (-) //	Q9I2L3
PA1887_at	PA1887	7.89	8.96	-2.11	0.00E+00	0.00E+00	gb|AE004091|AE004091:2057278-2057947 (-) //	Q9I2L0
PA1893_at	PA1893	8.12	9.17	-2.07	0.00E+00	0.00E+00	gb|AE004091|AE004091:2062398-2064828 (-) //	Q9I2K4
PA1907_at	PA1907	6.62	8.71	-4.24	0.00E+00	0.00E+00	gb|AE004091|AE004091:2077626-2079267 (-) //	Q9I2J7
PA1911_at	PA1911	6.95	8.32	-2.57	0.00E+00	0.00E+00	gb|AE004091|AE004091:2084476-2085427 (-) //	Q9I2J3
PA1915_at	PA1915	6.97	8.3	-2.51	0.00E+00	0.00E+00	gb|AE004091|AE004091:2088602-2090150 (-) //	Q9I2I9
PA1916_at	PA1916	9.93	8.45	2.79	0.00E+00	0.00E+00	gb|AE004091|AE004091:2090213-2091446 (-) //	Q9I2I8
PA1917_at	PA1917	6.39	7.76	-2.59	0.00E+00	0.00E+00	gb|AE004091|AE004091:2091490-2091838 (-) //	Q9I2I7
PA1922_at	PA1922	7.09	8.1	-2.02	0.00E+00	0.00E+00	gb|AE004091|AE004091:2097491-2099453 (+) /,	Q9I2I2
PA1925_at	PA1925	9.28	8.2	2.12	0.00E+00	0.00E+00	gb|AE004091|AE004091:2103770-2104097 (+) /,	Q9I2H9
PA1869_at	PA1869	7.39	9.21	-3.55	0.00E+00	0.00E+00	gb|AE004091|AE004091:2031466-2031706 (+) /,	O52658
PA1926_at	PA1926	6.7	8.93	-4.71	0.00E+00	0.00E+00	gb|AE004091|AE004091:2104597-2106448 (+) /,	Q9I2H8
PA1932_at	PA1932	7.84	9.02	-2.26	0.00E+00	0.00E+00	gb|AE004091|AE004091:211220(Aromatic comp	Q9I2H2
PA1934_at	PA1934	9.83	8.79	2.06	0.00E+00	0.00E+00	gb|AE004091|AE004091:2115885-2116266 (+) /,	Q9I2H0
PA1951_at	PA1951	7.44	5.97	2.76	0.00E+00	0.00E+00	gb|AE004091|AE004091:2136520-2137786 (-) //	Q9I2F3
PA1952_at	PA1952	9.45	8.09	2.57	0.00E+00	0.00E+00	gb|AE004091|AE004091:2137846-2138599 (-) //	Q9I2F2
PA1953_at	PA1953	7.76	5.17	6.01	0.00E+00	0.00E+00	gb|AE004091|AE004091:2138612-2139293 (-) //	Q9I2F1
PA1962_at	PA1962	7.69	8.76	-2.1	0.00E+00	0.00E+00	gb|AE004091|AE004091:2145894-2146503 (+) /,	Q9I2E2
PA1972_at	PA1972	5.18	6.61	-2.69	0.00E+00	0.00E+00	gb|AE004091|AE004091:2153594-2155298 (+) /,	Q9I2D3
PA1975_at	PA1975	7.64	9.25	-3.06	0.00E+00	0.00E+00	gb|AE004091|AE004091:2159270-2160434 (+) /,	Q9I2D0
PA1976_at	PA1976	6.73	8.75	-4.06	0.00E+00	0.00E+00	gb|AE004091|AE004091:216036: Two-componer	Q9I2C9
PA1991_at	PA1991	6.34	8.71	-5.16	0.00E+00	0.00E+00	gb|AE004091|AE004091:2176974-2178138 (+) /,	Q9I2B8
PA1993_at	PA1993	8.57	7.42	2.22	0.00E+00	0.00E+00	gb|AE004091|AE004091:2179848-2181057 (-) //	Q9I2B6
PA2006_at	PA2006	9.37	8.23	2.2	0.00E+00	0.00E+00	gb|AE004091|AE004091:219405: Aromatic comp	Q9I2A3
PA2018_at	PA2018	9.52	8.43	2.14	0.00E+00	0.00E+00	gb|AE004091|AE004091:2208169-2211307 (-) //	Q9RG59; G3XCW2
PA2019_at	PA2019	10.14	9.03	2.16	0.00E+00	0.00E+00	gb|AE004091|AE004091:2211322-2212513 (-) //	Q9RG60; G3XD21
PA1930_at	PA1930	9.41	8.36	2.07	0.00E+00	0.00E+00	gb|AE004091|AE004091:211022(Chemotactic tr	Q9I2H4
PA2024_at	PA2024	8.09	9.15	-2.08	0.00E+00	0.00E+00	gb|AE004091|AE004091:221612: Aromatic comp	P23205
PA1853_at	PA1853	7.71	9.17	-2.77	0.00E+00	0.00E+00	gb|AE004091|AE004091:2012815-2013679 (+) /,	Q9I2P2
PA1848_at	PA1848	5.94	7.95	-4.02	0.00E+00	0.00E+00	gb|AE004091|AE004091:2008308-2009478 (-) //	Q9I2P7
PA1654_at	PA1654	7.32	8.64	-2.5	0.00E+00	0.00E+00	gb|AE004091|AE004091:1800629-1801796 (+) /,	Q9I371
PA1655_at	PA1655	10.11	8.98	2.19	0.00E+00	0.00E+00	gb|AE004091|AE004091:1801851-1802454 (+) /,	Q9I370

PA1666_at	PA1666	9.79	7.52	4.82	0.00E+00	0.00E+00	gb|AE004091|AE004091:1816352-1816859 (+) /		Q9I359
PA1667_at	PA1667	9.06	6.58	5.57	0.00E+00	0.00E+00	gb|AE004091|AE004091:1816855-1818187 (+) /		Q9I358
PA1668_at	PA1668	7.01	9.22	-4.63	0.00E+00	0.00E+00	gb|AE004091|AE004091:1818189-1819059 (+) /		Q9I357
PA1669_at	PA1669	9.62	8.17	2.74	0.00E+00	0.00E+00	gb|AE004091|AE004091:1819074-1822602 (+) /		Q9I356
PA1672_at	PA1672	6.43	8.9	-5.55	0.00E+00	0.00E+00	gb|AE004091|AE004091:1824343-1824724 (-) //		Q9I353
PA1678_at	PA1678	6.27	9.19	-7.57	0.00E+00	0.00E+00	gb|AE004091|AE004091:1827992-1828907 (+) /		Q9I347
PA1683_at	PA1683	8.32	9.49	-2.25	0.00E+00	0.00E+00	gb|AE004091|AE004091:1833162-1833780 (+) /		Q9I342
PA1684_at	PA1684	7.05	8.64	-3.01	0.00E+00	0.00E+00	gb|AE004091|AE004091:1833776-1834322 (+) /		Q9I341
PA1688_at	PA1688	5.78	8.16	-5.19	0.00E+00	0.00E+00	gb|AE004091|AE004091:1837388-1838258 (+) /		G3XD03
PA1699_at	PA1699	6.31	4.88	2.7	0.00E+00	0.00E+00	gb|AE004091|AE004091:184807	Type III Secreti	G3XCT8
PA1700_at	PA1700	10.35	8.96	2.61	0.00E+00	0.00E+00	gb|AE004091|AE004091:184833	Type III Secreti	Q9I329
PA1701_at	PA1701	8.58	9.6	-2.02	0.00E+00	0.00E+00	gb|AE004091|AE004091:184870	Type III Secreti	G3XD37
PA1850_at	PA1850	9.2	7.11	4.27	0.00E+00	0.00E+00	gb|AE004091|AE004091:2009747-2010752 (-) //		Q9I2P5
PA1729_at	PA1729	10.27	9.21	2.08	0.00E+00	0.00E+00	gb|AE004091|AE004091:1871555-1872203 (+) /		Q9I308
PA1751_at	PA1751	8.43	6.25	4.54	0.00E+00	0.00E+00	gb|AE004091|AE004091:1892133-1892511 (+) /		Q9I2Y6
PA1755_at	PA1755	7.97	9.43	-2.75	0.00E+00	0.00E+00	gb|AE004091|AE004091:1895280-1895628 (-) //		Q9I2Y3
PA1760_at	PA1760	6.89	8.54	-3.13	0.00E+00	0.00E+00	gb|AE004091|AE004091:1901524-1904248 (+) /		Q9I2X9
PA1762_at	PA1762	5.67	8.61	-7.72	0.00E+00	0.00E+00	gb|AE004091|AE004091:1904995-1905757 (+) /		Q9I2X7
PA1765_at	PA1765	9.19	7.77	2.67	0.00E+00	0.00E+00	gb|AE004091|AE004091:1908454-1909642 (+) /		Q9I2X4
PA1771_at	PA1771	5.88	8.44	-5.88	0.00E+00	0.00E+00	gb|AE004091|AE004091:1916482-1917493 (+) /		Q9I2W8
PA1779_at	PA1779	4.3	6.94	-6.22	0.00E+00	0.00E+00	gb|AE004091|AE004091:192304	Nitrogen metal	Q9I2W3
PA1786_at	PA1786	6.96	8.59	-3.09	0.00E+00	0.00E+00	gb|AE004091|AE004091:193364	Nitrogen metal	Q9I2V6
PA1817_at	PA1817	5.78	7.16	-2.61	0.00E+00	0.00E+00	gb|AE004091|AE004091:1974238-1974628 (-) //		Q9I2S8
PA1824_at	PA1824	8.27	7.18	2.12	0.00E+00	0.00E+00	gb|AE004091|AE004091:1983509-1984289 (-) //		Q9I2S1
PA1826_at	PA1826	6.91	8.63	-3.28	0.00E+00	0.00E+00	gb|AE004091|AE004091:1985030-1985936 (-) //		Q9I2R9
PA1828_at	PA1828	7.35	4.92	5.4	0.00E+00	0.00E+00	gb|AE004091|AE004091:1986989-1987757 (-) //		Q9I2R7
PA1829_at	PA1829	7.68	9.17	-2.8	0.00E+00	0.00E+00	gb|AE004091|AE004091:1987781-1988852 (-) //		Q9I2R6
PA1839_at	PA1839	9.25	7.75	2.83	0.00E+00	0.00E+00	gb|AE004091|AE004091:1997509-1998550 (-) //		Q9I2Q6
PA1738_at	PA1738	6.2	8.59	-5.23	0.00E+00	0.00E+00	gb|AE004091|AE004091:1882595-1883510 (-) //		Q9I2Z9
PA2029_i_at	PA2029	4.91	5.96	-2.07	0.00E+00	0.00E+00	gb|AE004091|AE004091:2220275-2220575 (+) /		Q9I286
PA2035_at	PA2035	8.44	4.36	16.86	0.00E+00	0.00E+00	gb|AE004091|AE004091:2224486-2226145 (-) //		Q9I280
PA2044_at	PA2044	9.8	8.77	2.03	0.00E+00	0.00E+00	gb|AE004091|AE004091:2236492-2238367 (-) //		Q9I271
PA2215_at	PA2215	6.28	8.12	-3.57	0.00E+00	0.00E+00	gb|AE004091|AE004091:2435101-2436277 (+) /		Q9I1Q2
PA2217_at	PA2217	5.17	8.67	-11.29	0.00E+00	0.00E+00	gb|AE004091|AE004091:2437428-2439012 (+) /		Q9I1Q0
PA2221_at	PA2221	6.58	8.34	-3.37	0.00E+00	0.00E+00	gb|AE004091|AE004091:2443161-2444367 (+) /		Q9I1P8
PA2225_at	PA2225	9.32	8.28	2.06	0.00E+00	0.00E+00	gb|AE004091|AE004091:2447573-2447990 (-) //		Q9I1P4
PA2226_at	PA2226	8.46	7.27	2.28	0.00E+00	0.00E+00	gb|AE004091|AE004091:2448033-2448534 (-) //		Q9I1P3
PA2228_at	PA2228	8.75	7.31	2.71	0.00E+00	0.00E+00	gb|AE004091|AE004091:2449554-2450766 (-) //		Q9I1P1

PA2261_at	PA2261	5.53	8.37	-7.14	0.00E+00	0.00E+00	gb|AE004091|AE004091:2489725-2490676 (+) / Q9I1L2
PA2266_at	PA2266	6.01	7.44	-2.68	0.00E+00	0.00E+00	gb|AE004091|AE004091:2495724-2497044 (+) / Q9I1K7
PA2275_at	PA2275	6.71	8.69	-3.96	0.00E+00	0.00E+00	gb|AE004091|AE004091:2504375-2505437 (-) // Q9I1J9
PA2281_at	PA2281	7	8.07	-2.1	0.00E+00	0.00E+00	gb|AE004091|AE004091:2509480-2510335 (-) // Q9I1J4
PA2287_at	PA2287	9.23	8.14	2.13	0.00E+00	0.00E+00	gb|AE004091|AE004091:2515373-2516009 (+) / Q9I1I8
PA2307_at	PA2307	8.43	6.79	3.13	0.00E+00	0.00E+00	gb|AE004091|AE004091:2545769-2546636 (-) // Q9I1G8
PA2308_at	PA2308	6.26	8.14	-3.66	0.00E+00	0.00E+00	gb|AE004091|AE004091:2546652-2547501 (-) // Q9I1G7
PA2310_at	PA2310	9.45	8.12	2.52	0.00E+00	0.00E+00	gb|AE004091|AE004091:2548571-2549459 (-) // Q9I1G5
PA2213_at	PA2213	8.45	7.18	2.42	0.00E+00	0.00E+00	gb|AE004091|AE004091:2432312-2433563 (+) / Q9I1Q4
PA2317_at	PA2317	8.29	9.43	-2.2	0.00E+00	0.00E+00	gb|AE004091|AE004091:2554962-2556261 (+) / Q9I1F8
PA2324_at	PA2324	7.09	8.16	-2.09	0.00E+00	0.00E+00	gb|AE004091|AE004091:2564290-2565550 (+) / Q9I1F4
PA2331_at	PA2331	8.07	9.16	-2.13	0.00E+00	0.00E+00	gb|AE004091|AE004091:2572805-2573366 (-) // Q9I1E7
PA2336_at	PA2336	7.82	9.26	-2.72	0.00E+00	0.00E+00	gb|AE004091|AE004091:2579542-2580883 (+) / Q9I1E2
PA2341_at	PA2341	10.3	9.24	2.09	0.00E+00	0.00E+00	gb|AE004091|AE004091:2585299-2586412 (+) / Q9I1D7
PA2359_at	PA2359	6.82	8.59	-3.4	0.00E+00	0.00E+00	gb|AE004091|AE004091:2605937-2607023 (-) // Q9I1C0
PA2361_at	PA2361	9.17	7.18	3.97	0.00E+00	0.00E+00	gb|AE004091|AE004091:2608229-2612045 (-) // Q9I1B8
PA2362_at	PA2362	7.98	6.07	3.76	0.00E+00	0.00E+00	gb|AE004091|AE004091:2612041-2612800 (-) // Q9I1B7
PA2363_at	PA2363	7.24	8.97	-3.33	0.00E+00	0.00E+00	gb|AE004091|AE004091:2612817-2614149 (-) // Q9I1B6
PA2364_at	PA2364	10.69	9.58	2.16	0.00E+00	0.00E+00	gb|AE004091|AE004091:2614208-2614685 (-) // Q9I1B5
PA2366_at	PA2366	7.81	9.32	-2.84	0.00E+00	0.00E+00	gb|AE004091|AE004091:2615461-2616946 (+) / Q9I1B3
PA2369_at	PA2369	10.04	8.09	3.85	0.00E+00	0.00E+00	gb|AE004091|AE004091:2617938-2619732 (+) / Q9I1B0
PA2370_at	PA2370	8.2	9.24	-2.06	0.00E+00	0.00E+00	gb|AE004091|AE004091:2619695-2620712 (+) / Q9I1A9
PA2373_at	PA2373	7.54	9.26	-3.31	0.00E+00	0.00E+00	gb|AE004091|AE004091:2624204-2626211 (+) / Q9I1A6
PA2374_at	PA2374	5.56	8.62	-8.32	0.00E+00	0.00E+00	gb|AE004091|AE004091:2626221-2626758 (+) / Q9I1A5
PA2318_at	PA2318	6.43	8.25	-3.52	0.00E+00	0.00E+00	gb|AE004091|AE004091:2556293-2556659 (+) / Q9I1F7
PA2211_at	PA2211	8.44	6.77	3.17	0.00E+00	0.00E+00	gb|AE004091|AE004091:2430170-2431130 (+) / Q9I1Q6
PA2206_at	PA2206	6.07	8.03	-3.91	0.00E+00	0.00E+00	gb|AE004091|AE004091:2424482-2425430 (-) // Q9I1R1
PA2204_at	PA2204	6.41	9	-6.02	0.00E+00	0.00E+00	gb|AE004091|AE004091:2422819-2423626 (-) // Q9I1R3
PA2046_at	PA2046	6.76	9.07	-4.98	0.00E+00	0.00E+00	gb|AE004091|AE004091:2238860-2239268 (-) // 100.0 //
PA2057_at	PA2057	5.81	8.51	-6.49	0.00E+00	0.00E+00	gb|AE004091|AE004091:2251275-2253816 (+) / Q9I258
PA2058_at	PA2058	8.04	6.78	2.39	0.00E+00	0.00E+00	gb|AE004091|AE004091:2253819-2255628 (+) / Q9I257
PA2059_at	PA2059	6.82	8.63	-3.49	0.00E+00	0.00E+00	gb|AE004091|AE004091:2255629-2256703 (+) / Q9I256
PA2060_at	PA2060	5.72	6.86	-2.21	0.00E+00	0.00E+00	gb|AE004091|AE004091:2256704-2257721 (+) / Q9I255
PA2063_at	PA2063	9.02	7.78	2.36	0.00E+00	0.00E+00	gb|AE004091|AE004091:2260862-2262086 (+) / Q9I252
PA2076_at	PA2076	8.71	7.59	2.17	0.00E+00	0.00E+00	gb|AE004091|AE004091:2282480-2283383 (+) / Q9I239
PA2079_at	PA2079	6.71	8.96	-4.76	0.00E+00	0.00E+00	gb|AE004091|AE004091:2287523-2288930 (-) // Q9I236
PA2083_at	PA2083	8.41	9.83	-2.68	0.00E+00	0.00E+00	gb|AE004091|AE004091:229179 Aromatic comp Q9I232
PA2086_at	PA2086	6.5	9.07	-5.93	0.00E+00	0.00E+00	gb|AE004091|AE004091:2295533-2296436 (+) / Q9I229

PA2091_at	PA2091	6.6	8.31	-3.26	0.00E+00	0.00E+00	gb|AE004091|AE004091:2301752-2303036 (+) /, Q9I224
PA2101_at	PA2101	6.19	8.11	-3.77	0.00E+00	0.00E+00	gb|AE004091|AE004091:2312899-2313790 (+) /, Q9I214
PA2109_at	PA2109	5.46	8.97	-11.39	0.00E+00	0.00E+00	gb|AE004091|AE004091:2320586-2321063 (-) //, Q9I206
PA2114_at	PA2114	7.07	8.59	-2.88	0.00E+00	0.00E+00	gb|AE004091|AE004091:2324808-2326080 (-) //, Q9I201
PA2116_at	PA2116	8.79	7.37	2.68	0.00E+00	0.00E+00	gb|AE004091|AE004091:2327394-2328192 (+) /, Q9I1Z9
PA2124_at	PA2124	7.55	8.68	-2.19	0.00E+00	0.00E+00	gb|AE004091|AE004091:2336209-2337847 (+) /, Q9I1Z1
PA2126_at	PA2126	6.46	9.08	-6.15	0.00E+00	0.00E+00	gb|AE004091|AE004091:2339352-2339988 (-) //, Q9I1Y9
PA2203_at	PA2203	7.51	8.67	-2.23	0.00E+00	0.00E+00	gb|AE004091|AE004091:2422022-2422739 (-) //, Q9I1R4
PA2202_at	PA2202	5.98	8	-4.05	0.00E+00	0.00E+00	gb|AE004091|AE004091:2421343-2422021 (-) //, Q9I1R5
PA2198_at	PA2198	6.29	7.31	-2.02	0.00E+00	0.00E+00	gb|AE004091|AE004091:2417410-2417755 (+) /, Q9I1R9
PA2196_at	PA2196	6.97	8.65	-3.21	0.00E+00	0.00E+00	gb|AE004091|AE004091:2415661-2416246 (+) /, Q9I1S1
PA2183_at	PA2183	7.49	3.56	15.32	0.00E+00	0.00E+00	gb|AE004091|AE004091:2404949-2405234 (-) //, Q9I1T2
PA2181_at	PA2181	9.97	8.71	2.4	0.00E+00	0.00E+00	gb|AE004091|AE004091:2403151-2404285 (+) /, Q9I1T4
PA1641_at	PA1641	7.96	9.42	-2.76	0.00E+00	0.00E+00	gb|AE004091|AE004091:1786888-1787167 (+) /, Q9I384
PA2175_at	PA2175	7.64	8.85	-2.32	0.00E+00	0.00E+00	gb|AE004091|AE004091:2396536-2396884 (-) //, Q9I1U0
PA2160_at	PA2160	8.05	9.35	-2.46	0.00E+00	0.00E+00	gb|AE004091|AE004091:2379310-2381461 (-) //, Q9I1V5
PA2156_at	PA2156	5.5	7.89	-5.24	0.00E+00	0.00E+00	gb|AE004091|AE004091:2375804-2376542 (-) //, Q9I1V9
PA2155_at	PA2155	5.74	8.07	-5.04	0.00E+00	0.00E+00	gb|AE004091|AE004091:2374602-2375808 (-) //, Q9I1W0
PA2150_at	PA2150	7.12	8.34	-2.33	0.00E+00	0.00E+00	gb|AE004091|AE004091:2365081-2365963 (+) /, Q9I1W5
PA2143_at	PA2143	6.79	8.32	-2.88	0.00E+00	0.00E+00	gb|AE004091|AE004091:2358024-2358312 (+) /, Q9I1X2
PA2133_at	PA2133	6.94	8	-2.09	0.00E+00	0.00E+00	gb|AE004091|AE004091:2348538-2349396 (+) /, Q9I1Y2
PA2161_at	PA2161	7.75	8.85	-2.15	0.00E+00	0.00E+00	gb|AE004091|AE004091:2381473-2381779 (-) //, Q9I1V4
Pae_AF241171cds51	Pae_AF241171	5.39	2.68	6.57	0.00E+00	0.00E+00	
